# Kinetics of Phosphate Ions and Phytase Activity Production for Lactic Acid-Producing Bacteria Utilizing Milling and Whitening Stages Rice Bran as Biopolymer Substrates

**DOI:** 10.3390/biom13121770

**Published:** 2023-12-10

**Authors:** Rojarej Nunta, Julaluk Khemacheewakul, Charin Techapun, Sumeth Sommanee, Juan Feng, Su Lwin Htike, Chatchadaporn Mahakuntha, Kritsadaporn Porninta, Yuthana Phimolsiripol, Kittisak Jantanasakulwong, Churairat Moukamnerd, Masanori Watanabe, Anbarasu Kumar, Noppol Leksawasdi

**Affiliations:** 1 Center of Excellence in Agro Bio-Circular-Green Industry (Agro BCG) & Bioprocess Research Cluster (BRC), School of Agro-Industry, Faculty of Agro-Industry, Chiang Mai University, Chiang Mai 50100, Thailand; rojarej.rn@gmail.com (R.N.); julaluk.kh@cmu.ac.th (J.K.); sumeth.s@cmu.ac.th (S.S.); juan_f@cmu.ac.th (J.F.); sulwinhtike_sulwinh@cmu.ac.th (S.L.H.); chatcha143dp@gmail.com (C.M.); g.kritsadaporn@gmail.com (K.P.); yuthana.p@cmu.ac.th (Y.P.); jantanasakulwong.k@gmail.com (K.J.); 2 Division of Food Innovation and Business, Faculty of Agricultural Technology, Lampang Rajabhat University, Lampang 52100, Thailand; 3 Faculty of Agro-Industry, Chiang Mai University, Chiang Mai 50100, Thailand; cmoukamnerd@gmail.com; 4 Graduate School of Agriculture, Yamagata University, 1-23 Wakada-Machi, Tsuruoka, Yamagata 997-8555, Japan; mwata@tds1.tr.yamagata-u.ac.jp; 5 Department of Biotechnology, Periyar Maniammai Institute of Science & Technology (Deemed to be University), Thanjavur 613403, India

**Keywords:** sustainability, phytic acid, rice bran, lactic acid bacteria, solid waste

## Abstract

A study evaluated nine kinetic data and four kinetic parameters related to growth, production of various phytase activities (PE_act_), and released phosphate ion concentration ([Pi]) from five lactic acid bacteria (LAB) strains cultivated in three types of media: phytate (IP6), milling stage rice bran (MsRB), and whitening stage rice bran (WsRB). Score ranking techniques were used, combining these kinetic data and parameters to select the most suitable LAB strain for each medium across three cultivation time periods (24, 48, and 72 h). In the IP6 medium, *Lacticaseibacillus casei* TISTR 1500 exhibited statistically significant highest (*p* ≤ 0.05) normalized summation scores using a 2:1 weighting between kinetic and parameter data sets. This strain also had the statistically highest levels (*p* ≤ 0.05) of produced phosphate ion concentration ([Pi]) (0.55 g/L) at 72 h and produced extracellular specific phytase activity (ExSp-PE_act_) (0.278 U/mg_protein_) at 48 h. For the MsRB and WsRB media, *Lactiplantibacillus plantarum* TISTR 877 performed exceptionally well after 72 h of cultivation. It produced ([Pi], ExSp-PE_act_) pairs of (0.53 g/L, 0.0790 U/mg_protein_) in MsRB and (0.85 g/L, 0.0593 U/mg_protein_) in WsRB, respectively. Overall, these findings indicate the most promising LAB strains for each medium and cultivation time based on their ability to produce phosphate ions and extracellular specific phytase activity. The selection process utilized a combination of kinetic data and parameter analysis.

***Novelty:*** This is the first comprehensive report which compared the production of [Pi], [LA], other organic compounds, and various types of PE activity (PE_act_) from five LAB strains. These previously isolated microbes from traditional fermented food were cultivated in a conventional IP6 medium as control and compared with MsRB and WsRB media. The relevant thirteen kinetic data and parameters, including maximum specific (growth, glucose consumption, as well as Pi and LA formation) rates, were evaluated and could be used in the subsequent scale-up design and optimization of relevant production processes.

## 1. Introduction

Utilization of agro-industrial residues for the production of value-added products and subsequent removal of excess by-products by environmentally friendly means are necessary steps towards zero-waste valorization processes, which can be achieved by either non-biological [1] or biological approaches [2,3,4]. Rice consists of 69–72% endosperm, 20–21% rice hulls, and 8–11% rice bran (RB) [5,6]. It has been estimated that more than 63 million tons of RB are produced annually worldwide [7]. This can be compared to the recorded (2019/2020) and estimated (2021/2022) crop years, with global rice production of 499 and 505 million tons [8,9]. RB is the by-product of the rice industry after the rice milling process in which several components, such as germ, pericarp, seed coat, nucellus, and aleurone layers, have been removed [10,11] during subsequent milling and whitening stages. It is generally supplemented to animal feed to ensure sufficient presence of phosphorus, which can promote animal growth. In fact, nutritionally superior protein can also be extracted from RB, which is gluten-free with anti-cancer activities [12]. Phytic acid (IP6) or phytate (myo-inositol hexakisphosphate), an anti-nutrient carbohydrate, is particularly known as a major constituent of the organic phosphates and, hence, phosphate ions (Pi) in RB [13]. RB consists of IP6 within the range of 59.4–60.9 mg/g and can be utilized as a potential resource of nutrients and elements [10]. This compound is also widely distributed in many cereals, such as maize (7.2–22.2 mg/g), wheat (3.9–13.5 mg/g), barley (3.8–11.6 mg/g), sorghum (5.7–33.5 mg/g), oat (4.2–11.6 mg/g), and rice (0.6–10.8 mg/g) [14]. IP6 in RB is the main storage form of phosphorus that exerts preventive effects of mineral assimilation by the animals. The absorption inhibition of some essential trace elements and minerals (Ca^+^, Cu^2+^, Mg^2+^, Fe^2+^, Mn^2+^, and Zn^2+^) in the gut of the animals by IP6 is also evident [14,15]. Phytase enzyme (IP6 phosphohydrolase; EC 3.1.3.8 and EC 3.1.3.29, PE) is required to catalyze the release of phosphorus in the form of Pi from IP6 and can be found in a variety of microorganisms, including yeasts, fungi, and bacteria [16]. Eutrophication of water sources is one of the environmental concerns when Pi from undigested IP6 in animal waste is released uncontrollably through microbial digestion [17]. Previous studies suggested the application of concentrated Pi as a buffer to activate a certain enzyme, but an efficient recycling strategy was also necessary to prevent environmental impact [18]. The production of PE by yeasts, filamentous fungi, and bacteria were reported in *Aspergillus ficcum, Bacillus amyloliquefacien, B. subtilis, Candida torulopsis, C. tropicalis*, *Escherichia coli, Debaryomyces castelii*, *Klebsiella sp., Kluyveromyces fragilis, Lactobacillus* spp., *Penicillium oxalicum*, *Rhizopus microsporus*, *R. oryzae, Saccharomyces cerevisiae*, *Schizophyllum commune*, and *Schwanniomyces castellii* [15,19,20,21,22]. The conventional producer of PE was fungi, such as *Aspergillus* sp. [15]. In a recent investigation, the production of hyperactive PE derived from the thermotolerant *A. fumigatus* was demonstrated, specifically for the degradation of phytic acid in wheat flour [23]. Nevertheless, fungal phytases are frequently associated with a notable presence of undesirable proteases. This, in turn, requires extra purification or inhibition of proteolysis, leading to an escalation in production costs [24]. Furthermore, the exploration of fungal phytases has been prompted by the limitations encountered, such as substrate specificity, diminished thermostability, reduced resistance to proteolysis and acidity, along with catalytic inefficiency [25]. In contrast, bacterial PE exhibits diverse characteristics, including resistance to proteolysis, broad substrate specificity, high catalytic efficiency, and exceptional heat stability. Within bacterial strains, Lactobacillus is recognized as a prominent producer of phytase [26]. The PE activity (PE_act_) of lactic acid bacteria (LAB) strains isolated from different sources, including sourdough and fermented batter, has been demonstrated in previous studies [27,28]. The functional properties of RB could also be improved by co-culture fermentation with fungi and LAB for the production of potential food agents with medical applications in reducing blood pressure and maintaining glucose (Glu) homeostasis [22].

This study aimed to investigate the production of Pi, lactic acid (LA), other related organic compounds, as well as viable cell density (via-CD) by five LAB strains, which were isolated previously from traditional fermented food, in three types of IP6-containing media. The cultivation of LAB strains in the readily available IP6 medium as control were compared with those media containing milling stage rice bran (MsRB) and whitening stage rice bran (WsRB) during 72 h periods in a 5 × 3 factorial design experiment. Thirteen kinetic data and parameters, including mass yield percentage of LA, specific Glu consumption rate, specific product (LA and Pi) formation rates, intracellular and extracellular volumetric, as well as specific PE_act_, were also assessed, with scores ranked and compared statistically. The findings of this study will have the potential to be applied in the subsequent design and optimization of scale-up processes relevant to production.

## 2. Materials and Methods

### 2.1. Microorganisms

Thailand Institute of Scientific and Technological Research (TISTR) provided five lactic acid bacteria (LAB) strains previously isolated from traditional fermented food. The original nomenclature for genera of *Lactobacillus* spp. had been replaced with the new taxonomic groupings [29,30]. These were *Lacticaseibacillus casei* TISTR 1500, *Lactiplantibacillus plantarum* TISTR 877, *Latilactobacillus sakei* TISTR 890, *Limosilactobacillus fermentum* TISTR 055, and *Weissella confusa* TISTR 1498. The primary stock culture of each strain was prepared from the freeze-dried stock according to the recommended instruction by TISTR. Each stock was maintained in 80% (*v*/*v*) glycerol solution at −20 °C [2]. 

### 2.2. Rice Bran (RB)

Two types of jasmine RB, namely milling stage rice bran (MsRB) and whitening stage rice bran (WsRB), were purchased from Chiang Mai Polsuriya Milling Co Ltd., Sanpatong District, Chiang Mai Province, Thailand. MsRB was obtained separately from rice hulls after a milling process, while WsRB was the subsequent removal of the outer layer, including aleurone layer cells, from the rice kernel during the rice whitening process. The particle sizes of MsRB and WsRB were 603 ± 12 and 347 ± 6 µm, respectively. These were determined from a system of stacked woven wire sieve trays on a sieve shaker (Endecotts, Model No. Octagon 200, London, United Kingdom) and subsequent particle size distribution analysis [31].

### 2.3. Cultivation Media and Inoculum Preparation

#### 2.3.1. Cultivation Media

Lactobacillus-modified de Man, Rogosa and Sharpe (MRS) broth (HiMedia Laboratories) was used for inoculum propagation of each LAB strain. Dissolving 55.15 g of MRS broth powder in 1 L distilled water resulted in the following composition (g/L): proteose peptone, 10; beef extract, 10; yeast extract, 5; glucose (Glu), 20; Tween 80, 1; ammonium citrate (C_6_H_11_NO_7_), 2; sodium acetate (C_2_H_3_NaO_2_), 5; magnesium sulphate (MgSO₄), 0.1; manganese sulphate (MnSO₄), 0.05; and dipotassium hydrogen phosphate (K_2_HPO_4_), 2 (modified from HiMedia [32]. Phytate (IP6) medium was MRS broth with the addition of 5 g/L sodium phytate (C_6_H_6_O_24_P_6_Na_12_—Sigma Aldrich, IP6-Na) with pH adjustment to 6.00 using 10 M H₂SO₄ [28]. The theoretical maximum level of [Pi] that can be released from 5 g/L IP6-Na would thus be 3.08 g/L (Supplementary Section A4). RB media consisted of 200 mM ammonium sulphate ((NH₄)₂SO₄) [16] with the addition of 10% (*w*/*v*) MsRB or WsRB [33] prior to a similar pH adjustment procedure as IP6 medium. All media were sterilized at 121 °C and 15 psi for 15 min with a portable pressure sterilizer (All American, Model No.1925x, Wisconsin, USA) before microbial cultivation [4]. Pretreatment of both MsRB and WsRB powders, as described by Saad et al. [13] and Wattanapanom et al. [4], were unnecessary based on the rationale given in Supplementary Section A1.

#### 2.3.2. Inoculum Preparation

The inoculum size for each LAB strain cultivation was 10% (*v*/*v*). Seed inoculum for batch cultivation at 100 mL scale was prepared by transferring 1 mL of frozen culture stock that had been thawed at room temperature for 1 h to a sterilized McCartney bottle containing 9 mL MRS broth with a total inoculum volume of 10 mL. The bottle was then placed in an orbital shaker incubator (Daihan Labtech, Model No. LSI-3016R, Korea) at 37.0 ± 1.0 °C with 150 rpm shaking speed until the exponential growth phase of the inoculum was reached [16]. The viable cells densities (via-CDs) after inoculation of seed inocula for all five LAB strains were between 7.24–7.39 Log (CFU/mL) with an average of 7.33 ± 0.03 Log (CFU/mL).

### 2.4. Experimental Design for LAB Strains Cultivation

The 5 × 3 factorial design experiment with three replications was carried out for five LAB strains cultivation in three types of media, namely IP6, MsRB, and WsRB media, as mentioned in Section 2.3.1. Each microbial cultivation was carried out in a 250 mL non-baffled Erlenmeyer flask with 100 mL liquid working volume. The seed inocula of all five LAB strains were prepared as previously described and propagated in 100 mL IP6 medium with 10% (*v*/*v*) inoculum size. The microbial cultivation was performed at 37.0 ± 1.0 °C with 150 rpm shaking speed for 72 h. The pH levels were monitored throughout the cultivation time course in the absence of a pH control. The samples were collected in triplicates of 4 mL aliquots at cultivation periods of 0, 24, 48, and 72 h, respectively [21]. The specific details of sample treatment after collection, supernatant and cell pellet separation for subsequent analyses (organic compounds, total protein concentration ([Tprot]), and various PE_act_), as well as glass bead pretreatment prior to specific PE_act_ determination are provided in Supplementary Section A2.

### 2.5. Analytical Methods

The powder samples of MsRB and WsRB (200 g of each) were analyzed by the Central Laboratory (Thailand) using the Compendium of Methods for Food Analysis for total carbohydrate content [4]. The quantitative analyses for crude protein, crude fat, ash, and moisture contents in both samples were also performed by the Central Laboratory (Thailand) based on reference methods given by the Association of Official Analytical Chemists (AOAC), including 991.20, 948.15, (923.03 and 920.153), as well as (925.10 and 950.46), respectively [34]. The complete solubilization of IP6 in RB was carried out by following the modified methods of Ebrahimian and Motamedi [35], in which 10 g of sample was digested in 250 mL of 0.5 M HCl at 95 °C for 9 h. Determination of via-CD (Log(CFU/mL)) was also based on AOAC methods described by Maturin and Peeler [36]. The via-CD was used as a variable representing microbial growth instead of dried biomass concentration ([DB]) since the latter fluctuated widely throughout the cultivation time course due to the heterogeneity nature of RB cultivation media with carried-over insoluble solid affecting the precision and accuracy of [DB] determination.

A high-performance liquid chromatography (HPLC) was used for quantification of [Glu], [1,2-propanediol], [ethanol], [1-propanol], [butyric acid], [lactic acid] ([LA]), [formic acid], [acetic acid], [succinic acid], and solubilized [IP6] based on modified methods of Zaky et al. [37], Scherer et al. [38], da Costa et al. [39], Qamar et al. [40], and Marolt and Kolar [41]. Further details of specific HPLC conditioning (mobile phase, flow rate, and run time), type of detector, as well as retention time (RT) for each chemical species are elaborated in Supplementary Section A3. The mass yield percentage of produced [LA] over consumed [Glu] (Y_LA/Glu_) could then be calculated from HPLC results for IP6 medium during the 24–72 h cultivation time. Y_LA/Glu_ was not determined (n.d.) during LAB strains cultivation in MsRB and WsRB media due to the presence of relatively small [Glu], insignificant consumed [Glu], and/or relatively minute produced [LA].

The ammonium molybdate ((NH_4_)_6_Mo_7_O_24_· 4H_2_O) method was used to determine released [Pi] at 700 nm with a spectrophotometer (Cary 60 UV-Vis, Agilent Technologies, USA) from a standard curve with 0–10 mM K_2_HPO_4_ as standard solutions [42,43]. In the IP6 medium, [IP6] existed fully in soluble form, while [IP6] in both MsRB and WsRB media appeared in both soluble ([IP6]_sol_) and insoluble ([IP6]_in-sol_) forms. The overall [IP6] or [IP6]_overall_ for both media could then be determined from Equation (1). This was contrary to the IP6 medium, where all of [IP6] was readily available in the solubilized form ([IP6] = [IP6]_sol_). Supplementary Section A4 elucidates the mass balance calculation of produced [Pi] to the consumed [IP6] in each medium, as well as the determination of [IP6]_in-sol_ and [IP6]_overall_ based on produced [Pi].

[IP6]_overall_ = [IP6]_sol_ + [IP6]_in-sol_ (1)

One unit of PE_act_ (U) was defined as 1.0 μmol of liberated Pi per h at pH 5.5 and 50 °C and was determined based on the strategy provided by Haros et al. [44,45]. Volumetric PE_act_ (Vol-PE_act_ in U/mL) for both extracellular (ExVol-PE_act_) and intracellular (InVol-PE_act_) types were then obtained. The corresponding specific PE_act_ (Sp-PE_act_ in U/mg_protein_), namely ExSp-PE_act_ and InVol-PE_act_, were evaluated based on the ratio of respective Vol-PE_act_ and [Tprot] [2,3,46,47,48]. Further details are elucidated in Supplementary Section A5.

All chemicals used were of analytical reagent (AR) grade. At least three or five replicates of each sample were assessed to estimate the extent of random error for an individual measurement.

### 2.6. Determination of Kinetic Parameters

The important kinetic parameters in terms of specific rates, namely specific growth rate (μ) per h, specific Glu consumption rate (q_s,Glu_) in g Glu/L/Log(CFU/mL)/h, specific Pi production rate (q_p,Pi_) in g Pi/L/Log(CFU/mL)/h, and specific LA production (q_p,LA_) in g LA/L/Log(CFU/mL)/h, were computed from analyzed results (Section 2.5) between three periods of adjacent time intervals (0–24, 24–48, and 48–72 h) with modified strategies described previously by our group [2,4,48]. The specific IP6 consumption rate (q_s,IP6_) was not determined as q_p,Pi_ and could be easily converted to q_s,IP6_ after division by 0.6168 g Pi/g IP6 (IP6-Na equivalent), as described in Supplementary Section A4. The via-CD with the unit of Log(CFU/mL) was used instead of [DB] for the calculation of each specific rate, as also elucidated in Section 2.5.

### 2.7. Score Weighting Strategy

Thirteen items of kinetic data and parameters determined from each cultivation system were scored by normalization and combined with different weighting by following the modification of the previously published strategies [2,3,4,48] under specific conditioning for each item. These are (1) produced [Pi]; (2) mass balance of [IP6] on [Pi] production; (3) via-CD; (4) produced [LA]; (5) Y_LA/Glu_; (6, 7) produced ExVol-PE_act_ and ExSp-PE_act_; (8, 9) produced InVol-PE_act_ and InSp-PE_act_; (10) μ; (11) q_s,Glu_; (12) q_p,Pi_; and (13) q_p,LA_. The combination of these scores, resulting in the summation of kinetic data scores (D_Sc_) from individual subtotal D_Sc_ for each time point and summation of kinetic parameter scores (P_Sc_) whose subsequent combination either equivalent weighting (D_Sc_ + P_Sc_) or 2:1 weighting ratio (2D_Sc_ + P_Sc_), were used as selection criteria of the best LAB strain for each cultivation medium. Further details on specific conditioning and rationale for each type of score summation are given in Supplementary A6.

### 2.8. Hypothesis Testing

The identification and assessment of statistically significant differences between reported actual or score weighting results were carried out by the Duncan procedure using Statistical Product and Service Solutions (SPSS, version 17.0) for Microsoft Windows, with *p* ≤ 0.05 indicating statistical significance [2,4]. The computation of error propagation for each experimental result was described in previous work [3].

## 3. Results

### 3.1. Comparison of Nutritional Contents, IP6, and [Pi] in MsRB and WsRB

MsRB contained statistically significantly higher (*p* ≤ 0.05) total carbohydrate contents than WsRB by 1.36 ± 0.01 folds, as indicated in Table 1. This was in contrast to the availability of crude fat and protein contents in WsRB, which were statistically significantly (*p* ≤ 0.05) higher than those of MsRB by 7.70 ± 0.08 and 1.82 ± 0.04 times, respectively. Since full digestion of MsRB and WsRB resulted in 2.50 and 7.17 g IP6/100 g of RB (Table 1) and, hence, similar numbers for [IP6]_overall_ (g/L) of both media based on % (*w*/*v*) composition in Section 2.3.1, the highest possible [Pi] that could be available theoretically during LAB strains cultivation in both media would thus be 1.54 g/L and 4.42 g/L, respectively (Section 2.3.1 and Supplementary Section A4). The descending mass ratio of the highest possible released [Pi] in three media (WsRB:IP6:MsRB) was thus 4.42:3.08:1.54 or 2.87:2:1, respectively. Additional analyses of [IP6]_sol_ in all three media (data not shown in Table 1) revealed the following descending order (*p* ≤ 0.05) of available [IP6]_sol_ (g/L); IP6 (5.00 ± 0.02) > WsRB (3.91 ± 0.02) > MsRB (0.45 ± <0.01). The average concentration ratio of [IP6]_sol_ to the [IP6]_in-sol_ in both MsRB and WsRB media were thus 0.45 g/L: (2.50–0.45 = 2.05 g/L) or 0.45:2.05 = 0.22:1 and 3.91 g/L: (7.17–3.91 = 3.26 g/L) or 3.91:3.26 = 1.20:1, respectively (see Equation (1) Section 2.3.1).

### 3.2. Kinetic Data and Parameer Profiles of LAB Strains in Three Media

This section delineates the results of five LAB strains cultivation in IP6, MsRB, and WsRB media. The full species name of each LAB strain, as given in Section 2.1 and Supplementary Section B, will only be provided with the TISTR number for brevity, except at the end of this subsection where specific LAB strain(s) was/were selected for each medium. All plotted results in each figure of either this section or Supplementary Section B are also tabulated in Supplementary Section B under the directory given by Table S0.

**Figure 1:**
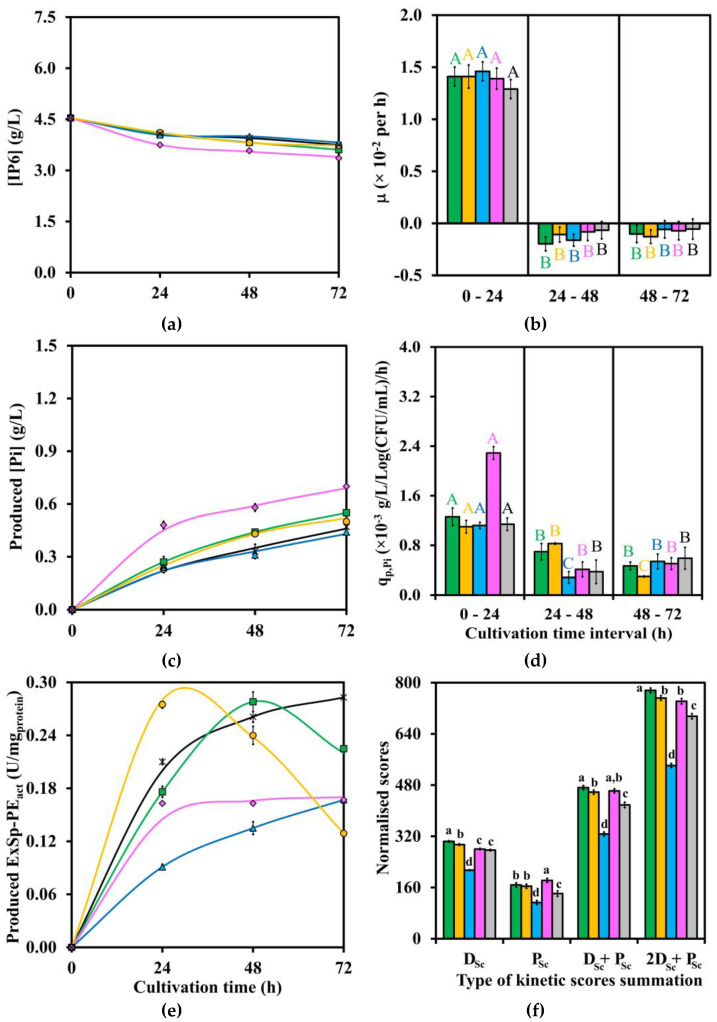
Kinetic data and parameter profiles as well as normalized weighting scores of five LAB strains (TISTR ■ 1500; ● 877; ▲ 890; ♦ 055; × 1498) during 72 h cultivation time in IP6 medium with respect to (**a**) [IP6]; (**b**) μ; (**c**) produced [Pi]; (**d**) q_p,Pi_; (**e**) produced ExSp-PE_act_; and (**f**) normalized scores. Each SE was included as an error bar for each data point. All [IP6] existed in [IP6]_sol_ form. The tabulated average and error results values for each LAB strain cultivation with a statistically significant comparison between time points of (**a**) are in Supplementary Section B Table S1.1; (**b**) in Table S1.6; (**c**) in Table S1.1; (**d**) in Table S1.6; (**e**) in Table S1.3; and (**f**) in Table S1.6. The statistically significant comparisons in (**b**,**d**) were made across three cultivation time intervals for each LAB strain with similar font coloring. The statistically significant comparison in (**f**) was made within each type of summation; D_Sc_; P_Sc_; D_Sc_ + P_Sc_; 2D_Sc_ + P_Sc_. The numbers with the same alphabet (A–C; a–d) indicate no statistically significant difference (*p* > 0.05).

**Figure 2:**
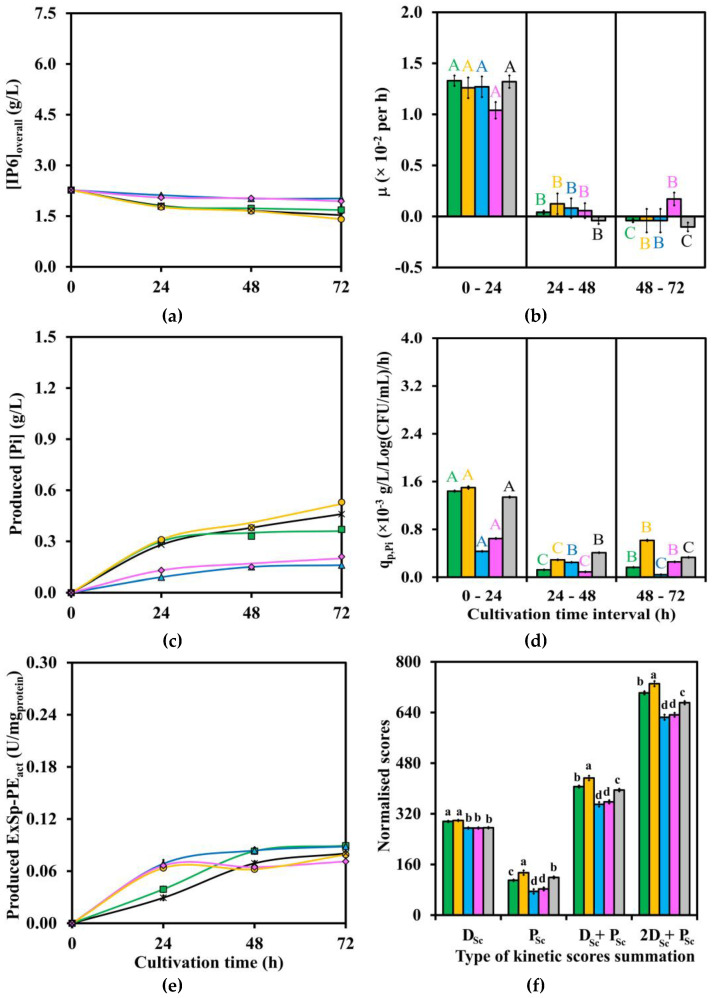
Kinetic data and parameter profiles as well as normalized weighting scores of five LAB strains (TISTR ■ 1500; ● 877; ▲ 890; ▲ 890; ♦ 055; × 1498) during 72 h cultivation time in MsRB medium with respect to (**a**) [IP6]_overall_; (**b**) μ; (**c**) produced [Pi]; (**d**) q_p,Pi_; (**e**) produced ExSp-PE_act_; and (**f**) normalized scores. Each SE was included as an error bar for each data point. [IP6]_overall_ existed in both [IP6]_sol_ and [IP6]_in-sol_ forms. The tabulated average and error results values for each LAB strain cultivation with a statistically significant comparison between time points of (**a**) are in Supplementary Section B Table S2.1; (**b**) in Table S2.6; (**c**) in Table S2.1; (**d**) in Table S2.6; (**e**) in Table S2.3; and (**f**) in Table S2.6. The statistically significant comparisons in (**b**,**d**) were made across three cultivation time intervals for each LAB strain with similar font coloring. The statistically significant comparison in (**f**) was made within each type of summation; D_Sc_; P_Sc_; D_Sc_ + P_Sc_; 2D_Sc_ + P_Sc_. The numbers with the same alphabet (A–C; a–d) indicate no statistically significant difference (*p* > 0.05).

**Figure 3:**
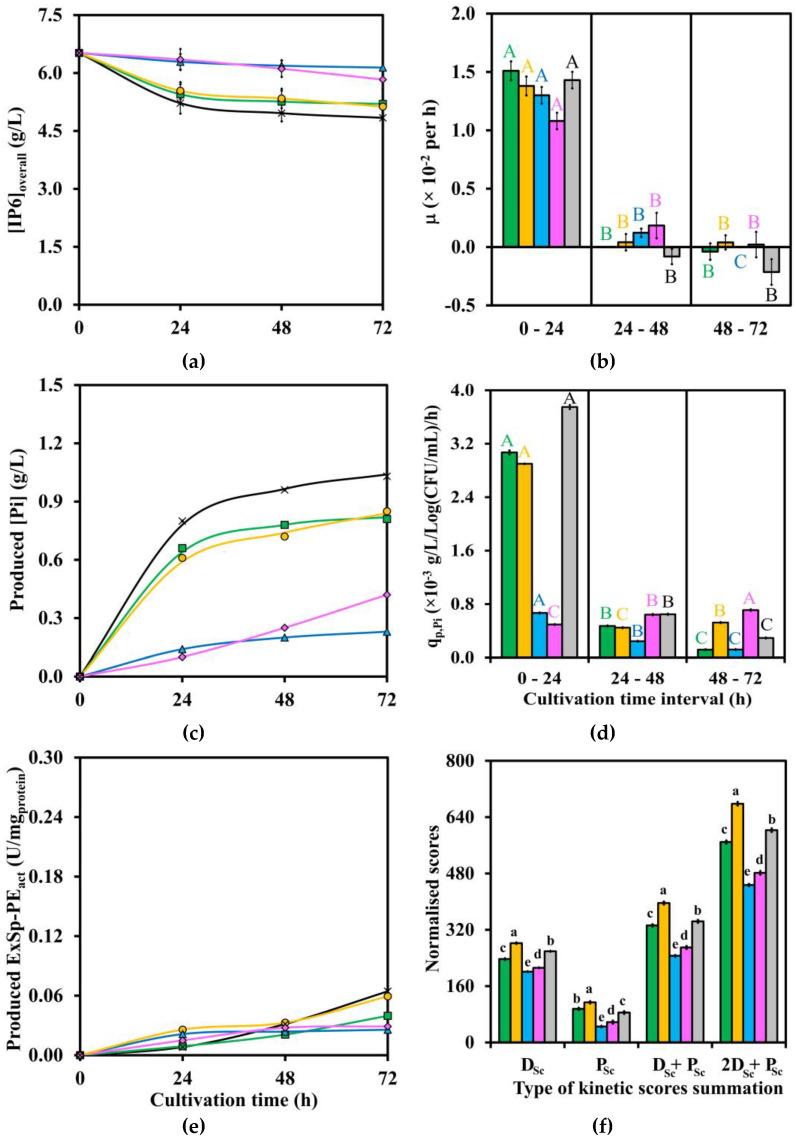
Kinetic data and parameter profiles as well as normalized weighting scores of five LAB strains (TISTR ■ 1500; ● 877; ▲ 890; ♦ 055; × 1498) during 72 h cultivation time in WsRB medium with respect to (**a**) [IP6]_overall_; (**b**) μ; (**c**) produced [Pi]; (**d**) q_p,Pi_; (e) produced ExSp-PE_act_; and (**f**) normalised scores. Each SE was included as an error bar for each data point. [IP6]_overall_ existed in both [IP6]_sol_ and [IP6]_in-sol_ forms. The tabulated average and error results values for each LAB strain cultivation with statistically significant comparison between time points of (**a**) are in Supplementary Section B Table S3.1; (**b**) in Table S3.6; (**c**) in Table S3.1; (**d**) in Table S3.6; (**e**) in Table S3.3; and (**f**) in Table S3.6. The statistically significant comparisons in (**b**,**d**) were made across three cultivation time intervals for each LAB strain with similar font coloring. The statistically significant comparison in (**f**) was made within each type of summation; D_Sc_; P_Sc_; D_Sc_ + P_Sc_; 2D_Sc_ + P_Sc_. The numbers with the same alphabet (A–C; a–d) indicate no statistically significant difference (*p* > 0.05).

Three crucial results of kinetic data profiles after LAB strains cultivation in IP6, MsRB, and WsRB media, including [IP6] or [IP6]_overall_, produced [Pi], and produced ExSp-PE_act_ at four time points of 0, 24, 48, and 72 h, are shown in Figure 1a, Figure 2a, Figure 3a, Figure 1c, Figure 2c, Figure 3c, and Figure 1e, Figure 2e, Figure 3e, respectively. In addition, the corresponding detailed kinetic data profiles of [Glu], produced [LA], and via-CD are given in Supplementary Section B under Figures S1a, S2a, S3a, S1c, S2c, S3c, and S1e, S2e, S3e, respectively. The additional results of mass balance percentage of [IP6] or [IP6]_overall_ on [Pi] production, produced [formic acid], produced [acetic acid], produced [ethanol], pH level, Y_LA/Glu_, produced ExVol-PE_act_, produced InVol-PE_act_, and produced InSp-PE_act_ are all numerically tabulated in Supplementary Section B. In addition, the results of [Ex-Tprot] and [In-Tprot] are not shown as they have already been incorporated into produced ExVol-PE_act_ and produced InVol-PE_act_, resulting in produced ExSp-PE_act_ and produced InSp-PE_act_.

Both two important kinetic parameter profiles, namely μ and q_p,Pi,_ are plotted and statistically significantly compared (*p* ≤ 0.05) during three time intervals of 0–24, 24–48, and 48–72 h, as shown in Figure 1b, Figure 2b, Figure 3b and Figure 1d, Figure 2d, Figure 3d for (IP6, MsRB, and WsRB) media, respectively. Four summation scores (D_Sc_, P_Sc_, D_Sc_ + P_Sc_, and 2D_Sc_ + P_Sc_) for all three media are also shown in Figure 1f, Figure 2f and Figure 3f. The additional plots of q_s,Glu_, q_p,LA,_ and normalized kinetic data scores are provided in Supplementary Section B under Figures S1b, S2b, S3b, S1d, S2d, S3d, and S1f, S2f, S3f, respectively.

#### 3.2.1. Comparison of Via-CD and Produced PE_act_

Figures S1e, S2e, and S3e, as well as Supplementary Tables S1.1–S3.1, indicate similar initial via-CD of all LAB strains in three media from 7.24–7.39 Log (CFU/mL). LAB strains (TISTR 1500, 877, and 890) cultivation in IP6 media followed a general trend of reaching the highest statistically significant (*p* ≤ 0.05) range of 10.2–10.5 Log (CFU/mL) at 24 h before dropping to the lower statistically significant (*p* ≤ 0.05) range of 9.64–9.96 Log (CFU/mL) at 72 h. This was in contrast with TISTR 055 and TISTR 1498, whose via-CD were maintained at the highest values for extended periods of time after 24 h for another 24 and 48 h, respectively. The delay of reaching the highest statistically significant (*p* ≤ 0.05) range of 9.95–10.2 Log (CFU/mL) was observed when TISTR 1500, 877, and 055 were cultivated in MsRB medium at 48, 48, and 72 h, respectively. A similar growth pattern as TISTR 877 in MsRB medium was elucidated for TISTR 890 and 055 in WsRB medium. In fact, TISTR 890 was the only LAB strain whose via-CD had plateaued out after reaching the highest statistically significant (*p* ≤ 0.05) at 24 h in MsRB medium. Such a trend of growth was detected again in TISTR 1500 and 877 in WsRB medium.

The ranges of the highest statistically significant (*p* ≤ 0.05) produced ExSp-PE_act_ in IP6, MsRB, and WsRB media were 0.163–0.283, 0.0664–0.0893, and 0.0238–0.0644 U/mg_protein_, respectively, as shown in Figure 1e, Figure 2e and Figure 3e. The ExSp-PE_act_ production pattern with the highest statistically significant (*p* ≤ 0.05) peaked at 24 and 48 h, followed by a subsequent drop in ExSp-PE_ac,_ which was only observed for TISTR 877 and 1500 in IP6 medium. Another production pattern of gradual ExSp-PE_act_ accumulation throughout the cultivation period, reaching the highest statistically significant (*p* ≤ 0.05) level of 72 h, was noticed for the cultivation of TISTR 890 and 1498 in IP6 medium, TISTR 877 and 1498 in MsRB medium as well as TISTR 1500, 877, and 1498 in WsRB medium. These were compared with produced InSp-PE_act_ (Supplementary Section B Tables S1.3–S3.3), of which the highest statistically significant (*p* ≤ 0.05) values (U/mg_protein_) were in the ranges of 0.222–1.090 for IP6 medium, 0.513–1.075 for MsRB medium, and 0.182–0.225 for WsRB medium. The produced InSp-PE_act_ reached the highest levels during 24–48 h after LAB cultivation in both IP6 and MsRB media. On the contrary, all LAB strains achieved the highest statistically significant (*p* ≤ 0.05) values of produced InSp-PE_act_ after 72 h cultivation in WsRB medium.

Similar patterns of produced ExVol-PE_act_ (Supplementary Section B Tables S1.3, S2.3, and S3.3) to those of produced ExSp-PE_act_ were generally observed for all LAB strains cultivation in three media, with a few exceptions of TISTR 1500 and 055 in MsRB medium, as well as TISTR 890 in WsRB medium. The highest statistically significant (*p* ≤ 0.05) ExVol-PE_act_ value was shifted to 72 h for the former group, while the latter strain reached the highest value earlier at 24 h. The ranges of the highest statistically significant (*p* ≤ 0.05) produced ExVol-PE_act_ in IP6, MsRB, and WsRB media were 0.0604–0.0927, 0.0224–0.0415, and 0.0248–0.0753 U/mL, respectively. The produced InVol-PE_act_ in IP6, MsRB, and WsRB media also generally followed the same trends as InSp-PE_act_, with corresponding ranges of the highest statistically significant (*p* ≤ 0.05) volumetric PE_act_ (U/mL) of 0.0202–0.0290, 0.0203–0.0236, and 0.0238–0.0321, respectively. Some delayed times of attaining the highest InVol-PE_act_ values compared to InSp-PE_act_ were detected in some LAB strains, namely TISTR 877 and 1498 in IP6 medium with 24 h delayed time, as well as TISTR 877, 890, and 1498 in MsRB medium with 24–48 h delayed time.

#### 3.2.2. Comparison of [Glu] and Related Produced By-Products

Figures S1a–S3a, as well as Supplementary Section B Tables S1.1–S3.1, indicate that LAB strains TISTR 877, 1500, and 1498 in IP6 medium could not consume all of the [Glu] present initially (18.0 g/L), with remnant [Glu] of 4.30–5.00 g/L at 72 h. This was compared to TISTR 055 and 890, whose [Glu] were completely consumed after 48 h. The [Glu] of TISTR 1498 plateaued at 48 h, with [Glu] of 4.30–5.10 g/L during 48–72 h. The initial [Glu] for MsRB and WsRB media were all less than 0.6 g/L with corresponding ranges of 0.37–0.58 g/L and 0.21–0.54 g/L, respectively. In addition, [Glu] was maintained at relatively constant levels throughout the cultivation period for TISTR 1500, 877, and 1498 in MsRB medium and TISTR 1500 in WsRB medium. In fact, TISTR 1500 was the only LAB strain whose remnant [Glu] did not drop to the level less than 0.10 g/L in both media, except at 72 h in WsRB medium (0.09 g/L).

The production of [LA], related produced by-products, and pH levels for all LAB strains in three media are illustrated and tabulated in Supplementary Section B Figures S1c–S3c, as well as Tables S1.2–S3.2. Production of butyric acid, succinic acid, 1-propanol, and 1,2-propanediol were not detected in all conditions. The produced [LA] for TISTR 1500 and 877 in IP6 medium was within the range of 11.1–11.5 g/L, while only 2.20–3.23 g/L was observed in WsRB medium. All LAB strains produced less than 0.5 g/L of [LA] in MsRB medium. Produced [formic acid] was not detected when all LAB strains were cultivated in MsRB medium. In fact, less than 1 g/L and 2 g/L of produced [formic acid] were observed for LAB cultivation in IP6 and WsRB media. In addition, TISTR 1498 could produce 5.38 ± 0.49 g/L [acetic acid] after 72 h cultivation in IP6 medium, while less than 3 g/L of [acetic acid] was produced during LAB strains cultivation in MsRB and WsRB media. The ability of TISTR 055 to produce 3.64 ± 0.04 g/L [ethanol] after 72 h cultivation in IP6 medium was recorded. This was compared to the mitigation of produced [ethanol] for LAB strains cultivation in both MsRB (<2 g/L) and WsRB (<0.7 g/L) media. The shift of the initial pH level range was observed with the following ascending order for IP6 medium (5.58–5.69), MsRB medium (5.62–5.77), and WsRB medium (5.73–5.99). The subsequent drops in the pH level range after 72 h cultivation were seen in all three media, with TISTR 1500 and 877 having the lowest pH levels as follows: 3.89–3.92 in IP6 medium, 4.31 in MsRB medium, and 4.16–4.29 in WsRB medium. These were compared with the relatively higher pH level ranges of TISTR 890, 055, and 1498 of 4.30–5.03 in IP6 medium, 4.46–4.75 in MsRB medium, and 4.83–4.86 in WsRB medium.

The assessment of Y_LA/Glu_ in IP6 medium is shown in Supplementary Section B Table S1.3, while Y_LA/Glu_ in both MsRB and WsRB media could not be determined (Tables S2.3 and S3.3) due to the presence of relatively low [Glu] (<0.6 g/L) as well as insignificant consumed [Glu] or produced [LA]. The maximum statistically significant Y_LA/Glu_ (g LA_produced_/g Glu_consumed_) (*p* ≤ 0.05) in IP6 medium were 0.84–0.89 (24–72 h for TISTR 1500), 0.98 ± 0.09 (48 h, TISTR 877), 0.60 ± 0.05 (24 h, TISTR 890), 0.75 ± 0.11 (24 h, TISTR 055), and 0.48–0.51 (24 and 72 h, TISTR 1498).

#### 3.2.3. Comparison of [IP6] and Produced [Pi]

The initial [IP6] or [IP6]_overall_ varied with each type of medium, as indicated in Figure 1a, Figure 2a and Figure 3a as well as Supplementary Section B Tables S1.1–S3.1, with corresponding concentration (g/L) before and after the addition of 10%(*v*/*v*) inoculum size as follows: [IP6] of (5.00 ± 0.02, 4.54 ± 0.02) for IP6 medium and [IP6]_overall_ of (2.50 ± 0.05, 2.27 v 0.04), as well as (7.17 ± 0.17, 6.52 ± 0.15) for MsRB and WsRB media. Generally, the decreasing trends of [IP6] or [IP6]_overall_ mirrored the increasing trends of produced [Pi] during LAB strains cultivation throughout the time course for all media, as shown in Figure 1c, Figure 2c and Figure 3c as well as Supplementary Section B Tables S1.1–S3.1 due to nearly 100% or 100% mass balance percentage of [IP6] on [Pi] production. The relatively rapid rate of produced [Pi] production during the first 24 h was followed by a slower rate or plateaued production until 72 h for each LAB strain. The LAB strains cultivation could reach produced [Pi] (g/L) of 0.70 ± 0.01 by TISTR 055 in IP6 medium, 0.53 ± <0.01 by TISTR 877 in MsRB medium, and 1.03 ± <0.01 by TISTR 1498 in WsRB medium.

#### 3.2.4. Comparison of Kinetic Parameters and Summation Scores

Both μ and q_p,Pi_, which were determined for three time intervals of 0–24, 24–48, and 48–72 h, are shown in Figure 1b,d for IP6 medium, 2b,d for MsRB medium, and 3b,d for WsRB medium, as well as tabulated numerically in Supplementary Section B Tables S1.6, S2.6, and S3.6. The positive μ values within the range of 1.29–1.46 × 10^−2^ per h during the first 24 h for LAB strains cultivation in IP6 medium were followed by statistically significant lower (*p* ≤ 0.05) negative values during subsequent time intervals. In fact, similar ranges of μ values at 1.26–1.33 × 10^−2^ per h for MsRB medium and 1.30–1.51 × 10^−2^ per h for WsRB medium during the first 24 h were observed, with the exception of TISTR 055, whose μ values were at lower ranges of 1.04–1.08 × 10^−2^ per h. The μ values during the subsequent time intervals were statistically significantly lower (*p* ≤ 0.05) with either slightly positive or negative values for both MsRB and WsRB media. These were compared to q_p,Pi_ values in which TISTR 055 could reach the value of 2.29 ± 0.10 (× 10^−3^ g/L/Log(CFU/mL)/h) during the first 24 h of cultivation in IP6 medium in relation to the other strains with q_p,Pi_ range of 1.10–1.26 (×10^−3^ g/L/Log(CFU/mL)/h). For MsRB medium, q_p,Pi_ (×10^−3^ g/L/Log(CFU/mL)/h) range of 1.34–1.50 was evident for TISTR 1500, 877, and 1498, while the lower range of 0.432–0.647 was observed for TISTR 890 and 055 during the first 24 h cultivation. The q_p,Pi_ value as high as 3.75 ± 0.03 (×10^−3^ g/L/Log(CFU/mL)/h) could be observed for TISTR 1498 cultivation in WsRB medium during the first 24 h. This was compared to the lower q_p,Pi_ (×10^−3^ g/L/Log(CFU/mL)/h) ranges of 2.90 (TISTR 877)–3.07 (TISTR 1500) as well as 0.494 (TISTR 055)–0.667 (TISTR 890). The q_p,Pi_ values during the subsequent time intervals in all media were generally statistically significant lower (*p* ≤ 0.05) than the first 24 h counterpart, with the exception of TISTR 055 during 48–72 h cultivation in WsRB medium whose q_p,Pi_ value was statistically significant higher (*p* ≤ 0.05).

The q_s,Glu_ and q_p,LA_ values were plotted and tabulated numerically in Supplementary Section B Figure S1b,d for IP6 medium, S2b,d for MsRB medium, and S3b,d for WsRB medium, as well as the same tables as μ and q_p,Pi_. The cultivation of TISTR 055 in IP6 medium during 24–48 h achieved the lowest q_s,Glu_ (× 10^−2^ g/L/Log(CFU/mL)/h) or the fastest rate of [Glu] consumption of -6.91 ± 0.08. This was compared to the range of q_p,LA_ between (2.73–3.06) × 10^−2^ g/L/Log(CFU/mL)/h) for TISTR 877 and 055 in the same medium and cultivation time interval. The insignificant q_s,Glu_ values were observed in MsRB and WsRB media due to the minute [Glu] in both media, while the relatively lower q_p,LA_ values in comparison to IP6 medium were also evident.

Table 2 summarizes the best D_Sc_ + P_Sc_ and 2D_Sc_ + P_Sc_ score values with corresponding TISTR number of LAB strain(s) for each medium, as compared in Figure 1f, Figure 2f and Figure 3f, as well as numerical values provided in Supplementary Section B Tables S1.5, S1.7, S2.5, S2.7, S3.5, and S3.7. The optimal subtotal D_Sc_ score values with the corresponding optimal cultivation time of these LAB strains in each medium are also summarized in Table 2, with individual numerical values given in Supplementary Section B Tables S1.5, S2.5, and S3.5. *L. plantarum* TISTR 877 was the optimal strain for cultivation in MsRB and WsRB media with a cultivation period of 72 h, while either *L. casei* TISTR 1500 or *L. fermentum* TISTR 055 could be the suitable LAB strains for cultivation in IP6 medium with cultivation periods of both 48 and 72 h, as their D_Sc_ + P_Sc_ and subtotal D_Sc_ score values were not statistically significantly different (*p* > 0.05). In fact, *L. casei* TISTR 1500 could be the only optimal LAB strain for 72 h cultivation in IP6 medium in the situation where 2D_Sc_ + P_Sc_ is used as the selection criterion.

## 4. Discussion

### 4.1. Comparison of Nutritional Contents and IP6 in MsRB and WsRB Powders

Bodie et al. [5] and Mohammadi et al. [49] reported the nutritional composition of RB in terms of mass percentages, indicating that it typically contains 34–52% carbohydrates, 15–22% lipids, 10–16% protein, 6–10% ash, and 8–12% moisture. These findings align with the results of the present study. The study observed that the total carbohydrate content in MsRB was significantly higher (*p* ≤ 0.05) than WsRB, potentially due to the elevated lignocellulosic content [6] from rice hulls. Singh [50] explained that rice hulls, constituting 20% (*w/w*) of the whole rice grain, consist of approximately 50% (*w/w*) cellulose, 25–30% (*w/w*) lignin, and 15–20% (*w/w*) silica. The MsRB, obtained earlier in the milling process, may include the inner bran layers rich in dietary fiber, contributing to the higher total carbohydrate content. Conversely, WsRB, obtained later in the process, might have undergone the removal of some inner bran layers, resulting in lower carbohydrate content. Moreover, variations in the degree of milling between MsRB and WsRB could contribute to differences in carbohydrate content [51]. The research findings of the present study align with the existing literature, and the observed differences in carbohydrate content between MsRB and WsRB are attributed to factors such as lignocellulosic content from rice hulls, the inclusion of inner bran layers, and variations in milling processes.

Furthermore, rice bran (RB) was found to be rich in various beneficial compounds, including vitamin E (0.32–0.44 mg/g), gamma-oryzanol (3.86–5.89 mg/g), and phenolic compounds (9.60–81.85 mg gallic acid equivalent/g), along with other bioactive compounds [49,52]. The presence of polyphenolic compounds was observed to have a less detrimental effect on the growth of LAB compared to other types of bacteria. In fact, it could even be beneficial due to synergistic effects and the tailored metabolic pathways of LAB that are compatible with polyphenols [53]. Saad et al. [13] highlighted that RB is generally abundant in IP6 relative to other parts of rice grain, with a typical composition of 1.5–6.4% (*w/w*) in whole grain cereals. Kortekangas et al. [54] reported an IP6 content of 5.0–8.7% (*w/w*) in RB, primarily located in aleurone layer cells. The aleurone layer, positioned at the outermost part of the rice endosperm, acts as a protective coat and contains IP6 strongly bound to minerals such as zinc, iron, and magnesium [55]. The IP6 composition of WsRB at 7.17% (*w/w*) in Table 1 aligned well with the previous report by Kortekangas et al. [54]. On the contrary, the relatively lower IP6 content of MsRB at 2.50% (*w/w*) suggests that the milling process in this study only partially removed the aleurone layer cells. Sim et al. [6] reported extraction yields of IP6 from rice hulls to be between 1.91 and 2.28% (*w/w*), consistent with the current study for MsRB. The presence of relatively lower [IP6]_sol_ in each type of RB medium (17.8 ± 0.4% (*w/w*) for MsRB and 54.5 ± 1.3% (*w/w*) for WsRB) compared to the total available [IP6] in RB was not surprising. This could be due to a significant portion of IP6 remaining in the insoluble forms ([IP6]_in-sol_), such as lignocellulosic materials/carbohydrates in rice hulls of MsRB [6,56] or various proteins in aleurone layer cells in WsRB [54], affecting their solubility. These [IP6]_in-sol_ might be gradually released and utilized throughout the cultivation time course of LAB strains due to microbial metabolisms. It is important to note that the [IP6]_sol_ of IP6 medium (5.00 ± 0.02 g/L), MsRB medium (0.45 ± <0.01 g/L), and WsRB medium (3.91 ± 0.02 g/L) at the beginning of LAB strains cultivation was not expected to cause any significant inhibitory effect on PE. PE could easily remove all of [IP6] from soymilk, even with a relatively high [IP6] of 5.6 g/L, to generate an IP6-free product [57].

### 4.2. Kinetic Data and Parameter Profiles of LAB Strains in Three Media

#### 4.2.1. Comparison of Via-CD and Produced PE_act_

The LAB strains investigated in this study were isolated from Thai local pickles and fermented meat products. Previous studies have shown variations in phytase activity among LAB strains originating from different sources. For instance, Karaman et al. [58] isolated 49 LAB and 53 yeast strains from Turkish sourdough, with LAB isolates displaying phytase activities ranging from 703–1154 U/mL and yeast isolates ranging from 352–943 U/mL. Traditionally, LAB phytases were known for their intracellular or cell-bound activities [20,59]; however, our study reveals a departure from this norm, indicating that the LAB isolates exhibit both extracellular and intracellular phytase activities. Intriguingly, most strains displayed higher extracellular activity than intracellular activity, a finding consistent with Nuobariene et al. [60], who observed both extracellular and intracellular phytase activity in four LAB strains, *Lactobacillus panis*, *L. reuteri*, *L. fermentum*, and *Pediococcus pentosaceus*, isolated from Lithuanian sourdough. The authors reported the presence of both extracellular and intracellular phytase activity in LAB strains, and our findings align with this observation. The majority of strains showed higher extracellular activity compared to their intracellular counterparts. This is consistent with the work of Mohammadi-Kouchesfahani et al. [61], who argued that LAB strains with significant extracellular PE_act_ would be more effective as starter cultures in the bakery industry. The presence of high IP6 levels in wholemeal bread can affect mineral bioavailability for consumers, and extracellular PE during sourdough production could efficiently dissociate IP6. The ExVol-PE_act_ (0.068 ± 0.003 U/mL) and ExSp-PE_act_ (5.38 ± 0.37 U/mg protein) of TISTR 1500 cultivated in MsRB medium were significantly higher (*p* ≤ 0.05) than those reported for two other strains. These results may find correlation with a prior study by Haros et al. [45], which investigated the IP6-degrading and phosphatase activities of several LAB strains isolated from different sources. The PEact of LAB depends on strains and substrates, as observed in various studies [45,62,63,64]. Consistent with these findings, our current investigation aligns, where LAB strain TISTR 877, characterized by the highest rate of glucose consumption, displayed augmented extracellular phytase activity. This association is indicative of an elevated specific growth rate, highlighting the interplay between substrate utilization, growth rate, and phytase activity in LAB strains.

#### 4.2.2. Comparison of [Glu] and Produced By-Products

It was expected that both TISTR 1500 and 877 would be relatively proficient producers of [LA], considering that the other three LAB strains tended to generate higher levels of [formic acid], [acetic acid], and [ethanol], which balanced out the overall production of [LA]. Typically, in addition to lactic acid, LAB have the capacity to convert pyruvate into acetic acid, formic acid, and ethanol through hetero-lactic fermentation [65]. This conversion is influenced by the presence of additional substrates that act as electron acceptors, primarily fructose, oxygen, malate, and citrate, through the 6-phosphogluconate/phosphoketolase (6-PG/PK) pathway. In the current study, all five LAB strains exhibited the utilization of the pentose phosphoketolase pathway for heterofermentative metabolism, producing LA, ethanol, and acetic acid over the cultivation period in IP6 medium [66]. LA and acetic acid were produced by all five LAB strains at varying levels, while ethanol production was observed only for TISTR 055 and TISTR 1498. Within the citrate metabolism pathway of LAB, citrate undergoes decarboxylation to form oxaloacetate, followed by a gradual reduction process resulting in the formation of malic and succinic acids. This reduction process requires two NADH molecules, and the generated NADH can be used as a cofactor to facilitate the conversion of lactate to pyruvate. This conversion enables the synthesis of ATP through the generation of acetyl phosphate, ultimately evolving into acetic acid [67]. Notably, TISTR 1500 and TISTR 877 demonstrated remarkable capabilities as LA producers, achieving significant LA production, as evidenced by their LA yield percentage relative to the mass of consumed glucose (Y_LA/Glu_). Specifically, TISTR 1500 yielded 11.1 ± 0.06 g/L at 72 h, with a yield ratio ranging from 0.84–0.89 g/g between 24 and 72 h. In comparison, TISTR 877 achieved an LA production of 11.5 ± 0.07 g/L at 72 h, with a yield ratio of 0.98 ± 0.09 g/g at 48 h. Similar yield ratio ranges have been reported in previous studies for various LAB strains with different substrates [66]. The observed relatively low pH mitigation in some LAB strains in this study may be correlated to the overall production of weak acids by each LAB strain, with LA (pK_a_ = 3.78) being a predominant acidic compound [66]. For instance, the decrease in pH noted after 72 h of cultivation with TISTR 1500 and 877 was attributed to the ongoing metabolic processes of LAB, leading to the generation of various mild organic acids. These acids likely contributed to the decline in pH, possibly reaching a critical threshold during fermentation, as observed in previous studies [63,68,69]. The combined production of acidic compounds for TISTR 1500 and TISTR 877 ranged from 11.6–13.4 g/L at 72 h (including LA and acetic acid). This can be compared with TISTR 055 and TISTR 1498, whose combined [LA], [formic acid], and [acetic acid] ranged from 8.99–12.8 g/L at 72 h. The initial [LA] of 3.44 ± 0.21 g/L, generated by *L. sakei* TISTR 890 within 24 h, was completely depleted by the 72 h mark. This depletion was accompanied by the production of formic and acetic acids, reaching a combined concentration of 4.85 ± 0.28 g/L. Abedi and Hashemi [66] also noted the inhibitory effect of relatively low pH (< 4) on cell growth and LA production. The acidic environment resulting from LAB activity creates a conducive atmosphere for the breakdown of IP6, facilitating the activation of endogenous phytase and enhancing phytate degradation in various food types such as sourdough, white sorghum, maize gruels, and cereal dough [57,70]. Similarly, Leenhardt et al. [71] observed in their study that slight acidification of the dough due to organic acid production was favorable for wheat phytase activity, effectively reducing the phytic acid content [71]. However, a rapid decline in pH can impact the efficacy of the phytase enzyme, as excessively low pH levels hinder its function. The optimal activity for the enzyme was observed in a pH range of approximately 4.5 to 5.5 [63].

#### 4.2.3. Comparison of [IP6] and Produced [Pi]

Karaman et al. [58] noted that the addition of LAB isolates to whole wheat bread significantly and statistically mitigated (***p*** ≤ 0.05) the IP6 content compared to the control. Interestingly, the use of co-cultures, such as *Saccharomyces cerevisiae* and *Pediococcus pentosaceus*, resulted in the most substantial decrease in [IP6] levels (43.4%). In another study by Fischer et al. [27], 76 LAB strains isolated from 13 different fermented tef-injera (Ethiopian soft pancake) were screened for their abilities to degrade IP6 on MRS agar medium supplemented with IP6. The tef-injera fermentation resulted in the lowest IP6 contents when *Lactobacillus buchneri* (41% IP6) and *P. pentosaceus* (42% IP6) were utilized. The observed trends observed in this study, where [IP6] decreased and produced [Pi] increased during the cultivation of LAB strains, might be attributed to the growth of LAB. This growth could lead to the depletion of phytate, ultimately resulting in the accumulation of inorganic phosphate. The phosphate accumulation arises from the release of phosphates as a consequence of phytate degradation, as discussed earlier [72,73]. The initial 24 h period showed a relatively swift pace of [Pi] production, which could potentially be attributed to the phenomenon described by Lopez et al. [73]. In their findings, the activity of the phytase enzyme was hindered by the accumulation of released phosphate, leading to a notable decline in phytate hydrolysis. They noted that the majority of phytate hydrolysis occurred within the initial 2 h of fermentation, characterized by a rapid release of phosphorus that subsequently decelerated [73].

## 5. Conclusions

To summarize, this study represents a pioneering effort to provide a comprehensive assessment of phosphate ion ([Pi]) production, lactic acid ([LA]) generation, other organic compound outputs, and diverse types of phytase activity (PE_act_) across five LAB strains. These strains, isolated from traditional fermented foods, underwent cultivation in various media, including a conventional IP6 medium as a control, as well as MsRB and WsRB media, which led to diverse outcomes in terms of [Pi] production, organic compound generation, and phytase activity. By meticulously analyzing thirteen pertinent kinetic data and parameters, encompassing maximum specific rates of growth, glucose consumption, [Pi] and [LA] formation, valuable insights have been gained. These findings not only aid in the selection of optimal LAB strains for specific media and durations but also lay the foundation for subsequent endeavors in scaling-up design and refining production processes. The findings shed light on the possible implementations of LAB and phytase in enhancing the nutritional value of foods by releasing [Pi] from phytate, improving mineral availability. This study stands as a significant contribution to the understanding of LAB behavior in different fermentation contexts, with potential applications in enhancing traditional fermented food production through optimized and scaled processes. While the current study has focused on establishing important connections between kinetic data and parameters associated with LAB cultivation in different media, we intend to investigate further the specific biochemical interactions between the components of the matrices and their influence on phytase activity and mineral release in future research endeavors.

## Figures and Tables

**Table 1:** Nutritional contents, IP6, and Pi of MsRB and WsRB.

Components	Nutritional Contents (g/100 g)	
	MsRB	WsRB
Total carbohydrate	**64.68^A^ ± 0.20**	47.65^B^ ± 0.16
Crude fat	2.55^B^ ± 0.02	**19.64^A^ ± 0.12**
Crude protein	7.90^B^ ± 0.17	**14.40^A^ ± 0.02**
Ash	**16.11^A^ ± 0.02**	9.97^B^ ± 0.03
Moisture	**8.76^A^ ± 0.01**	8.34^B^ ± 0.01
IP6 (fully digested) - Pi (% *w*/*w*) in IP6 - non-Pi in IP6 (% *w*/*w*)	2.50^B^ ± 0.05 1.54^B^ ± 0.03 (61.68%) 0.96^B^ ± 0.06 (38.32%)	**7.17^A^ ± 0.17** **4.42^A^ ± 0.10 (61.68%)** **2.75^A^ ± 0.20 (38.32%)**

Note: The numbers with the same alphabet (^A,B^) indicate no statistically significant differences (*p* > 0.05) for comparison between different columns of the same rows.

**Table 2:** Summary of the best score combination and subtotal D_Sc_ values for the selection of suitable LAB strain(s) and corresponding optimal cultivation time(s) in each type of cultivation medium.

Type of Medium	Best Score Combination and Subtotal D_Sc_ Values with Corresponding TISTR Number of LAB Strain and Cultivation Time						
	Type of Score Combination	Score Combination Value	TISTR	Cited Table	Subtotal D_Sc_ Value	Cultivation Time (h)	Cited Table
IP6	D_Sc_ + P_Sc_	472 ^ns1^ ± 6 462 ^ns1^ ± 6	1500 055	S1.7 S1.7	99.1^ns2^ ± 1.0 98.9 ^ns2^ ± 1.0 87.3 ^ns3^ ± 1.0 88.3 ^ns3^ ± 0.9	48 72 48 72	S1.5 S1.5
	2D_Sc_ + P_Sc_	776 ± 7	1500	S1.7	99.1 ± 1.0	48	S1.5
MsRB	D_Sc_ + P_Sc_	433 ± 7	877	S2.7	100.0 ± 1.4	72	S2.5
	2D_Sc_ + P_Sc_	731 ± 8	877	S2.7	100.0 ± 1.4	72	S2.5
WsRB	D_Sc_ + P_Sc_	396 ± 4	877	S3.7	100.0 ± 1.0	72	S3.5
	2D_Sc_ + P_Sc_	678 ± 5	877	S3.7	100.0 ± 1.0	72	S3.5

**Note**: The tabulated values for each type of score combination and subtotal D_Sc_ were the highest statistically significant (*p* ≤ 0.05) among their peers in each cited table from Supplementary Section B with respect to each type of medium as well as retrieved corresponding TISTR number and optimal cultivation time. Both TISTR 1500 and 055 were optimal LAB strains to be cultivated in IP6 medium based on D_Sc_ + P_Sc_ scores for either 48 or 72 h cultivation time, as corresponding score combination values and subtotal D_Sc_ values of these LAB strains were not different statistically (*p* ≤ 0.05). The symbols ^ns1, ns2^, and ^ns3^ denote non-significant differences for statistical comparison within the row or column of specified cited tables.

## Data Availability

The data presented in this study are available upon request from the corresponding authors.

## Supplementary Materials

The following supporting information can be downloaded at: https://www.mdpi.com/article/10.3390/biom13121770/s1, Table S0: Directory of LAB strains cultivation results using “Sx.y” format with respect to each y value; Table S1.1: Detailed kinetics data for five LAB strains cultivation in IP6 medium during 0–72 h with respect to [Glu], [IP6], produced [Pi], mass balance percentage of [IP6] on [Pi] production, and via-CD.; Table S1.2: Detailed kinetics data for five LAB strains cultivation in IP6 medium during 0–72 h with respect to produced [LA], produced [formic acid], produced [acetic acid], produced [ethanol], and pH level. Production of butyric acid, succinic acid, 1-propanol, 1,2-propanediol were not detected in all cases; Table S1.3: Detailed kinetics data for five LAB strains cultivation in IP6 medium during 0–72 h with respect to YLA/Glu, produced ExVol-PEact, produced ExSp-PEact, produced InVol-PEact, and produced InSp-PEact.; Table S1.4: Detailed kinetics data scores from Tables S1.1–S1.3 for five LAB strains cultivation in IP6 medium during 0–72 h with respect to produced [Pi], mass balance percentage of [IP6] on [Pi] production, via-CD, produced [LA], and YLA/Glu.; Table S1.5: Detailed kinetics data scores from Table S1.3 for five LAB strains cultivation in IP6 medium during 0–72 h with respect to produced ExVol-PEact, produced ExSp-PEact, produced InVol-PEact, and produced InSp-PEact as well as overall normalised scores from Tables S1.4 and S1.5; Table S1.6: Detailed kinetics parameters for five LAB strains cultivation in IP6 medium during three time intervals between 0–72 h with respect to m, qs,Glu, qp,Pi, and qp,LA.; Table S1.7: Detailed kinetics parameters scores from Table S1.6 for five LAB strains cultivation in IP6 medium during three time intervals between 0–72 h with respect to m, qs,Glu, qp,Pi, and qp,LA as well as overall normalised scores; Table S2.1: Detailed kinetics data for five LAB strains cultivation in MsRB medium during 0–72 h with respect to [Glu], [IP6], produced [Pi], mass balance percentage of [IP6] on [Pi] production, and via-CD.; Table S2.2: Detailed kinetics data for five LAB strains cultivation in MsRB medium during 0–72 h with respect to produced [LA], produced [formic acid], produced [acetic acid], produced [ethanol], and pH level. Production of butyric acid, succinic acid, 1-propanol, 1,2-propanediol were not detected in all cases; Table S2.3: Detailed kinetics data for five LAB strains cultivation in MsRB medium during 0–72 h with respect to YLA/Glu, produced ExVol-PEact, produced ExSp-PEact, produced InVol-PEact, and produced InSp-PEact.; Table S2.4: Detailed kinetics data scores from Tables S2.1–S2.3 for five LAB strains cultivation in MsRB medium during 0–72 h with respect to produced [Pi], mass balance percentage of [IP6] on produced [Pi], via-CD, produced [LA], and YLA/Glu.; Table S2.5: Detailed kinetics data scores from Table S2.3 for five LAB strains cultivation in MsRB medium during 0–72 h with respect to produced ExVol-PEact, produced ExSp-PEact, produced InVol-PEact, and produced InSp-PEact as well as overall normalised scores from Tables S2.4 and S2.5; Table S2.6: Detailed kinetics parameters for five LAB strains cultivation in MsRB medium during three time intervals between 0–72 h with respect to m, qs,Glu, qp,Pi, and qp,LA.; Table S2.7: Detailed kinetics parameters scores from Table S2.6 for five LAB strains cultivation in MsRB medium during three time intervals between 0–72 h with respect to m, qs,Glu, qp,Pi, and qp,LA as well as overall normalised scores; Table S3.1: Detailed kinetics data for five LAB strains cultivation in WsRB medium during 0–72 h with respect to [Glu], [IP6], produced [Pi], mass balance percentage of [IP6] on [Pi] production, and via-CD.; Table S3.2: Detailed kinetics data for five LAB strains cultivation in WsRB medium during 0–72 h with respect to produced [LA], produced [formic acid], produced [acetic acid], produced [ethanol], and pH level. Production of butyric acid, succinic acid, 1-propanol, 1,2-propanediol were not detected in all cases; Table S3.3: Detailed kinetics data for five LAB strains cultivation in WsRB medium during 0–72 h with respect to YLA/Glu, produced ExVol-PEact, produced ExSp-PEact, produced InVol-PEact, and produced InSp-PEact.; Table S3.4: Detailed kinetics data scores from Tables S3.1–S3.3 for five LAB strains cultivation in WsRB medium during 0–72 h with respect to produced [Pi], mass balance percentage of [IP6] on [Pi] production, via-CD, produced [LA], and YLA/Glu.; Table S3.5: Detailed kinetics data scores from Table S3.3 for five LAB strains cultivation in WsRB medium during 0–72 h with respect to produced ExVol-PEact, produced ExSp-PEact, produced InVol-PEact, and produced InSp-PEact as well as overall normalised scores from Tables S3.4 and S3.5; Table S3.6: Detailed kinetics parameters for five LAB strains cultivation in WsRB medium during three time intervals between 0–72 h with respect to m, qs,Glu, qp,Pi, and qp,LA.Table S3.7: Detailed kinetics parameters scores from Table S3.6 for five LAB strains cultivation in WsRB medium during three time intervals between 0–72 h with respect to m, qs,Glu, qp,Pi, and qp,LA as well as overall normalised scores; Figure S1: Kinetic data and parameters profiles as well as normalised weighting scores of five LAB strains (TISTR 1500; 877; 890; 055; 1498) during 72 h cultivation time in IP6 medium with respect to (a) [Glu]; (b) qs,Glu; (c) produced [LA]; (d) qp,LA; (e) via-CD; and (f) subtotal DSc. Each SE was included as an error bar to each data point. The tabulated average and error results values for each LAB strain cultivation with stastistical significant comparison between time points of (a) were in Supplementary Section B Table S1.1; (b) in Table S1.6; (c) in Table S1.2; (d) in Table S1.6; (e) in Table S1.1; and (f) in Table S1.5. The statistically significant comparison in (b), (d), and (f) were made across three cultivation time intervals or four cultivation time periods for each LAB strain with similar font coloring. The numbers with the same alphabet (A–D) indicated no statistically significant difference (*p* > 0.05).; Figure S2: Kinetic data and parameters profiles as well as normalised weighting scores of five LAB strains (TISTR 1500; 877; 890; 055; 1498) during 72 h cultivation time in MsRB medium with respect to (a) [Glu]; (b) qs,Glu; (c) produced [LA]; (d) qp,LA; (e) via-CD; and (f) subtotal DSc. Each SE was included as an error bar to each data point. The tabulated average and error results values for each LAB strain cultivation with stastistical significant comparison between time points of (a) were in Supplementary Section B Table S2.1; (b) in Table S2.6; (c) in Table S2.2; (d) in Table S2.6; (e) in Table S2.1; and (f) in Table S2.5. The statistically significant comparison in (b), (d), and (f) were made across three cultivation time intervals or four cultivation time periods for each LAB strain with similar font coloring. The numbers with the same alphabet (A–D) indicated no statistically significant difference (*p* > 0.05).; Figure S3: Kinetic data and parameters profiles as well as normalised weighting scores of five LAB strains (TISTR 1500; 877; 890; 055; 1498) during 72 h cultivation time in WsRB medium with respect to (a) [Glu]; (b) qs,Glu; (c) produced [LA]; (d) qp,LA; (e) via-CD; and (f) subtotal DSc. Each SE was included as an error bar to each data point. The tabulated average and error results values for each LAB strain cultivation with stastistical significant comparison between time points of (a) were in Supplementary Section B Table S3.1; (b) in Table S3.6; (c) in Table S3.2; (d) in Table S3.6; (e) in Table S3.1; and (f) in Table S3.5. The statistically significant comparison in (b), (d), and (f) were made across three cultivation time intervals or four cultivation time periods for each LAB strain with similar font coloring. The numbers with the same alphabet (A–D) indicated no statistically significant difference (*p* > 0.05).

## Abbreviations and Symbols

[ ]	concentration of chemical species being enclosed by square bracket
µ	specific growth rate
act	activity
AOAC	Association of Official Analytical Chemists
AR	analytical reagent
CFU	colony forming unit
DAD	diode array detector
DB	dried biomass
D_Sc_	kinetic data score
ExSp-PE_act_	extracellular specific PE_act_
Ex-Tprot	extracellular total protein
ExVol-PE_act_	extracellular volumetric PE_act_
g	gram
Glu	glucose
HPLC	high-performance liquid chromatography
InSp-PE_act_	intracellular specific PE_act_
In-Tprot	intracellular total protein
InVol-PE_act_	intracellular volumetric PE_act_
IP6	phytic acid/phytate
[IP6]_sol_	soluble form of IP6 in MsRB or WsRB media
[IP6]_in-sol_	insoluble form of IP6 in MsRB or WsRB media
[IP6]_overall_	overall [IP6] equal to summation of [IP6]_sol_ and [IP6]_in-sol_
IP6-Na	sodium phytate
LA	lactic acid
LAB	lactic acid bacteria
Log	common logarithm, decadic logarithm
m	milli
MRS	de Man, Rogosa, and Sharpe (Lactobacillus modified broth)
MsRB	milling stage RB
n.d.	not determined
PE	phytase enzyme
PE_act_	phytase enzyme activity
Pi	phosphate ions
P_Sc_	kinetic parameter score
q_p,LA_	specific LA production rate (g LA/L/Log(CFU/mL)/h)
q_p,Pi_	specific Pi production rate (g Pi/L/Log(CFU/mL)/h)
q_s,Glu_	specific Glu consumption rate (g Glu/L/Log(CFU/mL)/h)
q_s,IP6_	specific IP6 consumption rate (g IP6/L/Log(CFU/mL)/h)
RB	rice bran
RID	refractive index detector
RT	retention time
S	supplementary
SE	standard error
Sp-PE_act_	specific PE_act_
SPSS	Statistical Product and Service Solutions
TISTR	Thailand Institute of Scientific and Technological Research
Tprot	total protein
U	unit of PE_act_
USA	United States of America
UV-Vis	ultraviolet-visible
via-CD	viable cells density
Vol-PE_act_	volumetric PE_act_
WsRB	whitening stage RB
Y_LA/Glu_	mass yield percentage of produced [LA] over consumed [Glu]
