# Supplementary material for: Kinetics of Phosphate Ions and Phytase Activity Production for Lactic Acid-Producing Bacteria Utilizing Milling and Whitening Stages Rice Bran as Biopolymer Substrates
Source: Biomolecules. 2023 Dec 10;13(12):1770. doi: 10.3390/biom13121770 (PMC10741578; doi:10.3390/biom13121770)
Supplement: Supplementary file 1 [file biomolecules-13-01770-s001.zip › biomolecules-2659671-supplementary.pdf]

Supplementary Section A

A1: Pretreatment of both MsRB and WsRB powders were unnecessary either by acidic pretreatment with 5% (v/v) sulfuric acid [13] or enzymatic pretreatment by an enzyme mixture of amylase, glucoamylase, cellulase, and xylanase as described by Wattanapanom *et al.* [4]. Since the kinetic data and parameters obtained after LAB strains cultivation between untreated and pretreated MsRB and WsRB powders were not statistically significant different ($p > 0.05$) (data not shown), MsRB and WsRB media only utilized unpretreated form of respective RB powders.

A2: Ice-water mixture was used to cool down the samples immediately prior to storage at -20°C for further analyses. Via-CD for each replicate was determined from 1 mL aliquot of the well-mixed collected sample [16]. The remnant sample volume of each collected sample was then subjected to centrifugation by a centrifuge machine (Nuve, Model No. NF200, Turkey) at 2,822 × g for 5 min to separate supertanant from cells pellet [3, 4]. The items to be analyzed from separated supernatant included concentration level of organic compounds (Glu, IP6, phosphate (Pi), lactic acid (LA), formic acid, acetic acid, ethanol, butyric acid, succinic acid, 1-propanol, and 1,2 - propanediol), pH level, extracellular total protein concentration ([Ex-Tprot]), extracellular volumetric as well as specific PE activity (ExVol-PE_{act} and ExSp-PE_{act}) as stated in section 2.5. Collected cells pellet from each sample was attrited by glass bead strategy using modified method by Nunta *et al.* [2]. The liquid portion from glass bead - treated mixture was obtained by similar centrifugation condition described previously and subsequently analyzed for intracellular total protein concentration ([In-Tprot]), intracellular volumetric as well as specific PE activities (InVol-PE_{act} and InSp-PE_{act}) as described in section 2.5. The mass yield percentage of LA and relevant kinetic parameters were also assessed and evaluated (section 2.6). All data sets were score weighted and statistically compared with detailed strategies mentioned in section 2.7 and 2.8.

A3: The quantification of [Glu], [1,2-propanediol], [ethanol], and [1-propanol] (g/L) were determined from adapted method given by Zaky *et al.* [37] using a high-performance liquid chromatography (HPLC) (Model 1260 Infinity, Agilent Technologies, USA) equipped with C8 column (7.7 ×300 mm, 8 mm) (Hi-Plex H, Agilent Technologies) at 50°C. The retention times (RT) in HPLC chromatogram for these compounds were 10.1 – 10.2, 17.6 – 17.8, 21.8 – 22.0, and 28.1 – 28.3 min, respectively. Mobile phase was 5 mM H_2SO_4 with flow rate of 0.60 mL / min and run time of 27 min. Refractive index detector (RID) (Model G1362A, Agilent Technologies, USA) was used at 32°C. The HPLC analyses of [butyric acid], [LA], [formic acid], [acetic acid], and [succinic acid] (g/L) were based on the modified methods described by Scherer *et al.* [38] and da Costa *et al.* [39]. The similar system set up as Glu and ethanol analyses was used with 65°C column temperature, 0.85 mL / min flow rate, 32 min run time, and detection using diode array detector (DAD) (Model G1315B, Agilent Technologies, USA). RT of these five acids were 8.7 – 8.8, 9.2 – 9.3, 9.7 – 9.8, 10.7 – 10.8, and 15.9 – 16.1 min, respectively. The wavelength of DAD was set at 210 nm. Solubilized [IP6] was also determined by HPLC using IP6-Na as standard and calculation basis. The analytical C18 column (4.6 × 250 mm, 3.5 mm) (Zorbax, Agilent Technologies, USA) was maintained at 50°C while RID at 32°C was used as the

detector for analysis of [IP6]. Mobile phase was 10 mM acetate buffer with flow rate of 0.60 mL / min. Run time of 27 min was applied with RT of IP6 during 2.7 – 2.8 min [40]. The integrated area resulting in [IP6] was thus interpreted as overall concentration of low and high inositol Pi species [41].

A4: The mass balance calculation of produced [Pi] to the consumed [IP6] could be achieved by utlizing molar mass ratio of Pi and IP6-Na. Since the latter was used as a calculation basis for IP6, the corresponding molar mass ratio would be (6 × 94.97 g Pi / mol) / (923.82 g IP6-Na / mol) = 0.6168 g Pi / g IP6 (IP6-Na equivalent). Dividing the result of produced [Pi] by 0.6168 would thus result in the consumed [IP6]. The mass balance percentage of [IP6] on [Pi] production in IP6 medium could be determined from 100 × ([IP6] + consumed [IP6]) divided by [IP6] at time zero. By assuming complete mass balance closure for production of [Pi] from combined $[IP6]_{sol}$ and $[IP6]_{in\text{-}sol}$ in MsRB and WsRB media, $[IP6]_{in\text{-}sol}$ could then be calculated and combined with $[IP6]_{sol}$ to produce $[IP6]_{overall}$.

A5: The reaction mixture for PE_{act} assay comprised 400 μL of 0.1 M sodium acetate buffer at pH 5.5 with 1.2 mM IP6-Na as substrate. The collected and properly diluted enzyme sample of 200 μL was added to the reaction mixture and incubated for 30 min at 50°C before addition of 100 μL of 2 g/L trichloroacetic acid to stop the reaction [44, 45]. The solid precipitate was removed by centrifugation at 2,822 × g for 5 min prior to proper dilution of supernatant for subsequent [Pi] quantification. Volumetric PE_{act} (Vol-PE_{act} in U/mL) could then be computed from the amount of liberated Pi and implemented volume of enzyme sample in PE_{act} assay for both extracellular (ExVol-PE_{act}) and intracellular (InVol-PE_{act}) types. The latter was determined from glass bead - pretreated sample as described in Supplementary Section A2. Total protein concentration ([Tprot] in mg/mL) at physiological condition for both extracellular ([Ex-Tprot]) and intracellular ([In-Tprot]) types were determined based on Bradford assay [2, 3, 46, 47] using 0 - 2 mg/mL bovine serum albumin as standard solutions. Specific PE_{act} (U/$mg_{protein}$) was then determined from the ratio of Vol-PE_{act} and [Tprot] [48] resulting in either ExSp-PE_{act} from the ratio of ExVol-PE_{act} and [Ex-Tprot]) or InSp-PE_{act} from the ratio of InVol-PE_{act} and [In-Tprot].

A6: In order to quantitatively compare and assess the overall efficiency and productivity of all five LAB strains in producing Pi, LA, and PE_{act} from available IP6 in IP6, MsRB, and WsRB media during different cultivation periods. Up to thirteen items of kinetic data and parameters from each cultivation medium system These items include; (1) produced [Pi]; (2) mass balance of [IP6] on [Pi] production; (3) via-CD; (4) produced [LA] (condition: this score was not determined for medium in which all LAB strains produced less than 1 g/L [LA] throughout 72 h cultivation period); (5) $Y_{LA/Glu}$ (condition: this score was determined only for IP6 medium based on rationale described in section 2.5); (6, 7) produced ExVol-PE_{act} and ExSp-PE_{act}; (8, 9) produced InVol-PE_{act} and InSp-PE_{act}; (10) m; (11) $q_{s,Glu}$; (12) $q_{p,Pi}$; and (13) $q_{p,LA}$. The highest absolute value of each item was set to 100 before converting the lesser values proportionally to the maximum value. Score normalisation of each item was carried out internally within each type of medium and not across the board for all media due to varying heterogeniety and initial composition of specific media which might trigger different growth and metabolic responses. The combination of scores from items (1) – (9) for each time point was normalised to 100 resulting in summation of kinetic data scores (D_{Sc}) with the maximum

possible score of 4 time points × 100 = 400. The subtotal D_{Sc} for each time point could then be used as the selection criterion for the optimal cultivation time of each LAB strain. Similar combination and normalisation of scores were performed for items (10) – (13) resulting in summation of kinetic parameter scores (P_{Sc}) during three intervals (0 – 24, 24 – 48, and 48 – 72 h) with the maximum possible score of 300. D_{Sc} and P_{Sc} scores were then further combined additively by either equivalent weighting (D_{Sc} + P_{Sc}) or 2 : 1 weighting ratio (2D_{Sc} + P_{Sc}) based on the selection criterion that emphasising kinetic data ([chemical species], $Y_{LA/Glu}$, and PE_{act}) in a greater extent than the kinetic parameters. The maximum possible scores for D_{Sc} + P_{Sc} and 2D_{Sc} + P_{Sc} summation were thus 400 + 300 = 700 and 2 × 400 + 300 = 1,100, respectively.

Supplementary Section B

The numbering system of Tables in this section is "Sx.y" where; x indicates different type of cultivation medium, namely, 1 for IP6, 2 for MsRB, and 3 for WsRB; y = 1 to 7 with tabulated results of each LAB strain cultivation in order of appearance as shown in Table S0.

Table S0: Directory of LAB strains cultivation results using "Sx.y" format with respect to each y value

y value	Tabulated results
1	[Glu], [IP6], produced [Pi], mass balance percentage of [IP6] on [Pi] production, via-CD
2	Produced [LA], produced [formic acid], produced [acetic acid], produced [ethanol], pH level
3	$Y_{LA/Glu}$, produced ExVol-PE_{act}, produced ExSp-PE_{act}, produced InVol-PE_{act}, produced InSp-PE_{act}
4	Produced [Pi] scores (Item 1), mass balance percentage of [IP6] on [Pi] production scores (Item 2), via-CD scores (Item 3), produced [LA] scores (Item 4), $Y_{LA/Glu}$ scores (Item 5)
5	Produced ExVol-PE_{act} scores (Item 6), produced ExSp-PE_{act} scores (Item 7), produced InVol-PE_{act} scores (Item 8), produced InSp-PE_{act} scores (Item 9), overall normalised scoring to 100 for summation of Item 1 – 9 from Table S1.4 and S1.5, D_{Sc}
6	m, $q_{s,Glu}$, $q_{p,Pi}$, $q_{p,LA}$
7	m scores (Item 10), $q_{s,Glu}$ scores (Item 11), $q_{p,Pi}$ scores (Item 12), $q_{p,LA}$ scores (Item 13), overall normalised scoring to 100 for summation of Item 10 – 13, P_{Sc}, D_{Sc} + P_{Sc}, 2D_{Sc} + P_{Sc}

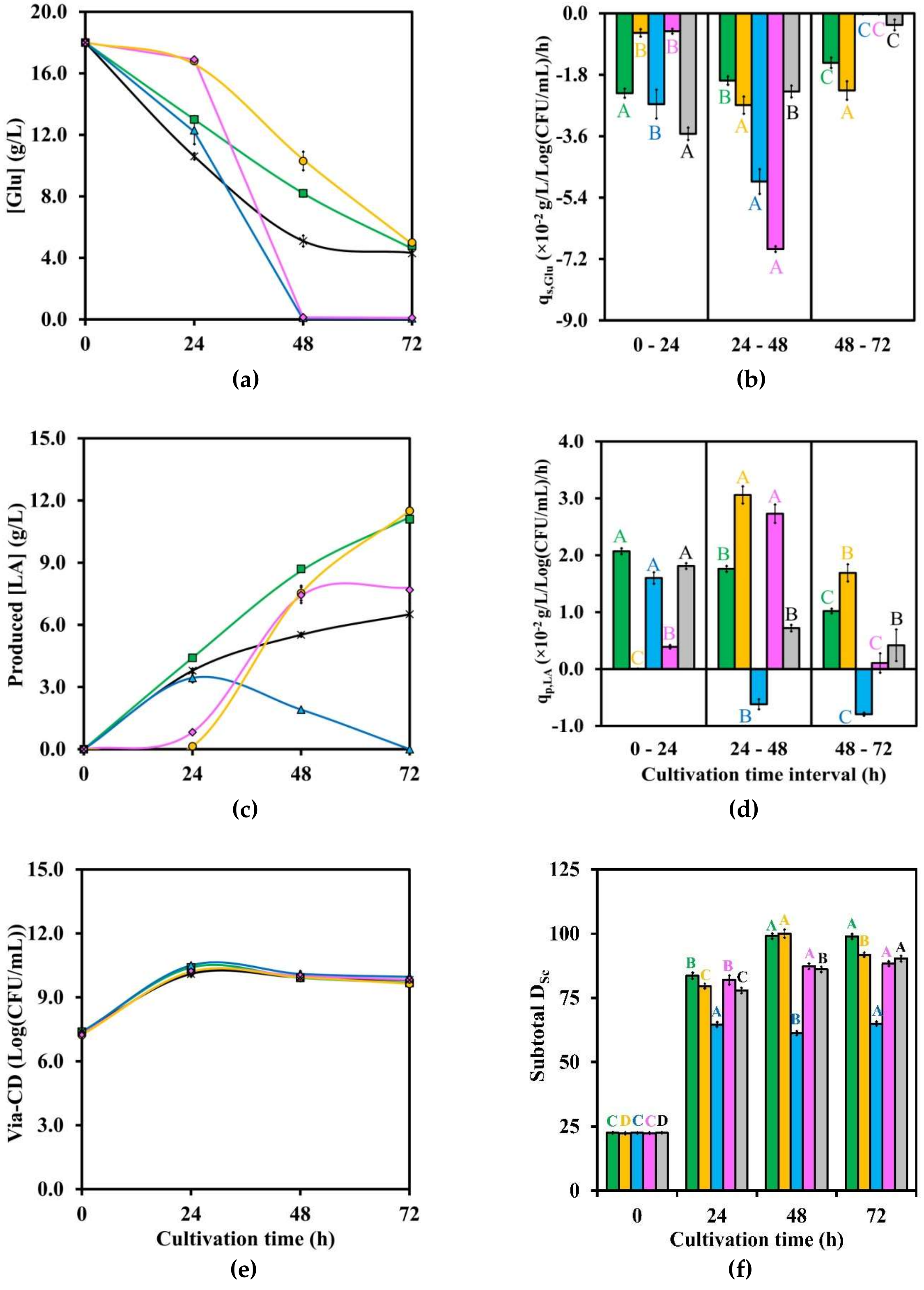

Figure S1: Kinetic data and parameters profiles as well as normalised weighting scores of five LAB strains (TISTR ■ 1500; ● 877; ▲ 890; ◆ 055; × 1498) during 72 h cultivation time in IP6 medium with respect to (a) [Glu]; (b) $q_{s,Glu}$; (c) produced [LA]; (d) $q_{p,LA}$; (e) via-CD; and (f) subtotal D_{Sc}. Each SE was included as an error bar to each data point. The tabulated average and error results values for each LAB strain cultivation with stastistical significant comparison between time points of (a) were in Supplementary Section B Table S1.1; (b) in Table S1.6; (c) in Table S1.2; (d) in Table S1.6; (e) in Table S1.1; and (f) in Table S1.5. The statistically significant comparison in (b), (d), and (f) were made across three cultivation time intervals or four cultivation time periods for each LAB strain with

similar font coloring. The numbers with the same alphabet (A - D) indicated no statistically significant difference ($p > 0.05$).

Table S1.1: Detailed kinetics data for five LAB strains cultivation in IP6 medium during 0 – 72 h with respect to [Glu], [IP6], produced [Pi], mass balance percentage of [IP6] on [Pi] production, and via-CD.

**Cultivation time (h)**	**LAB strains**				
	***Lacticaseibacillus casei* TISTR 1500**	***Lactiplantibacillus plantarum* TISTR 877**	***Latilactobacillus sakei* TISTR 890**	***Limosilactobacillus fermentum* TISTR 055**	***Weissella confusa* TISTR 1498**
**[Glu] (g/L)**					
**0**	**$18.0^{A} \pm 0.2$**	**$18.0^{A} \pm 0.2$**	**$18.0^{A} \pm 0.1$**	**$18.0^{A} \pm 0.1$**	**$18.0^{A} \pm 0.3$**
**24**	$13.0^{B} \pm 0.2$	$16.8^{B} \pm 0.1$	$12.3^{B} \pm 0.9$	$16.9^{B} \pm 0.1$	$10.6^{B} \pm 0.2$
**48**	$8.20^{C} \pm 0.20$	$10.3^{C} \pm 0.6$	$0.11^{C} \pm < 0.01$	$0.14^{C} \pm < 0.01$	$5.10^{C} \pm 0.35$
**72**	$4.80^{D} \pm 0.30$	$5.00^{D} \pm 0.20$	$0.07^{C} \pm < 0.01$	$0.10^{C} \pm < 0.01$	$4.30^{D} \pm 0.10$
**[IP6] (g/L)**					
**0**	**$4.54^{A} \pm 0.02$**	**$4.54^{A} \pm 0.02$**	**$4.54^{A} \pm 0.02$**	**$4.54^{A} \pm 0.02$**	**$4.54^{A} \pm 0.02$**
**24**	$4.08^{B} \pm 0.03$	$4.11^{B} \pm 0.07$	$4.04^{B} \pm 0.04$	$3.75^{B} \pm 0.03$	$4.07^{B} \pm 0.03$
**48**	$3.82^{C} \pm 0.02$	$3.81^{C} \pm 0.05$	$4.00^{B} \pm 0.04$	$3.58^{C} \pm 0.03$	$3.95^{C} \pm 0.04$
**72**	$3.61^{D} \pm 0.02$	$3.73^{C} \pm 0.06$	$3.82^{C} \pm 0.04$	$3.36^{D} \pm 0.02$	$3.75^{D} \pm 0.02$
**Produced [Pi] (g/L)**					
**0**	$0.00^{D} \pm 0.00$	$0.00^{D} \pm 0.00$	$0.00^{D} \pm 0.00$	$0.00^{D} \pm 0.00$	$0.00^{D} \pm 0.00$
**24**	$0.27^{C} \pm 0.03$	$0.23^{C} \pm 0.02$	$0.24^{C} \pm 0.01$	$0.48^{C} \pm 0.02$	$0.24^{C} \pm 0.02$
**48**	$0.44^{B} \pm 0.01$	$0.43^{B} \pm 0.01$	$0.31^{B} \pm 0.02$	$0.58^{B} \pm 0.02$	$0.33^{B} \pm 0.04$
**72**	**$0.55^{A} \pm 0.01$**	**$0.50^{A} \pm 0.01$**	**$0.44^{A} \pm 0.02$**	**$0.70^{A} \pm 0.01$**	**$0.47^{A} \pm 0.01$**
**Mass balance percentage of [IP6] on [Pi] production (%)**					
**0**	**$100.0^{A} \pm 0.4$**	**$100.0^{A} \pm 0.4$**	**$100.0^{A} \pm 0.4$**	**$100.0^{A} \pm 0.4$**	**$100.0^{A} \pm 0.4$**
**24**	**$99.4^{A} \pm 0.8$**	**$98.6^{A} \pm 2.0$**	$97.4^{B} \pm 1.1$	**$99.5^{A} \pm 1.0$**	$98.0^{B} \pm 0.7$
**48**	**$99.7^{A} \pm 0.6$**	**$99.0^{A} \pm 1.8$**	**$99.1^{AB} \pm 1.2$**	**$99.6^{A} \pm 0.9$**	**$98.7^{A,B} \pm 1.1$**
**72**	**$99.1^{A} \pm 0.6$**	**$99.8^{A} \pm 1.8$**	**$99.7^{A} \pm 1.3$**	**$98.8^{A} \pm 0.8$**	**$99.2^{A,B} \pm 0.6$**
**Via-CD (Log (CFU/mL))**					
**0**	$7.39^{C} \pm 0.18$	$7.24^{D} \pm 0.20$	$7.36^{C} \pm 0.18$	$7.28^{C} \pm 0.18$	$7.38^{B} \pm 0.18$
**24**	**$10.4^{A} \pm 0.06$**	**$10.2^{A} \pm 0.12$**	**$10.5^{A} \pm 0.05$**	**$10.2^{A} \pm 0.12$**	**$10.1^{A} \pm 0.13$**
**48**	$9.92^{B} \pm 0.15$	$9.94^{B} \pm 0.12$	$10.1^{B} \pm 0.13$	**$10.0^{A,B} \pm 0.16$**	**$9.94^{A} \pm 0.15$**
**72**	$9.68^{B} \pm 0.12$	$9.64^{C} \pm 0.09$	$9.96^{B} \pm 0.15$	$9.83^{B} \pm 0.13$	**$9.81^{A} \pm 0.17$**

Note: The numbers with the same alphabet (A - D) indicate no statistically significant difference ($p > 0.05$) for comparison between time course subgroup of each LAB strain with respect to each via-CD or chemical species being monitored. Mass

balance percentage of [IP6] on [Pi] production in each data set might be subjected to slight rounding off error. Results which are bolded and underlined indicate the statistically significant highest values ($p \leq 0.05$) within the subgroup. Each result for 0th h cultivation time was obtained immediately after inoculum addition and well mixing.

Table S1.2: Detailed kinetics data for five LAB strains cultivation in IP6 medium during 0 – 72 h with respect to produced [LA], produced [formic acid], produced [acetic acid], produced [ethanol], and pH level. Production of butyric acid, succinic acid, 1-propanol, 1,2-propanediol were not detected in all cases.

Cultivation time (h)	LAB strains				
	Lacticaseibacillus casei TISTR 1500	*Lactiplantibacillus plantarum* TISTR 877	*Latilactobacillus sakei* TISTR 890	*Limosilactobacillus fermentum* TISTR 055	*Weissella confusa* TISTR 1498
**Produced [LA] (g/L)**					
**0**	$0.00^{D} \pm 0.00$	$0.00^{C} \pm 0.00$	$0.00^{C} \pm 0.00$	$0.00^{C} \pm 0.00$	$0.00^{D} \pm 0.00$
**24**	$4.41^{C} \pm 0.10$	$0.14^{C} \pm 0.01$	**$3.44^{A} \pm 0.21$**	$0.82^{B} \pm 0.06$	$3.79^{C} \pm 0.10$
**48**	$8.70^{B} \pm 0.07$	$7.53^{B} \pm 0.35$	$1.91^{B} \pm 0.06$	**$7.44^{A} \pm 0.38$**	$5.52^{B} \pm 0.09$
**72**	**$11.1^{A} \pm 0.06$**	**$11.5^{A} \pm 0.07$**	$0.00^{C} \pm 0.00$	**$7.69^{A} \pm 0.14$**	**$6.51^{A} \pm 0.65$**
**Produced [formic acid] (g/L)**					
**0**	$0.00^{B} \pm 0.00$	$0.00^{C} \pm 0.00$	$0.00^{D} \pm 0.00$	$0.00^{C} \pm 0.00$	$0.00^{D} \pm 0.00$
**24**	**$0.11^{A} \pm 0.02$**	$0.27^{B} \pm 0.02$	$0.15^{C} \pm 0.02$	$0.19^{B} \pm 0.03$	$0.47^{C} \pm 0.05$
**48**	**$0.12^{A} \pm 0.01$**	**$0.37^{A} \pm 0.05$**	$0.42^{B} \pm 0.05$	$0.18^{B} \pm 0.02$	$0.61^{B} \pm 0.04$
**72**	$0.00^{B} \pm 0.00$	$0.26^{B} \pm 0.03$	**$0.62^{A} \pm 0.04$**	**$0.43^{A} \pm 0.04$**	**$0.91^{A} \pm 0.02$**
**Produced [acetic acid] (g/L)**					
**0**	$0.00^{C} \pm 0.00$	$0.00^{D} \pm 0.00$	$0.00^{D} \pm 0.00$	$0.00^{C} \pm 0.00$	$0.00^{C} \pm 0.00$
**24**	$0.04^{C} \pm 0.01$	$0.50^{C} \pm 0.04$	$0.62^{C} \pm 0.07$	$0.34^{B} \pm 0.02$	$2.20^{B} \pm 0.19$
**48**	$0.09^{B} \pm 0.02$	$0.96^{B} \pm 0.04$	$2.85^{B} \pm 0.19$	**$0.81^{A} \pm 0.04$**	**$5.15^{A} \pm 0.20$**
**72**	**$0.52^{A} \pm 0.04$**	**$1.69^{A} \pm 0.11$**	**$4.23^{A} \pm 0.28$**	**$0.87^{A} \pm 0.06$**	**$5.38^{A} \pm 0.49$**
**Produced [ethanol] (g/L)**					
**0**	$0.00^{NS} \pm 0.00$	$0.00^{NS} \pm 0.00$	$0.00^{NS} \pm 0.00$	$0.00^{C} \pm 0.00$	$0.00^{D} \pm 0.00$
**24**	$0.00^{NS} \pm 0.00$	$0.00^{NS} \pm 0.00$	$0.00^{NS} \pm 0.00$	$0.00^{C} \pm 0.00$	$0.70^{C} \pm 0.03$
**48**	$0.00^{NS} \pm 0.00$	$0.00^{NS} \pm 0.00$	$0.00^{NS} \pm 0.00$	$2.76^{B} \pm 0.10$	$0.99^{B} \pm 0.07$
**72**	$0.00^{NS} \pm 0.00$	$0.00^{NS} \pm 0.00$	$0.00^{NS} \pm 0.00$	**$3.64^{A} \pm 0.04$**	**$1.54^{A} \pm 0.10$**
**pH level**					
**0**	**$5.60^{A} \pm 0.01$**	**$5.69^{A} \pm 0.01$**	**$5.58^{A} \pm 0.01$**	**$5.68^{A} \pm 0.01$**	**$5.65^{A} \pm 0.01$**
**24**	$4.49^{B} \pm 0.01$	$5.38^{B} \pm 0.01$	$4.70^{D} \pm 0.01$	$5.44^{B} \pm 0.01$	$4.64^{B} \pm 0.01$
**48**	$4.05^{C} \pm 0.01$	$4.26^{C} \pm 0.01$	$4.77^{C} \pm 0.01$	$4.31^{C} \pm 0.01$	$4.34^{C} \pm 0.01$
**72**	$3.92^{D} \pm 0.01$	$3.89^{D} \pm 0.01$	$5.03^{B} \pm 0.01$	$4.30^{C} \pm 0.01$	$4.35^{C} \pm 0.01$

Note: The numbers with the same alphabet (A -D) indicate no statistically significant difference ($p > 0.05$) for comparison between time course subgroup of each LAB strain with respect to each chemical species being monitored. Results which are bolded and underlined indicate the statistically significant highest values ($p \leq 0.05$) within the subgroup. Each result for 0th h cultivation time was obtained immediately after inoculum addition and well mixing. The initial pH level before addition of inoculum, which contained slightly produced acids, in each case was 6.00 ± 0.01.

Table S1.3: Detailed kinetics data for five LAB strains cultivation in IP6 medium during 0 – 72 h with respect to $Y_{LA/Glu}$, produced ExVol-PE_{act}, produced ExSp-PE_{act}, produced InVol-PE_{act}, and produced InSp-PE_{act}.

Cultivation time (h)	LAB strains				
	Lacticaseibacillus casei TISTR 1500	*Lactiplantibacillus plantarum* TISTR 877	*Latilactobacillus sakei* TISTR 890	*Limosilactobacillus fermentum* TISTR 055	*Weissella confusa* TISTR 1498
**$Y_{LA/Glu}$ (g $LA_{produced}$ / g $Glu_{consumed}$)**					
**0**	n.d.	n.d.	n.d.	n.d.	n.d.
**24**	**0.88^{A} ± 0.05**	0.12^{C} ± 0.02	**0.60^{A} ± 0.05**	**0.75^{A} ± 0.11**	**0.51^{A} ± 0.03**
**48**	**0.89^{A} ± 0.03**	**0.98^{A} ± 0.09**	0.11^{B} ± 0.01	0.42^{B} ± 0.02	0.43^{B} ± 0.02
**72**	**0.84^{A} ± 0.02**	0.88^{B} ± 0.02	n.d.	0.43^{B} ± 0.01	**0.48^{A} ± 0.03**
**Produced ExVol-PE_{act} (U / mL)**					
**0**	0.0000^{D} ± 0.0000	0.0000^{D} ± 0.0000	0.0000^{D} ± 0.0000	0.0000^{C} ± 0.0000	0.0000^{D} ± 0.0000
**24**	0.0579^{C} ± 0.0020	**0.0822^{A} ± 0.0010**	0.0382^{C} ± 0.0010	**0.0907^{A} ± 0.0010**	0.0516^{C} ± 0.0010
**48**	**0.0756^{A} ± 0.0030**	0.0650^{B} ± 0.0030	0.0583^{B} ± 0.0010	**0.0927^{A} ± 0.0010**	0.0560^{B} ± 0.0010
**72**	0.0679^{B} ± 0.0030	0.0375^{C} ± 0.0020	**0.0812^{A} ± 0.0020**	0.0846^{B} ± 0.0020	**0.0604^{A} ± 0.0010**
**Produced ExSp-PE_{act} (U / $mg_{protein}$)**					
**0**	0.000^{D} ± 0.000	0.000^{D} ± 0.000	0.000^{D} ± 0.000	0.000^{B} ± 0.000	0.000^{D} ± 0.000
**24**	0.176^{C} ± 0.006	**0.275^{A} ± 0.004**	0.091^{C} ± 0.003	**0.163^{A} ± 0.002**	0.210^{C} ± 0.003
**48**	**0.278^{A} ± 0.011**	0.240^{B} ± 0.010	0.135^{B} ± 0.007	**0.163^{A} ± 0.002**	0.261^{B} ± 0.006
**72**	0.225^{B} ± 0.010	0.129^{C} ± 0.008	**0.167^{A} ± 0.008**	**0.167^{A} ± 0.003**	**0.283^{A} ± 0.006**
**Produced InVol-PE_{act} (U / mL)**					
**0**	0.0000^{B} ± 0.0000	0.0000^{C} ± 0.0000	0.0000^{C} ± 0.0000	0.0000^{B} ± 0.0000	0.0000^{B} ± 0.0000
**24**	**0.0213^{A} ± 0.0010**	**0.0257^{A} ± 0.0010**	**0.0212^{A} ± 0.0010**	**0.0277^{A} ± 0.0014**	**0.0219^{A} ± 0.0010**
**48**	**0.0225^{A} ± 0.0010**	0.0236^{B} ± 0.0010	0.0190^{B} ± 0.0010	**0.0290^{A} ± 0.0016**	**0.0221^{A} ± 0.0009**
**72**	**0.0210^{A} ± 0.0010**	**0.0270^{A} ± 0.0010**	**0.0202^{A,B} ± 0.0010**	**0.0272^{A} ± 0.0016**	**0.0207^{A} ± 0.0010**
**Produced InSp-PE_{act} (U / $mg_{protein}$)**					
**0**	0.000^{B} ± 0.000	0.000^{D} ± 0.000	0.000^{C} ± 0.000	0.000^{C} ± 0.000	0.000^{D} ± 0.000
**24**	**0.756^{A} ± 0.047**	0.934^{B} ± 0.041	**0.227^{A} ± 0.005**	**0.229^{A} ± 0.011**	0.775^{C} ± 0.048
**48**	**0.751^{A} ± 0.037**	**1.090^{A} ± 0.046**	**0.222^{A} ± 0.006**	0.192^{B} ± 0.005	**0.984^{A} ± 0.025**
**72**	**0.780^{A} ± 0.041**	0.689^{C} ± 0.014	0.196^{B} ± 0.005	**0.231^{A} ± 0.014**	0.890^{B} ± 0.031

Note: The numbers with the same alphabet (A - D) indicate no statistically significant difference ($p > 0.05$) for comparison between time course subgroup of each LAB strain with respect to $Y_{LA/Glu}$ and each type of produced PE_{act}. $Y_{LA/Glu}$ was not determined (n.d.) for some time points due to insignificant consumed [Glu] and produced [LA]. Results which are bolded and underlined indicate the statistically significant highest values ($p \leq 0.05$) within the subgroup. Each result for 0th h cultivation time was obtained immediately after inoculum addition and well mixing.

Table S1.4: Detailed kinetics data scores from Table S1.1 – S1.3 for five LAB strains cultivation in IP6 medium during 0 – 72 h with respect to produced [Pi], mass balance percentage of [IP6] on [Pi] production, via-CD, produced [LA], and $Y_{LA/Glu}$.

Cultivation time (h)	LAB strains				
	Lacticaseibacillus casei TISTR 1500	*Lactiplantibacillus plantarum* TISTR 877	*Latilactobacillus sakei* TISTR 890	*Limosilactobacillus fermentum* TISTR 055	*Weissella confusa* TISTR 1498
**Produced [Pi] scores (Item 1)**					
**0**	0.00^{D} ± 0.00	0.00^{D} ± 0.00	0.00^{D} ± 0.00	0.00^{D} ± 0.00	0.00^{D} ± 0.,00
**24**	38.6^{C} ± 4.3	32.9^{C} ± 2.9	34.3^{C} ± 1.4	68.6^{C} ± 2.9	34.3^{C} ± 2.9
**48**	62.9^{B} ± 1.4	61.4^{B} ± 1.4	44.3^{B} ± 2.9	82.9^{B} ± 2.9	47.1^{B} ± 5.7
**72**	**78.6^{A} ± 1.4**	**71.4^{A} ± 1.4**	**62.9^{A} ± 2.9**	**100.0^{A} ± 1.4**	**67.1^{A} ± 1.4**
**Mass balance percentage of [IP6] on [Pi] production scores (Item 2)**					
**0**	**100.0^{A} ± 0.4**	**100.0^{A} ± 0.4**	**100.0^{A} ± 0.4**	**100.0^{A} ± 0.4**	**100.0^{A} ± 0.4**
**24**	**99.4^{A} ± 0.8**	**98.6^{A} ± 2.0**	97.4^{B} ± 1.1	**99.5^{A} ± 1.0**	98.0^{B} ± 0.7
**48**	**99.7^{A} ± 0.6**	**99.0^{A} ± 1.8**	**99.1^{AB} ± 1.2**	**99.6^{A} ± 0.9**	**$98.7^{A,B}$ ± 1.1**
**72**	**99.1^{A} ± 0.6**	**99.8^{A} ± 1.8**	**99.7^{A} ± 1.3**	**98.8^{A} ± 0.8**	**$99.2^{A,B}$ ± 0.6**
**Via-CD scores (Item 3)**					
**0**	70.4^{C} ± 1.7	69.0^{C} ± 1.9	70.1^{C} ± 1.7	69.3^{C} ± 1.7	70.3^{B} ± 1.7
**24**	**99.0^{A} ± 0.6**	**97.1^{A} ± 1.1**	**100.0^{A} ± 0.5**	**97.1^{A} ± 1.1**	**96.2^{A} ± 1.2**
**48**	94.5^{B} ± 1.4	**94.7^{A} ± 1.1**	96.2^{B} ± 1.2	**95.2^{AB} ± 1.5**	**94.7^{A} ± 1.4**
**72**	92.2^{B} ± 1.1	91.8^{B} ± 0.9	94.9^{B} ± 1.4	93.6^{B} ± 1.2	**93.4^{A} ± 1.6**
**Produced [LA] scores (Item 4)**					
**0**	0.00^{D} ± 0.00	0.00^{C} ± 0.00	0.00^{C} ± 0.00	0.00^{C} ± 0.00	0.00^{D} ± 0.00
**24**	38.4^{C} ± 0.9	1.22C ± 0.09	**29.9^{A} ± 1.8**	7.13^{B} ± 0.52	33.0^{C} ± 0.9
**48**	75.6^{B} ± 0.6	65.5^{B} ± 3.0	16.6^{B} ± 0.5	**64.7^{A} ± 3.3**	48.0^{B} ± 0.8
**72**	**96.5^{A} ± 0.5**	**100.0^{A} ± 0.61**	0.00^{C} ± 0.00	**66.9^{A} ± 1.2**	**56.6^{A} ± 5.6**
**$Y_{LA/Glu}$ scores (Item 5)**					
**0**	0.00^{B} ± 0.00	0.00^{D} ± 0.00	0.00^{C} ± 0.00	0.00^{C} ± 0.00	0.00^{C} ± 0.00
**24**	**89.8^{A} ± 5.1**	12.2^{C} ± 2.0	**61.2^{A} ± 5.10**	**76.5^{A} ± 11.2**	**52.0^{A} ± 3.1**
**48**	**90.8^{A} ± 3.1**	**100.0^{A} ± 9.2**	11.2^{B} ± 1.02	42.9^{B} ± 2.0	43.9^{B} ± 2.0
**72**	**85.7^{A} ± 2.0**	89.8^{B} ± 2.0	0.00^{C} ± 0.00	43.9^{B} ± 1.0	**49.0^{A} ± 3.1**

Note: The numbers with the same alphabet (A - D) indicate no statistically significant difference ($p > 0.05$) for comparison between time course subgroup of each LAB strain with respect to scores of each chemical species, via-CD, and $Y_{LA/Glu}$. Results which are bolded and underlined indicate the statistically significant highest values ($p \leq 0.05$) within the subgroup. The maximum score for each subgroup is 100 which is normalised using comparison across all five LAB strains.

Table S1.5: Detailed kinetics data scores from Table S1.3 for five LAB strains cultivation in IP6 medium during 0 – 72 h with respect to produced ExVol-PE$_{act}$, produced ExSp-PE$_{act}$, produced InVol-PE$_{act}$, and produced InSp-PE$_{act}$ as well as overall normalised scores from Table S1.4 – S1.5.

**Cultivation time (h)**	**LAB strains**				
	Lacticaseibacillus casei **TISTR 1500**	***Lactiplantibacillus plantarum*** **TISTR 877**	***Latilactobacillus sakei*** **TISTR 890**	***Limosilactobacillus fermentum*** **TISTR 055**	***Weissella confusa*** **TISTR 1498**
**Produced ExVol-PE$_{act}$ scores (Item 6)**					
**0**	0.00^{D} ± 0.00	0.00^{D} ± 0.00	0.00^{D} ± 0.00	0.00^{C} ± 0.00	0.00^{D} ± 0.00
**24**	62.5^{C} ± 2.2	**88.7^{A} ± 1.0**	41.2^{C} ± 1.2	97.8^{A} ± 1.1	55.7^{C} ± 0.7
**48**	**81.6^{A} ± 2.9**	70.1^{B} ± 3.0	62.9^{B} ± 1.1	**100.0^{A} ± 1.1**	60.4^{B} ± 1.1
**72**	73.2^{B} ± 2.8	40.5^{C} ± 2.6	**87.6^{A} ± 2.3**	91.2^{B} ± 1.8	**65.2^{A} ± 0.6**
**Produced ExSp-PE$_{act}$ scores (Item 7)**					
**0**	0.00^{D} ± 0.00	0.00^{D} ± 0.00	0.00^{D} ± 0.00	0.00^{C} ± 0.00	0.00^{D} ± 0.00
**24**	62.1^{C} ± 2.2	**97.3^{A} ± 1.3**	32.0^{C} ± 1.0	57.6^{B} ± 0.6	74.3^{C} ± 1.0
**48**	**98.3^{A} ± 4.0**	84.8^{B} ± 3.7	47.8^{B} ± 2.6	57.6^{B} ± 0.6	92.3^{B} ± 2.2
**72**	79.7^{B} ± 3.4	45.6^{C} ± 2.9	**59.1^{A} ± 2.7**	**59.1^{A} ± 1.2**	**100.0^{A} ± 2.0**
**Produced InVol-PE$_{act}$ scores (Item 8)**					
**0**	0.00^{B} ± 0.00	0.00^{C} ± 0.00	0.00^{C} ± 0.00	0.00^{B} ± 0.00	0.00^{B} ± 0.00
**24**	**73.4^{A} ± 3.4**	**88.7^{A} ± 3.4**	**73.1^{A} ± 3.4**	**95.5^{A} ± 4.7**	**75.4^{A} ± 3.4**
**48**	**77.6^{A} ± 3.4**	81.4^{B} ± 3.4	65.5^{B} ± 3.4	**100.0^{A} ± 5.5**	**76.2^{A} ± 3.2**
**72**	**72.3^{A} ± 3.4**	**93.0^{A} ± 3.4**	**69.5^{AB} ± 3.4**	**93.8^{A} ± 5.6**	**71.2^{A} ± 3.4**
**Produced InSp-PE$_{act}$ scores (Item 9)**					
**0**	0.00^{B} ± 0.00	0.00^{D} ± 0.00	0.00^{C} ± 0.00	0.00^{C} ± 0.00	0.00^{D} ± 0.00
**24**	**69.3^{A} ± 4.3**	85.8^{B} ± 3.7	**20.8^{A} ± 0.5**	**21.0^{A} ± 1.0**	71.1^{C} ± 4.4
**48**	**68.9^{A} ± 3.4**	**100.0^{A} ± 4.2**	**20.4^{A} ± 0.5**	17.6^{B} ± 0.5	**90.3^{A} ± 2.3**
**72**	**71.5^{A} ± 3.8**	63.2^{C} ± 1.2	18.0^{B} ± 0.4	**21.2^{A} ± 1.3**	81.7^{B} ± 2.9
**Overall normalised scoring to 100 for summation of Item 1 – 9 from Table S1.4 and S1.5**					
**0**	22.5^{C} ± 0.2	22.3^{D} ± 0.3	22.5^{C} ± 0.2	22.4^{C} ± 0.2	22.5^{D} ± 0.2
**24**	83.6^{B} ± 1.2	79.6^{C} ± 0.9	**64.7^{A} ± 0.9**	82.0^{B} ± 1.7	77.9^{C} ± 1.0
**48**	**99.1^{A} ± 1.0**	**100.0^{A} ± 1.6**	61.3^{B} ± 0.8	**87.3^{A} ± 1.0**	86.1^{B} ± 1.0
**72**	**98.9^{A} ± 1.0**	91.8^{B} ± 0.8	**65.0^{A} ± 0.8**	**88.3^{A} ± 0.9**	**90.3^{A} ± 1.1**
**Summation (D$_{Sc}$)**	**304^{a} ± 2**	294^{b} ± 2	214^{d} ± 1	280^{c} ± 2	277^{c} ± 2

Note: The numbers with the same capital alphabet (A - D) indicate no statistically significant difference ($p > 0.05$) for comparison between time course subgroup of each LAB strain with respect to each type of produced PE$_{act}$ score. Results which are bolded and underlined indicate the statistically significant highest values ($p \leq 0.05$) within the subgroup. The maximum score for each subgroup is 100 which is normalised using comparison across all five LAB strains. The summation row at the end of the table represents the summation of overall normalised scores at 0, 24, 48, and 72 h for each LAB strain with the maximum summation of overall normalised score of 400. The statistically significant difference comparsion between data of summation row was carried out at the similar p level with representation by small alphabets (a – d).

Table S1.6: Detailed kinetics parameters for five LAB strains cultivation in IP6 medium during three time intervals between 0 – 72 h with respect to m, $q_{s,Glu}$, $q_{p,Pi}$, and $q_{p,LA}$.

Cutivation time interval (h)	LAB strains				
	Lacticaseibacillus casei TISTR 1500	*Lactiplantibacillus plantarum* TISTR 877	*Latilactobacillus sakei* TISTR 890	*Limosilactobacillus fermentum* TISTR 055	*Weissella confusa* TISTR 1498
**m (× 10^{-2} per h)**					
**0 - 24**	**1.41^{A} ± 0.09**	**1.41^{A} ± 0.11**	**1.46^{A} ± 0.09**	**1.39^{A} ± 0.10**	**1.29^{A} ± 0.09**
**24 - 48**	-0.197^{B} ± 0.066*	-0.108^{B} ± 0.070*	-0.162^{B} ± 0.056*	-0.082^{B} ± 0.082*	-0.066^{B} ± 0.082*
**48 - 72**	-0.102^{B} ± 0.082*	-0.128^{B} ± 0.064*	-0.058^{B} ± 0.082*	-0.071^{B} ± 0.087*	-0.055^{B} ± 0.096*
**$q_{s,Glu}$ (× 10^{-2} g / L / Log(CFU/mL) / h)**					
**0 - 24**	**-2.34^{A} ± 0.13**	-0.573^{B} ± 0.107*	-2.66^{B} ± 0.42	-0.524^{B} ± 0.068*	**-3.53^{A} ± 0.18**
**24 - 48**	-1.97^{B} ± 0.12	**-2.69^{A} ± 0.25**	**-4.93^{A} ± 0.36**	**-6.91^{A} ± 0.08**	-2.29^{B} ± 0.17
**48 - 72**	-1.45^{C} ± 0.15	**-2.26^{A} ± 0.27**	-0.017^{C} ± 0.006*	-0.017^{C} ± 0.006*	-0.338^{C} ± 0.154*
**$q_{p,Pi}$ (× 10^{-3} g / L / Log(CFU/mL) / h)**					
**0 - 24**	**1.26^{A} ± 0.14**	**1.10^{A} ± 0.10**	**1.12^{A} ± 0.05**	**2.29^{A} ± 0.10**	**1.14^{A} ± 0.10**
**24 - 48**	0.697^{B} ± 0.130*	0.828^{B} ± 0.009	0.283^{C} ± 0.090*	0.412^{B} ± 0.117*	0.374^{B} ± 0.186*
**48 - 72**	0.468^{B} ± 0.060*	0.298^{C} ± 0.006	0.540^{B} ± 0.118*	0.504^{B} ± 0.094*	0.591^{B} ± 0.174*
**$q_{p,LA}$ (× 10^{-2} g / L / Log(CFU/mL) / h)**					
**0 - 24**	**2.07^{A} ± 0.05**	0.007^{C} ± 0.001	**1.60^{A} ± 0.10**	0.391^{B} ± 0.029	**1.81^{A} ± 0.05**
**24 - 48**	1.76^{B} ± 0.05	**3.06^{A} ± 0.15**	-0.619^{B} ± 0.088*	**2.73^{A} ± 0.16**	0.719^{B} ± 0.056
**48 - 72**	1.02^{C} ± 0.04	1.69^{B} ± 0.15	-0.794^{C} ± 0.026	0.105^{C} ± 0.170*	0.418^{B} ± 0.277*

Note: The numbers with the same alphabet (A - C) indicate no statistically significant difference ($p > 0.05$) for comparison between time interval subgroup of each LAB strain with respect to each specific rate being examined. All negative values were colored violet. The negative values of m indicate the mitigating via-CD as cultivation times progressed while the negative values of $q_{s,Glu}$ represent decreasing [Glu] level with time. The negative values of $q_{p,LA}$ represented degradation of [LA] with time. Results which are bolded and underlined indicate the statistically significant highest values ($p \leq 0.05$) within the subgroup.

* These SE might seem too large when compared to corresponding average values (> or >> 10%) but they were, in fact, correctly calculated through principle of error propagation. The resulting substraction of two values that were slightly changed between two time courses would inevitably fall within or beyond 100% range of uncertainties, for example, m of *Limosilactobacillus fermentum* TISTR 055 during cultivation time interval of 24 – 48 h was (-0.082 ± 0.082) × 10^{-2} per h or 100 × 0.082 × 10^{-2} / $|-0.082 \times 10^{-2}|$ = 100% uncertainty eventhough the parental values before subtraction has the relative error of less than 2% (via-CD of 10.2 ± 0.12 and 10.0 ± 0.16 Log (CFU/mL) at 24 and 48 h, respectively as shown in Table S1.1). The calculated average m for this case would be 10.0 – 10.2 / ((10.0 + 10.2)/2) / (48 – 24) h = -0.082 × 10^{-2} per h. The propagated error of via-CD formation rate ((10.0 – 10.2)/24 h = 0.833 × 10^{-2} Log (CFU/mL) / h) would be square root of summation between 0.12^2 and 0.16^2 divided by 48 – 24 = 24 h or $(0.12^2 + 0.16^2)^{0.5}$ / 24 h = 0.2 / 24 h = 0.833 × 10^{-2} Log (CFU/mL) / h. This calculated error would be propagated later with the error of average value between 10.0 and 10.2 Log (CFU/mL) which was $(0.12^2 + 0.16^2)^{0.5}$ / 2 = 0.02 Log (CFU/mL) for the average value of (10.0 + 10.2)/2 = 10.1 Log (CFU/mL). The overall propagated error for m in this case would thus be $|-0.082\times10^{-2}| \times ((0.833 \times 10^{-2} / 0.833 \times 10^{-2})^2 + (0.02 / 10.1)^2)^{0.5} = |-0.082\times10^{-2}| \times 1$ = 0.082 × 10^{-2} per h and hence m = (-0.082 ± 0.082) × 10^{-2} per h.

Table S1.7: Detailed kinetics parameters scores from Table S1.6 for five LAB strains cultivation in IP6 medium during three time intervals between 0 – 72 h with respect to m, $q_{s,Glu}$, $q_{p,Pi}$, and $q_{p,LA}$ as well as overall normalised scores.

Cultivation time Interval (h)	LAB strains				
	Lacticaseibacillus casei TISTR 1500	*Lactiplantibacillus plantarum* TISTR 877	*Latilactobacillus sakei* TISTR 890	*Limosilactobacillus fermentum* TISTR 055	*Weissella confusa* TISTR 1498
**m scores (Item 10)**					
**0 - 24**	**<u>96.2^{A} ± 6.2</u>**	**<u>96.5^{A} ± 7.7</u>**	**<u>100.0^{A} ± 6.0</u>**	**<u>95.0^{A} ± 7.1</u>**	**<u>88.5^{A} ± 7.3</u>**
**24 - 48**	-13.4^{B} ± 4.5	-7.34^{B} ± 4.79*	-11.0^{B} ± 3.8	5.63^{B} ± 5.63*	-4.54^{B} ± 5.63*
**48 - 72**	-6.96^{B} ± 5.57*	-8.71^{B} ± 4.36*	-3.97^{B} ± 5.63*	4.88^{B} ± 5.91*	-3.74^{B} ± 6.53*
**$q_{s,Glu}$ scores (Item 11)**					
**0 - 24**	**<u>33.9^{A} ± 2.0</u>**	8.29^{B} ± 1.55*	38.5^{B} ± 2.2	7.58^{B} ± 0.98*	**<u>51.0^{A} ± 2.6</u>**
**24 - 48**	28.5^{B} ± 1.7	**<u>38.9^{A} ± 3.6</u>**	**<u>71.3^{A} ± 2.2</u>**	**<u>100.0^{A} ± 1.0</u>**	33.1^{B} ± 2.4
**48 - 72**	20.9^{C} ± 2.2	**<u>32.6^{A} ± 3.9</u>**	0.24^{C} ± 0.09*	0.24^{C} ± 0.09*	4.88^{C} ± 2.22*
**$q_{p,Pi}$ scores (Item 12)**					
**0 - 24**	**<u>55.3^{A} ± 6.2</u>**	**<u>48.0^{A} ± 4.2</u>**	**<u>48.9^{A} ± 2.1</u>**	**<u>100.0^{A} ± 4.0</u>**	**<u>50.0^{A} ± 4.2</u>**
**24 - 48**	30.5^{B} ± 5.7*	36.2^{B} ± 4.0	12.4^{C} ± 4.0*	18.0^{B} ± 5.1*	16.4^{B} ± 8.1*
**48 - 72**	20.4^{B} ± 2.6*	13.0^{C} ± 2.6	23.6^{B} ± 5.1*	22.0^{B} ± 4.1*	25.8^{B} ± 7.6*
**$q_{p,LA}$ scores (Item 13)**					
**0 - 24**	**<u>67.6^{A} ± 1.7</u>**	2.19^{C} ± 0.16	**<u>52.5^{A} ± 3.2</u>**	12.8^{B} ± 1.0	**<u>59.1^{A} ± 1.7</u>**
**24 - 48**	57.5^{B} ± 1.7	**<u>100.0^{A} ± 5.0</u>**	-20.2^{B} ± 2.9*	**<u>89.3^{A} ± 5.3</u>**	23.5^{B} ± 1.8
**48 - 72**	33.4^{C} ± 1.3	55.3^{B} ± 5.0	-26.0^{C} ± 0.8	3.44^{C} ± 5.57*	13.7^{B} ± 9.1*
**Overall normalised scoring to 100 for summation of Item 10 – 13**					
**0 - 24**	**<u>100.0^{A} ± 4.0</u>**	**<u>61.3^{A} ± 3.5</u>**	**<u>94.8^{A} ± 3.7</u>**	**<u>85.2^{A} ± 3.4</u>**	**<u>98.3^{A} ± 3.6</u>**
**24 – 48**	40.7^{B} ± 3.0	**<u>66.3^{A} ± 3.4</u>**	20.7^{B} ± 3.2*	**<u>84.2^{A} ± 3.7</u>**	27.0^{B} ± 4.1*
**48 – 72**	26.8^{C} ± 2.6	36.4^{B} ± 3.2	-2.40^{C} ± 3.03*	12.1^{B} ± 3.6*	16.1^{C} ± 5.4*
**Summation S1.7 (P_{Sc})**	168^{b} ± 6	164^{b} ± 6	113^{d} ± 6	**<u>182^{a} ± 6</u>**	141^{c} ± 8
**Summation S1.5 + S1.7 (D_{Sc} + P_{Sc})**	**<u>472^{a} ± 6</u>**	458^{b} ± 6	327^{d} ± 6	**<u>462^{a,b} ± 6</u>**	418^{c} ± 8
**Summation 2 × S1.5 + S1.7 (2D_{Sc} + P_{Sc})**	**<u>776^{a} ± 7</u>**	751^{b} ± 7	540^{d} ± 6	742^{b} ± 8	695^{c} ± 8

Note: The numbers with the same capital alphabet (A - C) indicate no statistically significant difference ($p > 0.05$) for comparison between time interval subgroup of each LAB strain with respect to scoring of each specific rate being examined. Results which are bolded and underlined indicate the statistically significant highest values ($p \leq 0.05$) within the subgroup. The maximum score for each subgroup is 100 which is normalised using comparison across all five LAB strains. All negative values were colored violet. The summation rows at the end of the Table represent the summation of overall normalised scores at 0 - 24, 24 - 48, and 48 - 72 h from Table S1.7 (P_{Sc}); Table S1.5 and S1.7 with equal weighting (D_{Sc} + P_{Sc}), as well as Table S1.5 and S1.7 with double weighting of the former (2D_{Sc} + P_{Sc}) for each LAB strain with the maximum summation of overall normalised score of

300, 400 + 300 = 700, and 2 × 400 + 300 = 1,100, respectively. The statistically significant difference comparsion between data of these summation rows were carried out at the similar p level with representation by small alphabets (a – d).

* These SE were correctly propagated through mathematical operations, see footnote of Table S1.6 for rationale.

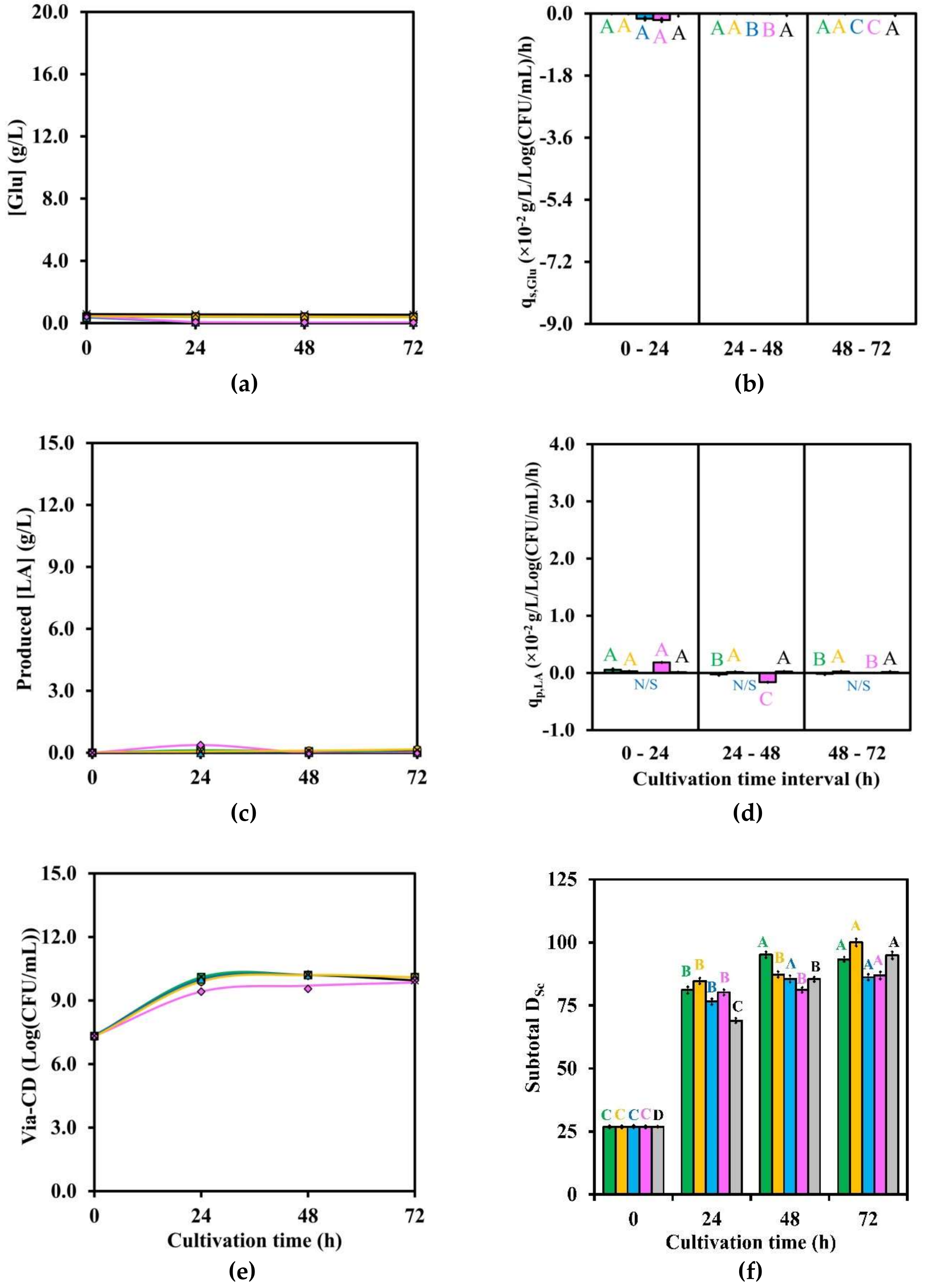

Figure S2: Kinetic data and parameters profiles as well as normalised weighting scores of five LAB strains (TISTR ■ 1500; ● 877; ▲ 890; ◆ 055; × 1498) during 72 h cultivation time in MsRB medium with respect to (a) [Glu]; (b) $q_{s,Glu}$; (c) produced [LA]; (d) $q_{p,LA}$; (e) via-CD; and (f) subtotal D_{Sc}. Each SE was included as an error bar to each data point. The tabulated average and error results values for each LAB strain cultivation with stastistical significant comparison between time points of (a) were in Supplementary Section B Table S2.1; (b) in Table S2.6; (c) in Table S2.2; (d) in Table S2.6; (e) in Table S2.1; and (f) in Table S2.5. The statistically significant comparison in (b), (d), and (f) were made across three cultivation time intervals or four cultivation time periods for each LAB strain with

similar font coloring. The numbers with the same alphabet (A - D) indicated no statistically significant difference ($p > 0.05$).

Table S2.1: Detailed kinetics data for five LAB strains cultivation in MsRB medium during 0 – 72 h with respect to [Glu], [IP6], produced [Pi], mass balance percentage of [IP6] on [Pi] production, and via-CD.

**Cultivation time (h)**	**LAB strains**				
	***Lacticaseibacillus casei* TISTR 1500**	***Lactiplantibacillus plantarum* TISTR 877**	***Latilactobacillus sakei* TISTR 890**	***Limosilactobacillus fermentum* TISTR 055**	***Weissella confusa* TISTR 1498**
			**[Glu] (g/L)**		
**0**	**$0.42^{A} \pm 0.01$**	**$0.44^{A} \pm 0.01$**	**$0.37^{A} \pm 0.01$**	**$0.44^{A} \pm 0.01$**	**$0.58^{A} \pm 0.01$**
**24**	**$0.41^{A,B} \pm 0.01$**	**$0.43^{A,B} \pm 0.01$**	$0.06^{B} \pm < 0.01$	$0.06^{B} \pm < 0.01$	$0.56^{B} \pm 0.01$
**48**	$0.40^{B,C} \pm 0.01$	$0.42^{B} \pm 0.01$	$0.04^{C} \pm < 0.01$	$0.04^{C} \pm < 0.01$	$0.55^{B,C} \pm 0.01$
**72**	$0.39^{C} \pm 0.01$	$0.40^{C} \pm 0.01$	$0.04^{C} \pm < 0.01$	$0.03^{D} \pm < 0.01$	$0.54^{C} \pm 0.01$
			**$[IP6]_{overall}$ (g/L)**		
**0**	**$2.27^{A} \pm 0.04$**	**$2.27^{A} \pm 0.04$**	**$2.27^{A} \pm 0.04$**	**$2.27^{A} \pm 0.04$**	**$2.27^{A} \pm 0.04$**
**24**	$1.79^{B} \pm 0.06$	$1.77^{B} \pm 0.05$	$2.12^{B} \pm 0.05$	$2.05^{B} \pm 0.05$	$1.81^{B} \pm 0.06$
**48**	$1.73^{B,C} \pm 0.04$	$1.65^{C} \pm 0.03$	$2.02^{C} \pm 0.03$	$2.03^{B} \pm 0.04$	$1.66^{C} \pm 0.04$
**72**	$1.68^{C} \pm 0.04$	$1.41^{D} \pm 0.03$	$2.02^{C} \pm 0.01$	$1.94^{C} \pm 0.03$	$1.53^{D} \pm 0.03$
			**Produced [Pi] (g/L)**		
**0**	$0.00^{D} \pm 0.00$	$0.00^{D} \pm 0.00$	$0.00^{D} \pm 0.00$	$0.00^{D} \pm 0.00$	$0.00^{D} \pm 0.00$
**24**	$0.30^{C} \pm < 0.01$	$0.31^{C} \pm < 0.01$	$0.09^{C} \pm < 0.01$	$0.13^{C} \pm < 0.01$	$0.28^{C} \pm < 0.01$
**48**	$0.33^{B} \pm < 0.01$	$0.38^{B} \pm < 0.01$	$0.15^{B} \pm < 0.01$	$0.15^{B} \pm < 0.01$	$0.38^{B} \pm < 0.01$
**72**	**$0.37^{A} \pm < 0.01$**	**$0.53^{A} \pm < 0.01$**	**$0.16^{A} \pm < 0.01$**	**$0.21^{A} \pm < 0.01$**	**$0.46^{A} \pm < 0.01$**
			**Mass balance percentage of $[IP6]_{overall}$ on [Pi] production (%)**		
**0**	**$100.0^{NS} \pm 2.8$**	**$100.0^{NS} \pm 2.8$**	**$100.0^{NS} \pm 2.8$**	**$100.0^{NS} \pm 2.8$**	**$100.0^{NS} \pm 2.8$**
**24**	**$100.0^{NS} \pm 3.4$**	**$100.0^{NS} \pm 3.1$**	**$100.0^{NS} \pm 2.9$**	**$100.0^{NS} \pm 3.1$**	**$100.0^{NS} \pm 3.4$**
**48**	**$100.0^{NS} \pm 2.7$**	**$100.0^{NS} \pm 2.4$**	**$100.0^{NS} \pm 2.4$**	**$100.0^{NS} \pm 2.7$**	**$100.0^{NS} \pm 2.7$**
**72**	**$100.0^{NS} \pm 2.7$**	**$100.0^{NS} \pm 2.4$**	**$100.0^{NS} \pm 2.1$**	**$100.0^{NS} \pm 2.4$**	**$100.0^{NS} \pm 2.4$**
			**Via-CD (Log (CFU/mL))**		
**0**	$7.32^{C} \pm 0.09$	$7.29^{C} \pm 0.10$	$7.36^{B} \pm 0.15$	$7.32^{C} \pm 0.11$	$7.34^{C} \pm 0.10$
**24**	$10.1^{B} \pm 0.03$	$9.90^{B} \pm 0.17$	**$10.0^{A} \pm 0.15$**	$9.42^{B} \pm 0.11$	**$10.1^{A,B} \pm 0.07$**
**48**	**$10.2^{A} \pm 0.02$**	**$10.2^{A} \pm 0.17$**	**$10.2^{A} \pm 0.17$**	$9.55^{B} \pm 0.12$	**$10.2^{A} \pm 0.02$**
**72**	$10.1^{B} \pm 0.03$	**$10.1^{A} \pm 0.22$**	**$10.1^{A} \pm 0.22$**	**$9.95^{A} \pm 0.08$**	$9.95^{B} \pm 0.10$

Note: $[IP6]_{overall}$ in RB was the combined $[IP6]_{sol}$ and $[IP6]_{in\text{-}sol}$ with assumption of mass balance enclosure. $[IP6]_{sol}$ for each time point for MsRB was between 0.34 – 0.41 g/L. Mass balance percentage of [IP6] on [Pi] production in each data set might be subjected to slight rounding off error. The numbers with the same alphabet (A – D or NS) indicate no statistically significant difference ($p > 0.05$) for comparison between time course subgroup of each LAB strain with respect to each via-CD or chemical species being monitored. Results which are bolded and underlined indicate the statistically significant highest values ($p \leq 0.05$) within the subgroup. Each result for 0th h cultivation time was obtained immediately after inoculum addition and well mixing.

Table S2.2: Detailed kinetics data for five LAB strains cultivation in MsRB medium during 0 – 72 h with respect to produced [LA], produced [formic acid], produced [acetic acid], produced [ethanol], and pH level. Production of butyric acid, succinic acid, 1-propanol, 1,2-propanediol were not detected in all cases.

Cultivation time (h)	LAB strains				
	Lacticaseibacillus casei **TISTR 1500**	***Lactiplantibacillus plantarum*** **TISTR 877**	***Latilactobacillus sakei*** **TISTR 890**	***Limosilactobacillus fermentum*** **TISTR 055**	***Weissella confusa*** **TISTR 1498**
**Produced [LA] (g/L)**					
**0**	0.00^{C} ± 0.00	0.00^{D} ± 0.00	0.00^{NS} ± 0.00	0.00^{B} ± 0.00	0.00^{D} ± 0.00
**24**	**0.12^{A} ± 0.04**	0.06^{C} ± 0.01	0.00^{NS} ± 0.00	**0.37^{A} ± 0.01**	0.03^{C} ± 0.01
**48**	**0.06^{A,B} ± 0.03**	0.10^{B} ± 0.02	0.00^{NS} ± 0.00	0.00^{B} ± 0.00	0.09^{B} ± 0.01
**72**	0.02^{B,C} ± 0.03	**0.16^{A} ± 0.01**	0.00^{NS} ± 0.00	0.00^{B} ± 0.00	**0.13^{A} ± 0.03**
**Produced [formic acid] (g/L)**					
**0**	0.00^{NS} ± 0.00	0.00^{NS} ± 0.00	0.00^{NS} ± 0.00	0.00^{NS} ± 0.00	0.00^{NS} ± 0.00
**24**	0.00^{NS} ± 0.00	0.00^{NS} ± 0.00	0.00^{NS} ± 0.00	0.00^{NS} ± 0.00	0.00^{NS} ± 0.00
**48**	0.00^{NS} ± 0.00	0.00^{NS} ± 0.00	0.00^{NS} ± 0.00	0.00^{NS} ± 0.00	0.00^{NS} ± 0.00
**72**	0.00^{NS} ± 0.00	0.00^{NS} ± 0.00	0.00^{NS} ± 0.00	0.00^{NS} ± 0.00	0.00^{NS} ± 0.00
**Produced [acetic acid] (g/L)**					
**0**	0.00^{C} ± 0.00	0.00^{B} ± 0.00	0.00^{C} ± 0.00	0.00^{D} ± 0.00	0.00^{D} ± 0.00
**24**	0.07^{B} ± 0.03	**0.29^{A} ± 0.05**	0.94^{B} ± 0.03	0.13^{C} ± 0.06	0.07^{C} ± 0.02
**48**	0.10^{B} ± 0.04	**0.26^{A} ± 0.03**	**1.54^{A} ± 0.05**	1.39^{B} ± 0.07	0.13^{B} ± 0.01
**72**	**0.37^{A} ± 0.09**	**0.26^{A} ± 0.04**	**1.57^{A} ± 0.03**	**2.00^{A} ± 0.04**	**0.33^{A} ± 0.03**
**Produced [ethanol] (g/L)**					
**0**	0.00^{D} ± 0.00	0.00^{D} ± 0.00	0.00^{C} ± 0.00	0.00^{C} ± 0.00	0.00^{D} ± 0.00
**24**	0.38^{C} ± 0.03	0.33^{C} ± 0.01	0.03^{B} ± 0.01	0.08^{B} ± 0.02	0.05^{C} ± 0.01
**48**	0.47^{B} ± 0.02	0.42^{B} ± 0.01	0.05^{B} ± 0.01	0.11^{B} ± 0.01	0.14^{B} ± 0.03
**72**	**1.96^{A} ± 0.03**	**0.91^{A} ± 0.04**	**0.08^{A} ± 0.01**	**0.14^{A} ± 0.01**	**0.20^{A} ± 0.01**
**pH level**					
**0**	**5.68^{A} ± 0.02**	**5.62^{A} ± 0.01**	**5.71^{A} ± 0.01**	**5.63^{A} ± 0.02**	**5.77^{A} ± 0.01**
**24**	5.09^{B} ± 0.01	5.12^{B} ± 0.01	5.54^{B} ± 0.01	5.23^{B} ± 0.01	5.54^{B} ± 0.01
**48**	4.73^{C} ± 0.02	4.84^{C} ± 0.01	5.02^{C} ± 0.01	4.88^{C} ± 0.01	5.02^{C} ± 0.01
**72**	4.31^{D} ± 0.01	4.31^{D} ± 0.01	4.75^{D} ± 0.01	4.46^{D} ± 0.01	4.75^{D} ± 0.01

Note: The numbers with the same alphabet (A - D) indicate no statistically significant difference ($p > 0.05$) for comparison between time course subgroup of each LAB strain with respect to each chemical species being monitored. NS implies non-significant difference. Results which are bolded and underlined indicate the statistically significant highest values ($p \leq 0.05$)

within the subgroup. Each result for 0th h cultivation time was obtained immediately after inoculum addition and well mixing. The initial pH level before addition of inoculum, which contained slightly produced acids, in each case was 6.00 ± 0.01.

Table S2.3: Detailed kinetics data for five LAB strains cultivation in MsRB medium during 0 – 72 h with respect to $Y_{LA/Glu}$, produced ExVol-PE$_{act}$, produced ExSp-PE$_{act}$, produced InVol-PE$_{act}$, and produced InSp-PE$_{act}$.

**Cultivation time (h)**	**LAB strains**				
	***Lacticaseibacillus casei* TISTR 1500**	***Lactiplantibacillus plantarum* TISTR 877**	***Latilactobacillus sakei* TISTR 890**	***Limosilactobacillus fermentum* TISTR 055**	***Weissella confusa* TISTR 1498**
	**$Y_{LA/Glu}$ (g LA$_{produced}$ / g Glu$_{consumed}$)**				
**0**	n.d.	n.d.	n.d.	n.d.	n.d.
**24**	n.d.	n.d.	n.d.	n.d.	n.d.
**48**	n.d.	n.d.	n.d.	n.d.	n.d.
**72**	n.d.	n.d.	n.d.	n.d.	n.d.
	**Produced ExVol-PE$_{act}$ (U / mL)**				
**0**	0.0000^{D} ± 0.0000	0.0000^{C} ± 0.0000	0.0000^{C} ± 0.0000	0.0000^{D} ± 0.0000	0.0000^{D} ± 0.0000
**24**	0.0127^{C} ± 0.0010	0.0254^{B} ± 0.0010	0.0164^{B} ± 0.0010	0.0244^{C} ± 0.0010	0.0110^{C} ± 0.0010
**48**	0.0280^{B} ± 0.0010	0.0256^{B} ± 0.0010	**0.0224^{A} ± 0.0010**	0.0272^{B} ± 0.0010	0.0271^{B} ± 0.0010
**72**	**0.0302^{A} ± 0.0010**	**0.0415^{A} ± 0.0020**	**0.0242^{A} ± 0.0010**	**0.0317^{A} ± 0.0020**	**0.0364^{A} ± 0.0020**
	**Produced ExSp-PE$_{act}$ (U / mg$_{protein}$)**				
**0**	0.0000^{C} ± 0.0000	0.0000^{C} ± 0.0000	0.0000^{C} ± 0.0000	0.0000^{C} ± 0.0000	0.0000^{D} ± 0.0000
**24**	0.0391^{B} ± 0.0031	0.0636^{B} ± 0.0026	0.0685^{B} ± 0.0043	**0.0664^{A,B} ± 0.0028**	0.0293^{C} ± 0.0027
**48**	**0.0830^{A} ± 0.0030**	0.0622^{B} ± 0.0024	**0.0837^{A} ± 0.0037**	0.0646^{B} ± 0.0024	0.0690^{B} ± 0.0026
**72**	**0.0893^{A} ± 0.0030**	**0.0790^{A} ± 0.0038**	**0.0884^{A} ± 0.0037**	**0.0711^{A} ± 0.0045**	**0.0804^{A} ± 0.0044**
	**Produced InVol-PE$_{act}$ (U / mL)**				
**0**	0.0000^{B} ± 0.0000	0.0000^{C} ± 0.0000	0.0000^{C} ± 0.0000	0.0000^{B} ± 0.0000	0.0000^{C} ± 0.0000
**24**	**0.0211^{A} ± 0.0010**	0.0212^{B} ± 0.0009	0.0182^{B} ± 0.0005	**0.0203^{A} ± 0.0008**	0.0195^{B} ± 0.0006
**48**	**0.0214^{A} ± 0.0008**	**0.0222^{A,B} ± 0.0010**	**0.0215^{A} ± 0.0010**	**0.0208^{A} ± 0.0008**	0.0200^{B} ± 0.0007
**72**	**0.0223^{A} ± 0.0006**	**0.0236^{A} ± 0.0010**	**0.0217^{A} ± 0.0010**	**0.0217^{A} ± 0.0010**	**0.0223^{A} ± 0.0010**
	**Produced InSp-PE$_{act}$ (U / mg$_{protein}$)**				
**0**	0.000^{C} ± 0.000	0.000^{C} ± 0.000	0.000^{C} ± 0.000	0.000^{B} ± 0.000	0.000^{B} ± 0.000
**24**	**1.075^{A} ± 0.053**	**0.696^{A} ± 0.030**	**0.879^{A} ± 0.032**	**0.836^{A} ± 0.035**	**0.517^{A} ± 0.016**
**48**	**1.027^{A} ± 0.046**	**0.663^{A} ± 0.030**	**0.865^{A,B} ± 0.043**	**0.766^{A} ± 0.030**	**0.513^{A} ± 0.018**
**72**	0.660^{B} ± 0.019	0.569^{B} ± 0.025	0.792^{B} ± 0.038	**0.769^{A} ± 0.036**	**0.552^{A} ± 0.027**

Note: The numbers with the same alphabet (A - D) indicate no statistically significant difference ($p > 0.05$) for comparison between time course subgroup of each LAB strain with respect to each type of produced PE$_{act}$. $Y_{LA/Glu}$ was not determined (n.d.)

due to the presence of relatively low [Glu], insignificant consumed [Glu] and produced [LA]. Results which are bolded and underlined indicate the statistically significant highest values ($p \leq 0.05$) within the subgroup. Each result for 0th h cultivation time was obtained immediately after inoculum addition and well mixing.

Table S2.4: Detailed kinetics data scores from Table S2.1 – S2.3 for five LAB strains cultivation in MsRB medium during 0 – 72 h with respect to produced [Pi], mass balance percentage of [IP6] on produced [Pi], via-CD, produced [LA], and $Y_{LA/Glu}$.

Cultivation time (h)	LAB strains				
	Lacticaseibacillus casei TISTR 1500	*Lactiplantibacillus plantarum* TISTR 877	*Latilactobacillus sakei* TISTR 890	*Limosilactobacillus fermentum* TISTR 055	*Weissella confusa* TISTR 1498
**Produced [Pi] scores (Item 1)**					
**0**	$0.00^{D} \pm 0.00$	$0.00^{D} \pm 0.00$	$0.00^{D} \pm 0.00$	$0.00^{D} \pm 0.00$	$0.00^{D} \pm 0.00$
**24**	$56.6^{C} \pm 0.2$	$58.5^{C} \pm 0.2$	$17.0^{C} \pm 0.2$	$24.5^{C} \pm 0.2$	$52.8^{C} \pm 0.2$
**48**	$62.3^{B} \pm 0.2$	$71.7^{B} \pm 0.2$	$28.3^{B} \pm 0.2$	$28.3^{B} \pm 0.2$	$71.7^{B} \pm 0.2$
**72**	**$69.8^{A} \pm 0.2$**	**$100.0^{A} \pm 0.2$**	**$30.2^{A} \pm 0.2$**	**$39.6^{A} \pm 0.2$**	**$86.8^{A} \pm 0.2$**
**Mass balance percentage of [IP6] on [Pi] production scores (Item 2)**					
**0**	**$100.0^{NS} \pm 2.8$**	**$100.0^{NS} \pm 2.8$**	**$100.0^{NS} \pm 2.8$**	**$100.0^{NS} \pm 2.8$**	**$100.0^{NS} \pm 2.8$**
**24**	**$100.0^{NS} \pm 3.4$**	**$100.0^{NS} \pm 3.1$**	**$100.0^{NS} \pm 2.9$**	**$100.0^{NS} \pm 3.1$**	**$100.0^{NS} \pm 3.4$**
**48**	**$100.0^{NS} \pm 2.7$**	**$100.0^{NS} \pm 2.4$**	**$100.0^{NS} \pm 2.4$**	**$100.0^{NS} \pm 2.7$**	**$100.0^{NS} \pm 2.7$**
**72**	**$100.0^{NS} \pm 2.7$**	**$100.0^{NS} \pm 2.4$**	**$100.0^{NS} \pm 2.1$**	**$100.0^{NS} \pm 2.4$**	**$100.0^{NS} \pm 2.4$**
**Via-CD scores (Item 3)**					
**0**	$71.8^{C} \pm 0.9$	$71.5^{C} \pm 1.0$	$72.2^{B} \pm 1.5$	$71.8^{C} \pm 1.1$	$72.0^{C} \pm 1.0$
**24**	$99.0^{B} \pm 0.3$	$97.1^{B} \pm 1.7$	**$98.0^{A} \pm 1.5$**	$92.4^{B} \pm 1.1$	**$99.0^{A,B} \pm 0.7$**
**48**	**$100.0^{A} \pm 0.2$**	**$100.0^{A} \pm 1.7$**	**$100.0^{A} \pm 1.7$**	$93.6^{B} \pm 1.2$	**$100.0^{A} \pm 0.2$**
**72**	$99.0^{B} \pm 0.3$	**$99.0^{A} \pm 2.2$**	**$99.0^{A} \pm 2.2$**	**$97.5^{A} \pm 0.8$**	$97.5^{B} \pm 1.0$
**Produced [LA] scores (Item 4)**					
**0**	n.d.	n.d.	n.d.	n.d.	n.d.
**24**	n.d.	n.d.	n.d.	n.d.	n.d.
**48**	n.d.	n.d.	n.d.	n.d.	n.d.
**72**	n.d.	n.d.	n.d.	n.d.	n.d.
**$Y_{LA/Glu}$ scores (Item 5)**					
**0**	n.d.	n.d.	n.d.	n.d.	n.d.
**24**	n.d.	n.d.	n.d.	n.d.	n.d.
**48**	n.d.	n.d.	n.d.	n.d.	n.d.
**72**	n.d.	n.d.	n.d.	n.d.	n.d.

Note: The numbers with the same alphabet (A – D or NS) indicate no statistically significant difference ($p > 0.05$) for comparison between time course subgroup of each LAB strain with respect to scores of produced [Pi] and via-CD. Scores of $Y_{LA/Glu}$ and produced [LA] were not determined (n.d.) based on provided rationales in footnote of Table S2.3. Results which are bolded and underlined indicate the statistically significant highest values ($p \leq 0.05$) within the subgroup. The maximum score for each subgroup is 100 which is normalised using comparison across all five LAB strains.

Table S2.5: Detailed kinetics data scores from Table S2.3 for five LAB strains cultivation in MsRB medium during 0 – 72 h with respect to produced ExVol-PE$_{act}$, produced ExSp-PE$_{act}$, produced InVol-PE$_{act}$, and produced InSp-PE$_{act}$ as well as overall normalised scores from Table S2.4 – S2.5.

Cultivation time (h)	LAB strains				
	Lacticaseibacillus casei TISTR 1500	*Lactiplantibacillus plantarum* TISTR 877	*Latilactobacillus sakei* TISTR 890	*Limosilactobacillus fermentum* TISTR 055	*Weissella confusa* TISTR 1498
**Produced ExVol-PE$_{act}$ scores (Item 6)**					
**0**	0.00^{D} ± 0.00	0.00^{C} ± 0.00	0.00^{C} ± 0.00	0.00^{D} ± 0.00	0.00^{D} ± 0.00
**24**	30.6^{C} ± 2.4	61.2^{B} ± 2.4	39.5^{B} ± 2.4	58.8^{C} ± 2.4	26.5^{C} ± 2.4
**48**	67.5^{B} ± 2.4	61.7^{B} ± 2.4	**54.0^{A} ± 2.4**	65.5^{B} ± 2.4	65.3^{B} ± 2.4
**72**	**72.8^{A} ± 2.4**	**100.0^{A} ± 4.8**	**58.3^{A} ± 2.4**	**76.4^{A} ± 4.8**	**87.7^{A} ± 4.8**
**Produced ExSp-PE$_{act}$ scores (Item 7)**					
**0**	0.00^{C} ± 0.00	0.00^{C} ± 0.00	0.00^{C} ± 0.00	0.00^{C} ± 0.00	0.00^{D} ± 0.00
**24**	43.8^{B} ± 3.5	71.2^{B} ± 2.9	76.7^{B} ± 4.8	**74.4^{A,B} ± 3.1**	32.8^{C} ± 3.0
**48**	**92.9^{A} ± 3.4**	69.7^{B} ± 2.7	**93.7^{A} ± 4.1**	72.3^{B} ± 2.7	77.3^{B} ± 2.9
**72**	**100.0^{A} ± 3.4**	**88.5^{A} ± 4.3**	**99.0^{A} ± 4.1**	**79.6^{A} ± 5.0**	**90.0^{A} ± 4.9**
**Produced InVol-PE$_{act}$ scores (Item 8)**					
**0**	0.00^{B} ± 0.00	0.00^{C} ± 0.00	0.00^{C} ± 0.00	0.00^{B} ± 0.00	0.00^{C} ± 0.00
**24**	**89.4^{A} ± 4.2**	89.8^{B} ± 3.8	77.1^{B} ± 2.1	**86.0^{A} ± 3.4**	82.6^{B} ± 2.5
**48**	**90.7^{A} ± 3.4**	**94.1^{A,B} ± 4.2**	**91.1^{A} ± 4.2**	**88.1^{A} ± 3.4**	84.7^{B} ± 3.0
**72**	**94.5^{A} ± 2.5**	**100.0^{A} ± 4.2**	**91.9^{A} ± 4.2**	**91.9^{A} ± 4.2**	**94.5^{A} ± 4.2**
**Produced InSp-PE$_{act}$ scores (Item 9)**					
**0**	0.00^{C} ± 0.00	0.00^{C} ± 0.00	0.00^{C} ± 0.00	0.00^{B} ± 0.00	0.00^{B} ± 0.00
**24**	**100.0^{A} ± 4.9**	**64.7^{A} ± 2.8**	**81.8^{A} ± 3.0**	**77.8^{A} ± 3.3**	**48.1^{A} ± 1.5**
**48**	**95.5^{A} ± 4.3**	**61.7^{A} ± 2.8**	**80.5^{A,B} ± 4.0**	**71.3^{A} ± 2.8**	**47.7^{A} ± 1.7**
**72**	61.4^{B} ± 1.8	52.9^{B} ± 2.3	73.7^{B} ± 3.5	**71.5^{A} ± 3.3**	**51.3^{A} ± 2.5**
**Overall normalised scoring to 100 for summation of Item 1 – 9 from Table S2.4 and S2.5**					
**0**	26.8^{C} ± 0.5	26.8^{C} ± 0.5	26.9^{C} ± 0.5	26.8^{C} ± 0.5	26.9^{D} ± 0.2
**24**	81.1^{B} ± 1.3	84.7^{B} ± 1.1	76.5^{B} ± 1.1	80.2^{B} ± 1.1	69.0^{C} ± 0.9
**48**	**95.1^{A} ± 1.2**	87.3^{B} ± 1.1	**85.5^{A} ± 1.3**	81.1^{B} ± 1.0	85.4^{B} ± 0.9
**72**	**93.3^{A} ± 0.9**	**100.0^{A} ± 1.4**	**86.2^{A} ± 1.2**	**86.9^{A} ± 1.4**	**94.9^{A} ± 1.4**
**Summation (D_{Sc})**	**296^{a} ± 2**	**299^{a} ± 2**	275^{b} ± 2	275^{b} ± 2	276^{b} ± 2

Note: The numbers with the same capital alphabet (A - D) indicate no statistically significant difference ($p > 0.05$) for comparison between time course subgroup of each LAB strain with respect to each type of produced PE$_{act}$ score. Results which are bolded and underlined indicate the statistically significant highest values ($p \leq 0.05$) within the subgroup. The maximum score for each subgroup is 100 which is normalised using comparison across all five LAB strains. The summation row at the end of the table represents the summation of overall normalised scores at 0, 24, 48, and 72 h for each LAB strain with the maximum summation of overall normalised score of 400. The statistically significant difference comparsion between data of summation row was carried out at the similar p level with representation by small alphabets (a – b).

Table S2.6: Detailed kinetics parameters for five LAB strains cultivation in MsRB medium during three time intervals between 0 – 72 h with respect to m, $q_{s,Glu}$, $q_{p,Pi}$, and $q_{p,LA}$.

**Cutivation time interval (h)**	**LAB strains**				
	Lacticaseibacillus casei **TISTR 1500**	***Lactiplantibacillus plantarum*** **TISTR 877**	***Latilactobacillus sakei*** **TISTR 890**	***Limosilactobacillus fermentum*** **TISTR 055**	***Weissella confusa*** **TISTR 1498**
**m (× 10^{-2} per h)**					
**0 - 24**	**$1.33^{A} \pm 0.05$**	**$1.26^{A} \pm 0.10$**	**$1.27^{A} \pm 0.10$**	**$1.04^{A} \pm 0.08$**	**$1.32^{A} \pm 0.06$**
**24 - 48**	$0.041^{B} \pm 0.015$*	$0.124^{B} \pm 0.100$*	$0.082^{B} \pm 0.094$*	$0.057^{B} \pm 0.072$*	$0.041^{B} \pm 0.030$*
**48 - 72**	$-0.041^{C} \pm 0.015$*	$-0.041^{B} \pm 0.114$*	$-0.041^{B} \pm 0.114$*	$0.171^{B} \pm 0.062$*	$-0.103^{C} \pm 0.042$*
**$q_{s,Glu}$ (× 10^{-2} g / L / Log(CFU/mL) / h)**					
**0 - 24**	**$-0.005^{A} \pm 0.007$***	**$-0.005^{A} \pm 0.007$***	**$-0.149^{A} \pm 0.048$***	**$-0.189^{A} \pm 0.050$***	**$-0.009^{A} \pm 0.068$***
**24 - 48**	**$-0.004^{A} \pm 0.006$***	**$-0.004^{A} \pm 0.006$***	$-0.008^{B} \pm 0.001$*	$-0.009^{B} \pm 0.001$*	**$-0.004^{A} \pm 0.058$***
**48 - 72**	**$-0.004^{A} \pm 0.006$***	**$-0.008^{A} \pm 0.006$***	$0.000^{C} \pm 0.000$	$-0.004^{C} \pm 0.001$*	**$-0.004^{A} \pm 0.058$***
**$q_{p,Pi}$ (× 10^{-3} g / L / Log(CFU/mL) / h)**					
**0 - 24**	**$1.44^{A} \pm 0.01$**	**$1.50^{A} \pm 0.02$**	**$0.432^{A} \pm 0.007$**	**$0.647^{A} \pm 0.008$**	**$1.34^{A} \pm 0.01$**
**24 - 48**	$0.123^{C} \pm 0.006$	$0.290^{C} \pm 0.007$	$0.248^{B} \pm 0.006$	$0.088^{C} \pm 0.006$	$0.410^{B} \pm 0.006$
**48 - 72**	$0.164^{B} \pm 0.006$	$0.616^{B} \pm 0.010$	$0.041^{C} \pm 0.006$	$0.256^{B} \pm 0.006$	$0.331^{C} \pm 0.006$
**$q_{p,LA}$ (× 10^{-2} g / L / Log(CFU/mL) / h)**					
**0 - 24**	**$0.057^{A} \pm 0.019$***	**$0.029^{A} \pm 0.005$***	$0.000^{NS} \pm 0.000$	**$0.184^{A} \pm 0.005$**	**$0.014^{A} \pm 0.005$**
**24 - 48**	$-0.025^{B} \pm 0.020$*	**$0.016^{A} \pm 0.009$***	$0.000^{NS} \pm 0.000$	$-0.162^{C} \pm 0.005$	**$0.025^{A} \pm 0.006$***
**48 - 72**	$-0.016^{B} \pm 0.017$*	**$0.025^{A} \pm 0.009$***	$0.000^{NS} \pm 0.000$	$0.000^{B} \pm 0.000$*	**$0.016^{A} \pm 0.013$***

Note: The numbers with the same alphabet (A - C) indicate no statistically significant difference ($p > 0.05$) for comparison between time interval subgroup of each LAB strain with respect to each specific rate being examined. NS implies non-significant difference. All negative values were colored violet. The negative values of m indicate the mitigating via-CD as cultivation times progressed while the negative values of $q_{s,Glu}$ represent decreasing [Glu] level with time. The negative values of $q_{p,LA}$ represented degradation of [LA] with time. Results which are bolded and underlined indicate the statistically significant highest values ($p \leq 0.05$) within the subgroup.

* Explanation of the apparent relatively large SE has been clearly explained in footnote of Table S1.6.

Table S2.7: Detailed kinetics parameters scores from Table S2.6 for five LAB strains cultivation in MsRB medium during three time intervals between 0 – 72 h with respect to m, $q_{s,Glu}$, $q_{p,Pi}$, and $q_{p,LA}$ as well as overall normalised scores.

**Cultivation time Interval (h)**	**LAB strains**				
	Lacticaseibacillus casei **TISTR 1500**	***Lactiplantibacillus plantarum*** **TISTR 877**	***Latilactobacillus sakei*** **TISTR 890**	***Limosilactobacillus fermentum*** **TISTR 055**	***Weissella confusa*** **TISTR 1498**
**m scores (Item 10)**					
**0 - 24**	**100.0^{A} ± 3.5**	**95.1^{A} ± 7.27**	**95.3^{A} ± 7.75**	**78.6^{A} ± 5.87**	**99.2^{A} ± 4.4**
**24 - 48**	3.09^{B} ± 1.11*	9.35^{B} ± 7.50*	6.20^{B} ± 7.03*	4.29^{B} ± 5.38*	3.09^{B} ± 2.25*
**48 - 72**	-3.09^{C} ± 1.11*	-3.09^{B} ± 8.58*	-3.09^{B} ± 8.58*	12.8^{B} ± 4.6*	-7.77^{C} ± 3.17*
**$q_{s,Glu}$ scores (Item 11)**					
**0 - 24**	n.d.	n.d.	n.d.	n.d.	n.d.
**24 - 48**	n.d.	n.d.	n.d.	n.d.	n.d.
**48 - 72**	n.d.	n.d.	n.d.	n.d.	n.d.
**$q_{p,Pi}$ scores (Item 12)**					
**0 - 24**	**95.5^{A} ± 0.6**	**100.0^{A} ± 1.0**	**28.8^{A} ± 0.5**	**43.1^{A} ± 0.5**	**89.0^{A} ± 0.7**
**24 - 48**	8.19^{C} ± 0.39	19.3^{C} ± 0.4	16.5^{B} ± 0.4	5.85^{C} ± 0.42	27.3^{B} ± 0.4
**48 - 72**	10.9^{B} ± 0.39	41.0^{B} ± 0.7	2.73^{C} ± 0.39*	17.1^{B} ± 0.4	22.0^{C} ± 0.4
**$q_{p,LA}$ scores (Item 13)**					
**0 - 24**	n.d.	n.d.	n.d.	n.d.	n.d.
**24 - 48**	n.d.	n.d.	n.d.	n.d.	n.d.
**48 - 72**	n.d.	n.d.	n.d.	n.d.	n.d.
**Overall normalised scoring to 100 for summation of Item 10 – 13**					
**0 - 24**	**100.0^{A} ± 1.8**	**99.8^{A} ± 3.8**	**63.4^{A} ± 4.0**	**62.2^{A} ± 3.0**	**96.3^{A} ± 2.3**
**24 – 48**	5.77^{B} ± 0.60*	14.7^{B} ± 3.8*	11.6^{B} ± 3.6*	5.19^{C} ± 2.76*	15.6^{B} ± 1.2
**48 – 72**	4.01^{C} ± 0.60*	19.4^{B} ± 4.4*	-0.18^{C} ± 4.39*	15.3^{B} ± 2.4*	7.28^{C} ± 1.64*
**Summation S2.7 (P_{Sc})**	110^{c} ± 2	**134^{a} ± 7**	74.9^{d} ± 6.9	82.7^{d} ± 4.7	119^{b} ± 3
**Summation S2.5 + S2.7 (D_{Sc} + P_{Sc})**	406^{b} ± 3	**433^{a} ± 7**	350^{d} ± 7	358^{d} ± 5	395^{c} ± 4
**Summation 2 × S2.5 + S2.7 (2D_{Sc} + P_{Sc})**	702^{b} ± 5	**731^{a} ± 8**	625^{d} ± 8	633^{d} ± 6	671^{c} ± 5

Note: The numbers with the same capital alphabet (A - C) indicate no statistically significant difference ($p > 0.05$) for comparison between time interval subgroup of each LAB strain with respect to scoring of each specific rate being examined. Results which are bolded and underlined indicate the statistically significant highest values ($p \le 0.05$) within the subgroup. The maximum score for each subgroup is 100 which is normalised using comparison across all five LAB strains. All negative values were colored violet. $q_{s,Glu}$ and $q_{p,LA}$ scores were not determined (n.d.) based on provided rationale in footnote of Table S2.3. The summation rows at the end of the Table represent the summation of overall normalised scores at 0 - 24, 24 - 48, and 48 - 72 h from Table S2.7 (P_{Sc}); Table S2.5 and S2.7 with equal weighting (D_{Sc} + P_{Sc}), as well as Table S2.5 and S2.7 with double weighting of the former (2D_{Sc} + P_{Sc}) for each LAB strain with the maximum summation of overall normalised score of 300, 400 + 300 = 700,

and 2 × 400 + 300 = 1,100, respectively. The statistically significant difference comparsion between data of these summation rows were carried out at the similar p level with representation by small alphabets (a – d).

* These SE were correctly propagated through mathematical operations, see footnote of Table S1.6 for rationale.

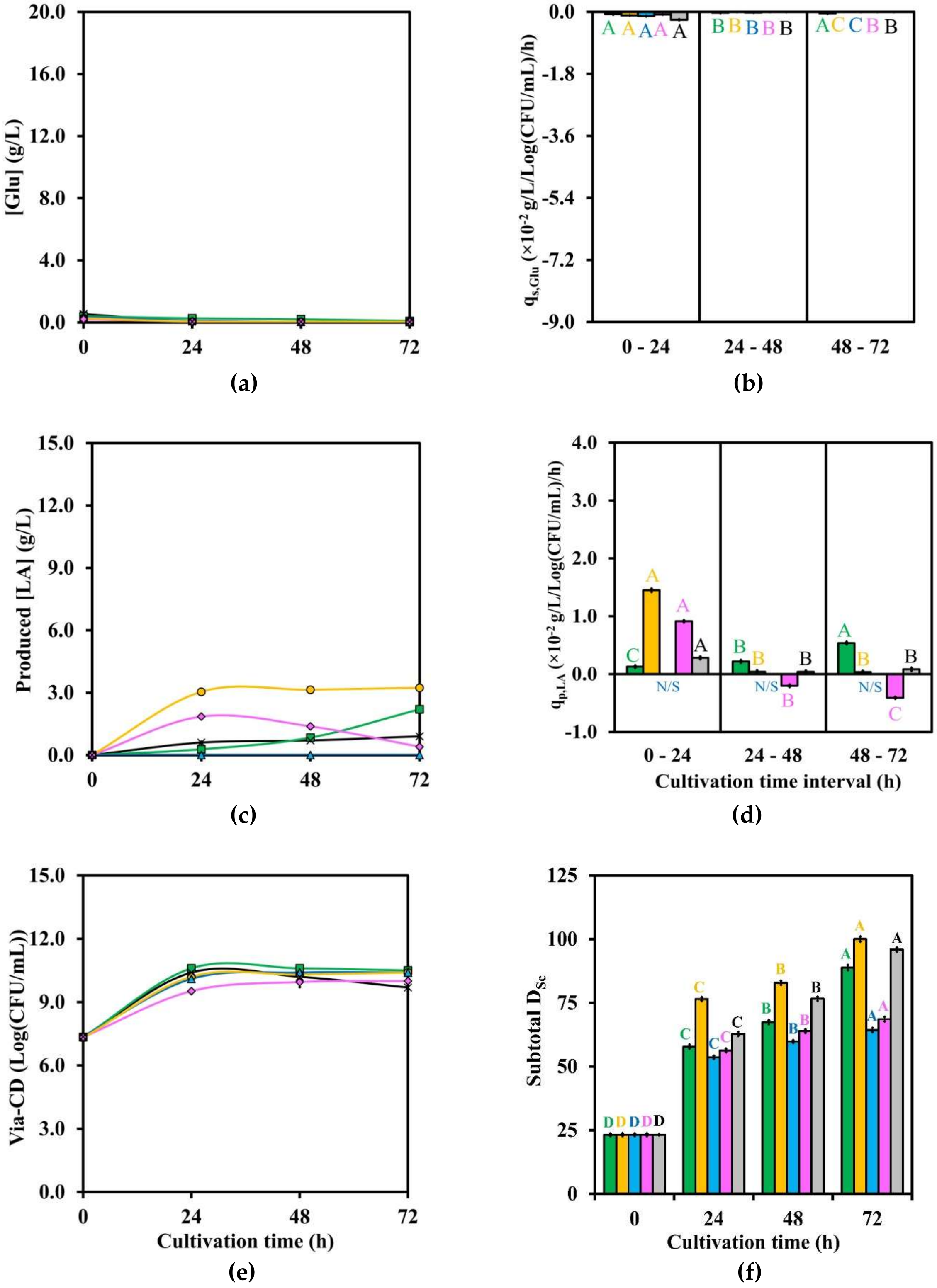

Figure S3: Kinetic data and parameters profiles as well as normalised weighting scores of five LAB strains (TISTR ■ 1500; ● 877; ▲ 890; ◆ 055; × 1498) during 72 h cultivation time in WsRB medium with respect to (a) [Glu]; (b) $q_{s,Glu}$; (c) produced [LA]; (d) $q_{p,LA}$; (e) via-CD; and (f) subtotal D_{Sc}. Each SE was included as an error bar to each data point. The tabulated average and error results values for each LAB strain cultivation with stastistical significant

comparison between time points of (a) were in Supplementary Section B Table S3.1; (b) in Table S3.6; (c) in Table S3.2; (d) in Table S3.6; (e) in Table S3.1; and (f) in Table S3.5. The statistically significant comparison in (b), (d), and (f) were made across three cultivation time intervals or four cultivation time periods for each LAB strain with similar font coloring. The numbers with the same alphabet (A - D) indicated no statistically significant difference ($p > 0.05$).

Table S3.1: Detailed kinetics data for five LAB strains cultivation in WsRB medium during 0 – 72 h with respect to [Glu], [IP6], produced [Pi], mass balance percentage of [IP6] on [Pi] production, and via-CD.

**Cultivation time (h)**	**LAB strains**				
	Lacticaseibacillus casei **TISTR 1500**	***Lactiplantibacillus plantarum*** **TISTR 877**	***Latilactobacillus sakei*** **TISTR 890**	***Limosilactobacillus fermentum*** **TISTR 055**	***Weissella confusa*** **TISTR 1498**
**[Glu] (g/L)**					
**0**	**0.40^{A} ± 0.03**	**0.27^{A} ± 0.01**	**0.35^{A} ± 0.01**	**0.21^{A} ± 0.01**	**0.54^{A} ± 0.03**
**24**	0.26^{B} ± 0.01	0.05^{B} ± < 0.01	0.09^{B} ± < 0.01	0.06^{B} ± < 0.01	0.05^{B} ± < 0.01
**48**	0.20^{C} ± 0.02	0.04^{C} ± < 0.01	0.04^{C} ± < 0.01	0.05^{C} ± < 0.01	0.04^{C} ± < 0.01
**72**	0.09^{D} ± 0.01	0.04^{C} ± < 0.01	0.03^{D} ± < 0.01	0.04^{D} ± < 0.01	0.03^{D} ± < 0.01
**$[IP6]_{overall}$ (g/L)**					
**0**	**6.52^{A} ± 0.15**	**6.52^{A} ± 0.15**	**6.52^{A} ± 0.15**	**6.52^{A} ± 0.15**	**6.52^{A} ± 0.15**
**24**	5.45^{B} ± 0.26	5.54^{B} ± 0.22	**6.29^{A,B} ± 0.20**	**6.35^{A} ± 0.27**	5.22^{B} ± 0.27
**48**	5.26^{B} ± 0.28	5.34^{B,C} ± 0.25	6.19^{B} ± 0.14	**6.11^{A,B} ± 0.21**	4.96^{B} ± 0.21
**72**	5.20^{B} ± 0.37	5.13^{C} ± 0.17	6.14^{B} ± 0.17	5.83^{B} ± 0.25	4.84^{B} ± 0.16
**Produced [Pi] (g/L)**					
**0**	0.00^{D} ± 0.00	0.00^{D} ± 0.00	0.00^{D} ± 0.00	0.00^{D} ± 0.00	0.00^{D} ± 0.00
**24**	0.66^{C} ± < 0.01	0.61^{C} ± < 0.01	0.14^{C} ± < 0.01	0.10^{C} ± < 0.01	0.80^{C} ± < 0.01
**48**	0.78^{B} ± < 0.01	0.72^{B} ± < 0.01	0.20^{B} ± < 0.01	0.25^{B} ± < 0.01	0.96^{B} ± < 0.01
**72**	**0.81^{A} ± < 0.01**	**0.85^{A} ± < 0.01**	**0.23^{A} ± < 0.01**	**0.42^{A} ± < 0.01**	**1.03^{A} ± < 0.01**
**Mass balance percentage of $[IP6]_{overall}$ on [Pi] production (%)**					
**0**	**100.0^{NS} ± 3.4**	**100.0^{NS} ± 3.4**	**100.0^{NS} ± 3.4**	**100.0^{NS} ± 3.4**	**100.0^{NS} ± 3.4**
**24**	**100.0^{NS} ± 4.7**	**100.0^{NS} ± 4.1**	**100.0^{NS} ± 3.9**	**100.0^{NS} ± 4.8**	**100.0^{NS} ± 4.8**
**48**	**100.0^{NS} ± 4.9**	**100.0^{NS} ± 4.6**	**100.0^{NS} ± 3.2**	**100.0^{NS} ± 4.0**	**100.0^{NS} ± 4.0**
**72**	**100.0^{NS} ± 6.1**	**100.0^{NS} ± 3.5**	**100.0^{NS} ± 3.5**	**100.0^{NS} ± 4.6**	**100.0^{NS} ± 3.4**
**Via-CD (Log (CFU/mL))**					
**0**	7.34^{B} ± 0.10	7.31^{B} ± 0.11	7.38^{C} ± 0.14	7.34^{C} ± 0.12	7.36^{C} ± 0.12
**24**	**10.6^{A} ± 0.14**	**10.2^{A} ± 0.14**	10.1^{B} ± 0.05	9.52^{B} ± 0.08	**10.4^{A} ± 0.08**
**48**	**10.6^{A} ± 0.13**	**10.3^{A} ± 0.10**	**10.4^{A} ± 0.07**	**9.95^{A} ± 0.24**	**10.2^{A} ± 0.14**
**72**	**10.5^{A} ± 0.12**	**10.4^{A} ± 0.11**	**10.4^{A} ± 0.11**	**10.0^{A} ± 0.09**	9.69^{B} ± 0.22

Note: $[IP6]_{overall}$ in RB was the combined $[IP6]_{sol}$ and $[IP6]_{in\text{-}sol}$ with assumption of mass balance enclosure. $[IP6]_{sol}$ for each time point for WsRB was between 2.75 – 3.55 g/L. Mass balance percentage of [IP6] on [Pi] production in each data set might be subjected to slight rounding off error. The numbers with the same alphabet (A – D or NS) indicate no statistically significant difference ($p > 0.05$) for comparison between time course subgroup of each LAB strain with respect to each via-CD or chemical species being monitored. Results which are bolded and underlined indicate the statistically significant highest values ($p \leq 0.05$) within the subgroup. Each result for 0th h cultivation time was obtained immediately after inoculum addition and well mixing.

Table S3.2: Detailed kinetics data for five LAB strains cultivation in WsRB medium during 0 – 72 h with respect to produced [LA], produced [formic acid], produced [acetic acid], produced [ethanol], and pH level. Production of butyric acid, succinic acid, 1-propanol, 1,2-propanediol were not detected in all cases.

**Cultivation time (h)**	**LAB strains**				
	Lacticaseibacillus casei **TISTR 1500**	*Lactiplantibacillus plantarum* **TISTR 877**	*Latilactobacillus sakei* **TISTR 890**	*Limosilactobacillus fermentum* **TISTR 055**	*Weissella confusa* **TISTR 1498**
**Produced [LA] (g/L)**					
**0**	$0.00^{D} \pm 0.00$	$0.00^{D} \pm 0.00$	$0.00^{NS} \pm 0.00$	$0.00^{D} \pm 0.00$	$0.00^{D} \pm 0.00$
**24**	$0.28^{C} \pm 0.05$	$3.04^{C} \pm 0.05$	$0.00^{NS} \pm 0.00$	**$1.85^{A} \pm 0.04$**	$0.60^{C} \pm 0.05$
**48**	$0.84^{B} \pm 0.04$	$3.14^{B} \pm 0.04$	$0.00^{NS} \pm 0.00$	$1.38^{B} \pm 0.03$	$0.70^{B} \pm 0.03$
**72**	**$2.20^{A} \pm 0.04$**	**$3.23^{A} \pm 0.04$**	$0.00^{NS} \pm 0.00$	$0.40^{C} \pm 0.03$	**$0.90^{A} \pm 0.06$**
**Produced [formic acid] (g/L)**					
**0**	$0.00^{D} \pm 0.00$	$0.00^{C} \pm 0.00$	$0.00^{D} \pm 0.00$	$0.00^{D} \pm 0.00$	$0.00^{C} \pm 0.00$
**24**	$0.13^{C} \pm 0.04$	$0.45^{B} \pm 0.02$	$1.58^{C} \pm 0.02$	**$0.48^{A} \pm 0.01$**	$0.22^{B} \pm 0.03$
**48**	$0.23^{B} \pm 0.04$	**$0.62^{A} \pm 0.03$**	$1.67^{B} \pm 0.01$	$0.43^{B} \pm 0.02$	**$0.29^{A} \pm 0.01$**
**72**	**$0.46^{A} \pm 0.04$**	**$0.58^{A} \pm 0.05$**	**$1.99^{A} \pm 0.01$**	$0.35^{C} \pm 0.01$	**$0.28^{A} \pm 0.03$**
**Produced [acetic acid] (g/L)**					
**0**	$0.00^{D} \pm 0.00$	$0.00^{D} \pm 0.00$	$0.00^{D} \pm 0.00$	$0.00^{D} \pm 0.00$	$0.00^{C} \pm 0.00$
**24**	$0.31^{C} \pm 0.04$	$0.42^{C} \pm 0.04$	$2.41^{B} \pm 0.07$	$1.13^{C} \pm 0.02$	$1.15^{B} \pm 0.06$
**48**	$1.14^{B} \pm 0.03$	$1.39^{B} \pm 0.04$	**$3.27^{A} \pm 0.10$**	$2.32^{B} \pm 0.01$	$1.20^{B} \pm 0.06$
**72**	**$2.49^{A} \pm 0.06$**	**$1.66^{A} \pm 0.06$**	$1.98^{C} \pm 0.07$	**$2.96^{A} \pm 0.04$**	**$1.39^{A} \pm 0.07$**
**Produced [ethanol] (g/L)**					
**0**	$0.00^{C} \pm 0.00$	$0.00^{C} \pm 0.00$	$0.00^{D} \pm 0.00$	$0.00^{C} \pm 0.00$	$0.00^{D} \pm 0.00$
**24**	$0.00^{C} \pm 0.00$	$0.00^{C} \pm 0.00$	$0.34^{C} \pm 0.02$	$0.35^{B} \pm 0.02$	$0.36^{C} \pm 0.05$
**48**	$0.49^{B} \pm 0.01$	$0.56^{B} \pm 0.01$	$0.48^{B} \pm 0.02$	**$0.46^{A} \pm 0.03$**	**$0.61^{A} \pm 0.05$**
**72**	**$0.62^{A} \pm 0.05$**	**$0.68^{A} \pm 0.02$**	**$0.56^{A} \pm 0.02$**	**$0.49^{A} \pm 0.02$**	$0.46^{B} \pm 0.03$
**pH level**					
**0**	**$5.91^{A} \pm 0.01$**	**$5.99^{A} \pm 0.01$**	**$5.92^{A} \pm 0.01$**	**$5.73^{A} \pm 0.01$**	**$5.87^{A} \pm 0.01$**
**24**	$5.30^{B} \pm 0.01$	$5.43^{B} \pm 0.01$	$5.51^{B} \pm 0.01$	$5.39^{B} \pm 0.01$	$5.64^{B} \pm 0.01$
**48**	$4.83^{C} \pm 0.01$	$4.96^{C} \pm 0.02$	$5.14^{C} \pm 0.01$	$4.96^{C} \pm 0.01$	$4.95^{C} \pm 0.01$
**72**	$4.29^{D} \pm 0.01$	$4.16^{D} \pm 0.01$	$4.83^{D} \pm 0.01$	$4.85^{D} \pm 0.01$	$4.86^{D} \pm 0.01$

Note: The numbers with the same alphabet (A - D) indicate no statistically significant difference ($p > 0.05$) for comparison between time course subgroup of each LAB strain with respect to each chemical species being monitored. Results which are bolded and underlined indicate the statistically significant highest values ($p \leq 0.05$) within the subgroup. Each result for 0th h

cultivation time was obtained immediately after inoculum addition and well mixing. The initial pH level before addition of inoculum, which contained slightly produced acids, in each case was 6.00 ± 0.01.

Table S3.3: Detailed kinetics data for five LAB strains cultivation in WsRB medium during 0 – 72 h with respect to $Y_{LA/Glu}$, produced ExVol-PE_{act}, produced ExSp-PE_{act}, produced InVol-PE_{act}, and produced InSp-PE_{act}.

Cultivation time (h)	LAB strains				
	Lacticaseibacillus casei TISTR 1500	*Lactiplantibacillus plantarum* TISTR 877	*Latilactobacillus sakei* TISTR 890	*Limosilactobacillus fermentum* TISTR 055	*Weissella confusa* TISTR 1498
	**$Y_{LA/Glu}$ (g $LA_{produced}$ / g $Glu_{consumed}$)**				
**0**	n.d.	n.d.	n.d.	n.d.	n.d.
**24**	n.d.	n.d.	n.d.	n.d.	n.d.
**48**	n.d.	n.d.	n.d.	n.d.	n.d.
**72**	n.d.	n.d.	n.d.	n.d.	n.d.
	**Produced ExVol-PE_{act} (U / mL)**				
**0**	0.0000^{D} ± 0.0000	0.0000^{D} ± 0.0000	0.0000^{B} ± 0.0000	0.0000^{C} ± 0.0000	0.0000^{D} ± 0.0000
**24**	0.0105^{C} ± 0.0010	0.0252^{C} ± 0.0010	**0.0248^{A} ± 0.0010**	0.0160^{B} ± 0.0010	0.0100^{C} ± 0.0010
**48**	0.0229^{B} ± 0.0010	0.0304^{B} ± 0.0010	**0.0264^{A} ± 0.0010**	**0.0291^{A} ± 0.0010**	0.0371^{B} ± 0.0020
**72**	**0.0424^{A} ± 0.0020**	**0.0532^{A} ± 0.0030**	**0.0264^{A} ± 0.0020**	**0.0293^{A} ± 0.0020**	**0.0753^{A} ± 0.0020**
	**Produced ExSp-PE_{act} (U / $mg_{protein}$)**				
**0**	0.0000^{D} ± 0.0000	0.0000^{D} ± 0.0000	0.0000^{C} ± 0.0000	0.0000^{C} ± 0.0000	0.0000^{D} ± 0.0000
**24**	0.0091^{C} ± 0.0009	0.0258^{C} ± 0.0010	0.0213^{B} ± 0.0009	0.0149^{B} ± 0.0009	0.0082^{C} ± 0.0008
**48**	0.0208^{B} ± 0.0009	0.0328^{B} ± 0.0011	**0.0238^{A} ± 0.0009**	**0.0277^{A} ± 0.0010**	0.0314^{B} ± 0.0017
**72**	**0.0397^{A} ± 0.0019**	**0.0593^{A} ± 0.0033**	**0.0257^{A} ± 0.0019**	**0.0290^{A} ± 0.0020**	**0.0644^{A} ± 0.0017**
	**Produced InVol-PE_{act} (U / mL)**				
**0**	0.0000^{D} ± 0.0000	0.0000^{D} ± 0.0000	0.0000^{D} ± 0.0000	0.0000^{C} ± 0.0000	0.0000^{D} ± 0.0000
**24**	0.0206^{C} ± 0.0003	0.0237^{C} ± 0.0003	0.0205^{C} ± 0.0002	0.0194^{B} ± 0.0001	0.0251^{C} ± 0.0001
**48**	0.0215^{B} ± 0.0001	0.0248^{B} ± 0.0003	0.0210^{B} ± 0.0001	0.0196^{B} ± 0.0004	0.0269^{B} ± 0.0001
**72**	**0.0283^{A} ± 0.0001**	**0.0305^{A} ± 0.0001**	**0.0238^{A} ± 0.0003**	**0.0255^{A} ± 0.0002**	**0.0321^{A} ± 0.0001**
	**Produced InSp-PE_{act} (U / $mg_{protein}$)**				
**0**	0.000^{D} ± 0.000	0.000^{D} ± 0.000	0.000^{D} ± 0.000	0.000^{D} ± 0.000	0.000^{D} ± 0.000
**24**	0.128^{C} ± 0.002	0.138^{C} ± 0.002	0.119^{C} ± 0.001	0.111^{C} ± 0.001	0.135^{C} ± 0.001
**48**	0.136^{B} ± 0.001	0.161^{B} ± 0.002	0.183^{B} ± 0.001	0.142^{B} ± 0.003	0.149^{B} ± 0.001
**72**	**0.217^{A} ± 0.001**	**0.206^{A} ± 0.002**	**0.225^{A} ± 0.003**	**0.202^{A} ± 0.002**	**0.182^{A} ± 0.001**

Note: The numbers with the same alphabet (A - D) indicate no statistically significant difference ($p > 0.05$) for comparison between time course subgroup of each LAB strain with respect to each type of produced PE_{act}. $Y_{LA/Glu}$ was not determined (n.d.)

due to the presence of relatively low [Glu] and insignificantly consumed [Glu]. Results which are bolded and underlined indicate the statistically significant highest values ($p \leq 0.05$) within the subgroup. Each result for 0th h cultivation time was obtained immediately after inoculum addition and well mixing.

Table S3.4: Detailed kinetics data scores from Table S3.1 – S3.3 for five LAB strains cultivation in WsRB medium during 0 – 72 h with respect to produced [Pi], mass balance percentage of [IP6] on [Pi] production, via-CD, produced [LA], and $Y_{LA/Glu}$.

**Cultivation time (h)**	**LAB strains**				
	***Lacticaseibacillus casei* TISTR 1500**	***Lactiplantibacillus plantarum* TISTR 877**	***Latilactobacillus sakei* TISTR 890**	***Limosilactobacillus fermentum* TISTR 055**	***Weissella confusa* TISTR 1498**
**Produced [Pi] scores (Item 1)**					
**0**	0.00^{D} ± 0.00	0.00^{D} ± 0.00	0.00^{D} ± 0.00	0.00^{D} ± 0.00	0.00^{D} ± 0.00
**24**	64.1^{C} ± 0.1	59.2^{C} ± 0.1	13.6^{C} ± 0.1	9.71^{C} ± 0.10	77.7^{C} ± 0.1
**48**	75.7^{B} ± 0.1	69.9^{B} ± 0.1	19.4^{B} ± 0.1	24.3^{B} ± 0.1	93.2^{B} ± 0.1
**72**	**78.6^{A} ± 0.1**	**82.5^{A} ± 0.1**	**22.3^{A} ± 0.1**	**40.8^{A} ± 0.1**	**100.0^{A} ± 0.1**
**Mass balance percentage of [IP6] on [Pi] production scores (Item 2)**					
**0**	**100.0^{NS} ± 3.4**	**100.0^{NS} ± 3.4**	**100.0^{NS} ± 3.4**	**100.0^{NS} ± 3.4**	**100.0^{NS} ± 3.4**
**24**	**100.0^{NS} ± 4.7**	**100.0^{NS} ± 4.1**	**100.0^{NS} ± 3.9**	**100.0^{NS} ± 4.8**	**100.0^{NS} ± 4.8**
**48**	**100.0^{NS} ± 4.9**	**100.0^{NS} ± 4.6**	**100.0^{NS} ± 3.2**	**100.0^{NS} ± 4.0**	**100.0^{NS} ± 4.0**
**72**	**100.0^{NS} ± 6.1**	**100.0^{NS} ± 3.5**	**100.0^{NS} ± 3.5**	**100.0^{NS} ± 4.6**	**100.0^{NS} ± 3.4**
**Via-CD scores (Item 3)**					
**0**	69.2^{C} ± 1.7	69.0^{B} ± 1.0	69.6^{C} ± 1.3	69.2^{C} ± 1.1	69.4^{C} ± 1.1
**24**	**100.0^{A} ± 1.3**	**96.2^{A} ± 1.3**	95.3^{B} ± 0.5	89.8^{B} ± 0.8	**98.1^{A} ± 0.8**
**48**	**100.0^{A} ± 1.2**	**97.2^{A} ± 0.9**	**98.1^{A} ± 0.7**	**93.9^{A} ± 2.3**	**96.2^{A} ± 1.3**
**72**	99.1^{B} ± 1.1	**98.1^{A} ± 1.0**	**98.1^{A} ± 1.0**	**94.3^{A} ± 0.8**	91.4^{B} ± 2.1
**Produced [LA] scores (Item 4)**					
**0**	0.00^{D} ± 0.00	0.00^{D} ± 0.00	0.00^{NS} ± 0.00	0.00^{D} ± 0.00	0.00^{D} ± 0.00
**24**	8.67^{C} ± 1.55	94.1^{C} ± 1.6	0.00^{NS} ± 0.00	**57.3^{A} ± 1.2**	18.6^{C} ± 1.6
**48**	26.0^{B} ± 1.2	97.2^{B} ± 1.2	0.00^{NS} ± 0.00	42.7^{B} ± 0.9	21.7^{B} ± 0.9
**72**	**68.1^{A} ± 1.2**	**100.0^{A} ± 1.2**	0.00^{NS} ± 0.00	12.4^{C} ± 0.9	**27.9^{A} ± 1.9**
**$Y_{LA/Glu}$ scores (Item 5)**					
**0**	n.d.	n.d.	n.d.	n.d.	n.d.
**24**	n.d.	n.d.	n.d.	n.d.	n.d.
**48**	n.d.	n.d.	n.d.	n.d.	n.d.
**72**	n.d.	n.d.	n.d.	n.d.	n.d.

Note: The numbers with the same alphabet (A - D) indicate no statistically significant difference ($p > 0.05$) for comparison between time course subgroup of each LAB strain with respect to scores of each chemical species and via-CD. NS implies non-significant difference. Scores of $Y_{LA/Glu}$ was not determined (n.d.) based on provided rationales in footnote of Table S3.3. Results which are bolded and underlined indicate the statistically significant highest values ($p \leq 0.05$) within the subgroup. The maximum score for each subgroup is 100 which is normalised using comparison across all five LAB strains.

Table S3.5: Detailed kinetics data scores from Table S3.3 for five LAB strains cultivation in WsRB medium during 0 – 72 h with respect to produced ExVol-PE$_{act}$, produced ExSp-PE$_{act}$, produced InVol-PE$_{act}$, and produced InSp-PE$_{act}$ as well as overall normalised scores from Table S3.4 – S3.5.

**Cultivation time (h)**	**LAB strains**				
	Lacticaseibacillus casei **TISTR 1500**	***Lactiplantibacillus plantarum*** **TISTR 877**	***Latilactobacillus sakei*** **TISTR 890**	***Limosilactobacillus fermentum*** **TISTR 055**	***Weissella confusa*** **TISTR 1498**
**Produced ExVol-PE$_{act}$ scores (Item 6)**					
**0**	0.00^{D} ± 0.00	0.00^{D} ± 0.00	0.00^{B} ± 0.00	0.00^{C} ± 0.00	0.00^{D} ± 0.00
**24**	13.9^{C} ± 1.3	33.5^{C} ± 1.3	**32.9^{A} ± 1.3**	21.1^{B} ± 1.3	13.3^{C} ± 1.3
**48**	30.4^{B} ± 1.3	40.4^{B} ± 1.3	**35.1^{A} ± 1.3**	**38.6^{A} ± 1.3**	49.3^{B} ± 2.7
**72**	**56.3^{A} ± 2.7**	**70.7^{A} ± 4.0**	**35.1^{A} ± 2.7**	**38.9^{A} ± 2.7**	**100.0^{A} ± 2.7**
**Produced ExSp-PE$_{act}$ scores (Item 7)**					
**0**	0.00^{D} ± 0.00	0.00^{D} ± 0.00	0.00^{C} ± 0.00	0.00^{C} ± 0.00	0.00^{D} ± 0.00
**24**	14.1^{C} ± 1.4	40.1^{C} ± 1.3	33.1^{B} ± 1.4	23.1^{B} ± 1.4	12.7^{C} ± 1.2
**48**	32.3^{B} ± 1.4	50.9^{B} ± 1.7	**37.0^{A} ± 1.4**	**43.0^{A} ± 1.6**	48.8^{B} ± 2.6
**72**	**61.6^{A} ± 3.0**	**92.1^{A} ± 5.1**	**39.9^{A} ± 3.0**	**45.0^{A} ± 3.1**	**100.0^{A} ± 2.6**
**Produced InVol-PE$_{act}$ scores (Item 8)**					
**0**	0.00^{D} ± 0.00	0.00^{D} ± 0.00	0.00^{D} ± 0.00	0.00^{C} ± 0.00	0.00^{D} ± 0.00
**24**	64.2^{C} ± 0.9	73.8^{C} ± 0.9	63.9^{C} ± 0.6	60.4^{B} ± 0.3	78.2^{C} ± 0.3
**48**	67.0^{B} ± 0.3	77.3^{B} ± 0.9	65.4^{B} ± 0.3	61.1^{B} ± 1.2	83.8^{B} ± 0.3
**72**	**88.2^{A} ± 0.3**	**95.0^{A} ± 0.3**	**74.1^{A} ± 0.9**	**79.4^{A} ± 0.6**	**100.0^{A} ± 0.3**
**Produced InSp-PE$_{act}$ scores (Item 9)**					
**0**	0.00^{D} ± 0.00	0.00^{D} ± 0.00	0.00^{D} ± 0.00	0.00^{D} ± 0.00	0.00^{D} ± 0.00
**24**	56.9^{C} ± 0.9	61.3^{C} ± 0.9	52.9^{C} ± 0.4	49.3^{C} ± 0.4	60.0^{C} ± 0.4
**48**	60.4^{B} ± 0.4	71.6^{B} ± 0.9	81.3^{B} ± 0.4	63.1^{B} ± 1.3	66.2^{B} ± 0.4
**72**	**96.4^{A} ± 0.4**	**91.6^{A} ± 0.9**	**100.0^{A} ± 1.3**	**89.8^{A} ± 1.3**	**80.9^{A} ± 0.4**
**Overall normalised scoring to 100 for summation of Item 1 – 9 from Table S3.4 and S3.5**					
**0**	23.2^{D} ± 0.5	23.2^{D} ± 0.5	23.2^{D} ± 0.5	23.2^{D} ± 0.5	23.2^{D} ± 0.5
**24**	57.8^{C} ± 0.8	76.5^{C} ± 0.7	53.6^{C} ± 0.6	56.3^{C} ± 0.7	62.8^{C} ± 0.8
**48**	67.4^{B} ± 0.8	82.8^{B} ± 0.8	59.8^{B} ± 0.5	63.9^{B} ± 0.7	76.6^{B} ± 0.8
**72**	**88.8^{A} ± 1.0**	**100.0^{A} ± 1.0**	**64.3^{A} ± 0.8**	**68.6^{A} ± 0.9**	**95.9^{A} ± 0.8**
**Summation (D_{Sc})**	237^{c} ± 2	**282^{a} ± 2**	201^{e} ± 1	212^{d} ± 1	259^{b} ± 1

Note: The numbers with the same capital alphabet (A - D) indicate no statistically significant difference ($p > 0.05$) for comparison between time course subgroup of each LAB strain with respect to each type of produced PE$_{act}$ score. Results which are bolded and underlined indicate the statistically significant highest values ($p \leq 0.05$) within the subgroup. The maximum score for each subgroup is 100 which is normalised using comparison across all five LAB strains. The summation row at the end of the table represents the summation of overall normalised scores at 0, 24, 48, and 72 h for each LAB strain with the maximum summation of overall normalised score of 400. The statistically significant difference comparsion between data of summation row was carried out at the similar p level with representation by small alphabets (a – e).

Table S3.6: Detailed kinetics parameters for five LAB strains cultivation in WsRB medium during three time intervals between 0 – 72 h with respect to m, $q_{s,Glu}$, $q_{p,Pi}$, and $q_{p,LA}$.

**Cutivation time interval (h)**	**LAB strains**				
	Lacticaseibacillus casei **TISTR 1500**	***Lactiplantibacillus plantarum*** **TISTR 877**	***Latilactobacillus sakei*** **TISTR 890**	***Limosilactobacillus fermentum*** **TISTR 055**	***Weissella confusa*** **TISTR 1498**
**m (× 10^{-2} per h)**					
**0 - 24**	**1.51^{A} ± 0.08**	**1.38^{A} ± 0.08**	**1.30^{A} ± 0.07**	**1.08^{A} ± 0.07**	**1.43^{A} ± 0.07**
**24 - 48**	0.000^{B} ± 0.000	0.041^{B} ± 0.070*	0.122^{B} ± 0.035*	0.184^{B} ± 0.108*	-0.081^{B} ± 0.065*
**48 - 72**	-0.039^{B} ± 0.070*	0.040^{B} ± 0.060*	0.000^{C} ± 0.000	0.021^{B} ± 0.108*	-0.214^{B} ± 0.109*
**$q_{s,Glu}$ (× 10^{-2} g / L / Log(CFU/mL) / h)**					
**0 - 24**	**-0.065^{A} ± 0.015***	**-0.105^{A} ± 0.005**	**-0.124^{A}± 0.005**	**-0.074^{A} ± 0.005**	**-0.230^{A} ± 0.014***
**24 - 48**	-0.024^{B} ± 0.009*	-0.004^{B} ± 0.001*	-0.020^{B} ± 0.001	-0.004^{B} ± 0.001*	-0.004^{B} ± 0.001*
**48 - 72**	**-0.043^{A} ± 0.009***	0.000^{C} ± 0.000	-0.004^{C} ± 0.001*	-0.004^{B} ± 0.001*	-0.004^{B} ± 0.001*
**$q_{p,Pi}$ (× 10^{-3} g / L / Log(CFU/mL) / h)**					
**0 - 24**	**3.07^{A} ± 0.03**	**2.90^{A} ± 0.03**	**0.667^{A} ± 0.007**	0.494^{C} ± 0.006	**3.75^{A} ± 0.03**
**24 - 48**	0.472^{B} ± 0.007	0.447^{C} ± 0.007	0.244^{B} ± 0.006	0.642^{B} ± 0.010	0.647^{B} ± 0.008
**48 - 72**	0.118^{C} ± 0.006	0.523^{B} ± 0.007	0.120^{C} ± 0.006	**0.710^{A} ± 0.011**	0.293^{C} ± 0.007
**$q_{p,LA}$ (× 10^{-2} g / L / Log(CFU/mL) / h)**					
**0 - 24**	0.130^{C} ± 0.023*	**1.45^{A} ± 0.03**	0.000^{NS} ± 0.000	**0.914^{A} ± 0.021**	**0.282^{A} ± 0.024**
**24 - 48**	0.220^{B} ± 0.025*	0.041^{B} ± 0.026*	0.000^{NS} ± 0.000	-0.201^{B} ± 0.022*	0.040^{B} ± 0.024*
**48 - 72**	**0.537^{A} ± 0.023**	0.036^{B} ± 0.023*	0.000^{NS} ± 0.000	-0.409^{C} ± 0.018	0.084^{B} ± 0.028*

Note: The numbers with the same alphabet (A - C) indicate no statistically significant difference ($p > 0.05$) for comparison between time interval subgroup of each LAB strain with respect to each specific rate being examined. NS implies non-significant difference. All negative values were colored violet. The negative values of m indicate the mitigating via-CD as cultivation times progressed while the negative values of $q_{s,Glu}$ represent decreasing [Glu] level with time. The negative values of $q_{p,LA}$ represented degradation of [LA] with time. Results which are bolded and underlined indicate the statistically significant highest values ($p \leq 0.05$) within the subgroup.

* Explanation of the apparent relatively large SE has been clearly explained in footnote of Table S1.6.

Table S3.7: Detailed kinetics parameters scores from Table S3.6 for five LAB strains cultivation in WsRB medium during three time intervals between 0 – 72 h with respect to m, $q_{s,Glu}$, $q_{p,Pi}$, and $q_{p,LA}$ as well as overall normalised scores.

**Cultivation time Interval (h)**	**LAB strains**				
	***Lacticaseibacillus casei* TISTR 1500**	***Lactiplantibacillus plantarum* TISTR 877**	***Latilactobacillus sakei* TISTR 890**	***Limosilactobacillus fermentum* TISTR 055**	***Weissella confusa* TISTR 1498**
**m scores (Item 10)**					
**0 - 24**	**100.0^{A} ± 5.4**	**90.8^{A} ± 5.7**	**85.6^{A} ± 4.7**	**71.2^{A} ± 4.8**	**94.2^{A} ± 4.5**
**24 - 48**	0.00^{B} ± 0.00	2.68^{B} ± 4.62*	8.05^{B} ± 2.31*	12.2^{B} ± 7.2*	-5.34^{B} ± 4.31*
**48 - 72**	-2.61^{B} ± 4.61*	2.66^{B} ± 3.95*	0.00^{C} ± 0.00	1.38^{B} ± 7.07*	-14.1^{B} ± 7.2*
**$q_{s,Glu}$ scores (Item 11)**					
**0 - 24**	n.d.	n.d.	n.d.	n.d.	n.d.
**24 - 48**	n.d.	n.d.	n.d.	n.d.	n.d.
**48 - 72**	n.d.	n.d.	n.d.	n.d.	n.d.
**$q_{p,Pi}$ scores (Item 12)**					
**0 - 24**	**81.7^{A} ± 0.8**	**77.3^{A} ± 0.8**	**17.8^{A} ± 0.2**	13.2^{C} ± 0.2	**100.0^{A} ± 0.8**
**24 - 48**	12.6^{B} ± 0.2	11.9^{C} ± 0.2	6.50^{B} ± 0.16	17.1^{B} ± 0.3	17.2^{B} ± 0.2
**48 - 72**	3.16^{C} ± 0.15	13.9^{B} ± 0.2	3.20^{C} ± 0.15	**18.9^{A} ± 0.3**	7.81^{C} ± 0.19
**$q_{p,LA}$ scores (Item 13)**					
**0 - 24**	8.99^{C} ± 1.61*	**100.0^{A} ± 1.9**	0.000^{NS} ± 0.000	**63.2^{A} ± 1.5**	**19.5^{A} ± 1.6**
**24 - 48**	15.2^{B} ± 1.8*	2.81^{B} ± 1.80*	0.000^{NS} ± 0.000	-13.9^{B} ± 1.5*	2.80^{B} ± 1.63*
**48 - 72**	**37.1^{A} ± 1.6**	2.50^{B} ± 1.57*	0.000^{NS} ± 0.000	-28.3^{C} ± 1.3	5.79^{B} ± 1.94*
**Overall normalised scoring to 100 for summation of Item 10 – 13**					
**0 - 24**	**71.1^{A} ± 2.1**	**100.0^{A} ± 2.3**	**38.6^{A} ± 1.8**	**55.0^{A} ± 1.9**	**79.7^{A} ± 1.8**
**24 – 48**	10.4^{C} ± 0.7	6.49^{B} ± 1.85*	5.43^{B} ± 0.86*	5.73^{B} ± 2.73*	5.48^{B} ± 1.72*
**48 – 72**	14.0^{B} ± 1.8*	7.12^{B} ± 1.59*	1.19^{C} ± 0.06	-2.98^{C} ± 2.68*	-0.19^{C} ± 2.79*
**Summation S3.7 (P_{Sc})**	95.5^{b} ± 2.9	**114^{a} ± 3**	45.2^{e} ± 2.0	57.8^{d} ± 4.2	85.0^{c} ± 3.7
**Summation S3.5 + S3.7 (D_{Sc} + P_{Sc})**	333^{c} ± 3	**396^{a} ± 4**	246^{e} ± 2	270^{d} ± 4	344^{b} ± 4
**Summation 2 × S3.5 + S3.7 (2D_{Sc} + P_{Sc})**	570^{c} ± 4	**678^{a} ± 5**	447^{e} ± 3	482^{d} ± 5	602^{b} ± 5

Note: The numbers with the same capital alphabet (A - C) indicate no statistically significant difference ($p > 0.05$) for comparison between time interval subgroup of each LAB strain with respect to scoring of each specific rate being examined. Results which are bolded and underlined indicate the statistically significant highest values ($p \leq 0.05$) within the subgroup. The maximum score for each subgroup is 100 which is normalised using comparison across all five LAB strains. All negative values were colored violet. $q_{s,Glu}$ was not determined (n.d.) based on provided rationale in footnote of Table S3.3. The summation rows at the end of the Table represent the summation of overall normalised scores at 0 - 24, 24 - 48, and 48 - 72 h from Table S3.7 (P_{Sc}); Table S3.5 and S3.7 with equal weighting (D_{Sc} + P_{Sc}), as well as Table S3.5 and S3.7 with double weighting of the former (2D_{Sc} + P_{Sc}) for each LAB strain with the maximum summation of overall normalised score of 300, 400 + 300 = 700, and 2 × 400 + 300 = 1,100,

respectively. The statistically significant difference comparsion between data of these summation rows were carried out at the similar p level with representation by small alphabets (a – d).

* These SE were correctly propagated through mathematical operations, see footnote of Table S3.6 for rationale.
